# A Co-attention Mechanism of Jointly Capturing Sequence and Structure Features for Signal Peptide Prediction

**DOI:** 10.34133/csbj.0120

**Published:** 2026-07-08

**Authors:** Zhixuan Piao, Yuan Liu, Xiaoyong Pan, Hong-Bin Shen

**Affiliations:** Institute of Image Processing and Pattern Recognition, Shanghai Jiao Tong University, and Key Laboratory of System Control and Information Processing, Ministry of Education of China, Shanghai 200240, China.

## Abstract

Signal peptides are short amino acid sequences at the N-terminus of proteins that serve as subcellular localization signals, directing proteins to specific compartments such as mitochondria, chloroplasts, or the secretory pathway. Although many recent predictors based on protein language models have achieved strong performance, most of them rely primarily on sequence information and do not explicitly incorporate structural cues. Here, we propose Signal-3L 4.0, a dual-path encoder framework that combines pretrained protein representations with task-specific sequence and structural modeling for signal peptide prediction, and the 2 modalities are fused via a VisualBERT-style co-attention module. To alleviate the impact of the long-tail distribution across organism groups and signal peptide types, we further employ a class-balanced label-distribution-aware margin loss. Benchmark results show that Signal-3L 4.0 achieves improved signal peptide classification performance and competitive or better cleavage-site prediction performance compared with SignalP 6.0, with particularly superior performance in several low-resource or challenging settings. Signal-3L 4.0 is freely available for academic use as a web server at http://www.csbio.sjtu.edu.cn/bioinf/Signal-3L/ and as open-source code at https://github.com/chandlevier/Signal-3L-4.0.

## Highlights

• Signal-3L 4.0 integrates sequence and structure via a VisualBERT-style co-attention and protein language model.
• Class-balanced label-distribution-aware margin loss improves performance on rare signal peptide types.
• Signal-3L 4.0 enhances signal peptide prediction performance, especially in Archaea and Sec/SPII categories.
• The freely available Signal-3L 4.0 web server and code enable community-wide application.

## Graphical Abstract

**Figure:**
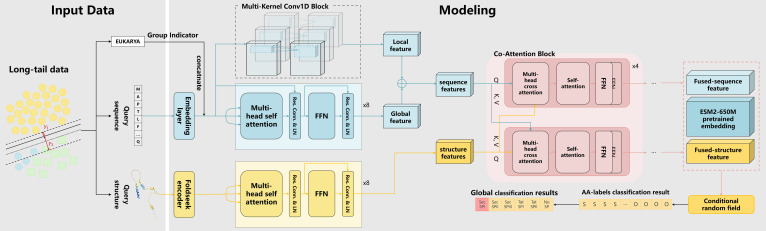

## Introduction

Secreted signal peptides (SPs), also known as signal peptides, are specific amino acid sequences located at the N-terminus of proteins that function similarly to postal codes. They serve to designate the target destination of a protein and direct it into the secretory pathway. Once the protein reaches the secretory pathway, the signal peptide region is cleaved by the corresponding signal peptidase, releasing the mature protein to perform its function in a specific cellular compartment [1,2]. Signal peptides are typically shorter than 70 amino acids in length and generally consist of a positively charged N-region, a highly hydrophobic H-region, and a polar C-region [3]. Signal peptides play a crucial role in various fields, including recombinant protein production and disease diagnosis. Therefore, accurately determining the cleavage sites (CSs) of known signal peptides, as well as discovering novel signal peptides, is of substantial biological and clinical importance [4].

The task of signal peptide prediction primarily consists of 2 aspects: (a) determining whether a given protein contains a signal peptide and, if so, identifying its specific type, namely, Sec/SPI (translocated via the Sec pathway and cleaved by signal peptidase I), Sec/SPII, Sec/SPIII, Tat/SPI (translocated via the Tat pathway and cleaved by signal peptidase I), and Tat/SPII, covering 5 distinct signal peptide categories, and (b) accurately locating the enzymatic CS of the signal peptide in the protein [5,6].

Prior to 2017, most approaches relied on traditional machine learning algorithms or simple neural networks. For instance, tools such as SignalP 1.0 [7,8], SignalP 3.0 [9], LipoP [10], TatP [11], SPEPlip [12], and PredSL [13] were all based on artificial neural networks, and Signal-3L 2.0 [14] primarily used support vector machines for classification. With the advancement of deep learning technologies, an increasing number of approaches have adopted deeper model architectures, such as convolutional neural networks (CNNs) [15], bidirectional long short-term memory networks (BiLSTMs) [16], graph neural networks [17], and protein language models [18], to improve the performance of signal peptide prediction. Representative deep-learning-based predictors include DeepSig [19], TargetP 2.0 [20], SignalP 5.0 [21], and SignalP 6.0 [5,6], Signal-3L 3.0 [22], PEFT-SP [23], TSignal [24], and USPNet [25,26].

Among these methods, SignalP 6.0 is the first approach to directly employ the protein language model ProtBERT in place of the conventional BiLSTM and CNN hybrid architecture. By leveraging ProtBERT, the model captures deeper sequence-level features (such as evolutionary information), markedly improving the performance in low-data scenarios. It also demonstrates strong generalization capabilities and accurately makes predictions for the protein sequences that differ substantially from the training data. TSignal introduces an innovative transformer-like encoder–decoder architecture that reformulates the task of signal peptide CS prediction as a machine translation problem, rather than a conventional labeling task, and autoregressively generates the label sequence of the query protein. Beyond these predictors, a recent comprehensive analysis of motif prediction methods has demonstrated that protein language models, when used together with evolutionary information and physicochemical descriptors, provide powerful and versatile encodings for a wide range of structural and functional motifs, from transmembrane segments to sorting posttranslational modification sites [27].

Although numerous methods have been proposed for signal peptide prediction, the task still faces 2 major challenges: (a) imbalanced data distribution across species in the training datasets, resulting in poor performance on underrepresented species, and (b) the difficulty in accurately identifying the CSs of signal peptides. Moreover, most existing approaches rely primarily on sequence features and do not explicitly model the structural context. Although signal peptide regions are often associated with lower structural order than downstream mature regions, the usefulness of structural information for this task is likely to be context dependent, making it important to design an effective fusion strategy rather than assuming uniformly positive gains from structure alone.

To address these challenges, we propose Signal-3L 4.0, a deep learning framework that integrates a strong pretrained sequence prior with task-specific sequence and structure modeling for signal peptide prediction. Specifically, Signal-3L 4.0 combines frozen ESM2 embeddings with an explicit sequence branch and a structural branch, where sequence features are extracted by multikernel one-dimensional convolutional neural networks (1D-CNNs) and a transformer encoder and structural features are encoded from predicted protein structures and modeled by a parallel transformer. The 2 modalities are then fused through a VisualBERT-style co-attention module [28], after which the fused multimodal representations are concatenated with pretrained ESM2 embeddings and decoded by a conditional random field (CRF) for joint signal peptide classification and CS prediction. To alleviate the impact of long-tail distribution across organism groups and signal peptide types, we further introduce a class-balanced label-distribution-aware margin loss (CB-LDAM loss) [29]. Benchmark and independent-test results show that Signal-3L 4.0 achieves competitive overall performance and improved robustness in several low-resource or challenging settings. Importantly, our analyses indicate that structural information acts as a complementary rather than a primary cue, and its contribution depends on the organism group and signal peptide type.

## Results

### Overview of Signal-3L 4.0

**Fig. 1.**
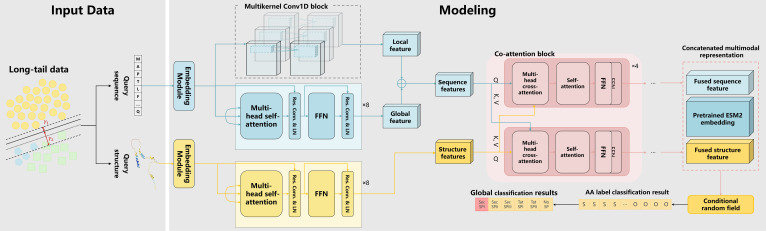
The pipeline of the Signal-3L 4.0. A 2-stream encoder is fused by VisualBERT-style co-attention for residue-level alignment: a sequence stream processed by multikernel one-dimensional convolutional neural networks (1D-CNNs) and a transformer, and a structure stream derived from predicted protein structures and processed by a parallel transformer. The fused multimodal features are concatenated with ESM2 embeddings and decoded by a conditional random field (CRF) to jointly output the signal peptide class and cleavage site. Training uses CRF complemented by class-balanced label-distribution-aware margin loss (CB-LDAM loss) to address the long-tail distribution across organism groups and signal peptide types.

Signal-3L 4.0 is designed to integrate pretrained sequence priors with task-specific sequence and structural modeling for signal peptide prediction (Fig. 1). The sequence stream extracts complementary local and long-range patterns through a multikernel 1D-CNN and a transformer encoder, while the structure stream encodes predicted structural signals and models them with a parallel transformer. A VisualBERT-style co-attention module then enables bidirectional interaction between the 2 modalities to achieve residue-level alignment, after which the fused multimodal features are concatenated with pretrained ESM2 embeddings to incorporate deep semantic and evolutionary priors. A linear projection maps the integrated representation to residue-wise label scores, and a CRF performs sequence-level inference by learning transition dependencies between adjacent states, thereby reducing spurious label switching and improving CS boundary modeling. Training optimizes the CRF negative log-likelihood (NLL), while Viterbi decoding yields the final label sequence from which both the signal peptide class and the CS are derived. To mitigate the long-tail distribution across organism groups and signal peptide types, the objective further incorporates a CB-LDAM loss that complements the CRF training signal.

### Comparison of structural representations

We compared different structural representations used in the Signal-3L 4.0 framework to determine an appropriate default structural input for this task. The compared variants include the following: directly using per-residue predicted local distance difference test (pLDDT) values as structural input (pLDDT feature), masking low-confidence Foldseek tokens according to a pLDDT threshold (pLDDT filter), modulating Foldseek embeddings with a soft pLDDT-based gate (pLDDT gating), using the original Foldseek 20-state geometric tokens (Foldseek), using global continuous structural embeddings derived from FoldExplorer (FoldExplorer), and the structure-removed unimodal baseline obtained from the previous setting (ESM2 + seq + CRF). The results are summarized in Table 1.

**Table 1.** Average performance comparison of structural-representation variants in multimodal Signal-3L 4.0. Metrics include average signal peptide classification performance measured by Avg. SP MCC1 and MCC2, and exact-match cleavage-site F1-scores under a ±0 tolerance window for Sec/SPI, Sec/SPII, and Tat/SPI. The structure-removed baseline corresponds to the ESM2 + sequence-branch + CRF model without structural input. Bold values indicate the best performance for each metric.

Methods	Representation type	Parameter (M)	Avg. SP		Avg. CS F1 (±0)		
			MCC1	MCC2	Sec/SPI	Sec/SPII	Tat/SPI
pLDDT feature	Per-residue pLDDT feature	25.23	0.866	0.838	0.660	0.921	0.587
pLDDT filter	pLDDT-masked Foldseek repr.	25.21	0.882	0.855	0.680	0.923	0.611
pLDDT gating	pLDDT-guided Foldseek repr.	25.21	0.880	0.852	0.674	0.917	**0.621**
Foldseek	Foldseek 20-state geometric repr.	25.21	0.884	0.861	0.688	**0.927**	0.615
FoldExplorer	Graph-enhanced structural repr.	25.34	**0.891**	**0.865**	**0.689**	0.924	0.603
w/o CoAttn & Struc-branch	No structural input	10.38	0.832	0.799	0.634	0.887	0.543

SP, secreted signal peptide; CRF, conditional random field; CS, cleavage site; M, million parameters; pLDDT, predicted local distance difference test; CoAttn, co-attention; Struc-branch, structural branch

Overall, the structural modality provides consistent gains over the structure-removed baseline. Compared with the unimodal Signal-3L 4.0 model without structural input, both the Foldseek-based and FoldExplorer-based multimodal variants achieve higher average SP classification performance and CS prediction performance. For example, introducing the Foldseek representation improves Avg. SP MCC1 and MCC2 by 0.052 and 0.062, respectively. It also improves the exact-match CS F1-scores for Sec/SPI, Sec/SPII, and Tat/SPI by 0.054, 0.040, and 0.072, respectively. These results indicate that structural information is not the primary information source, but it serves as a useful complementary modality that enhances the overall representation capacity of the model.

A further comparison among different structural representations shows that pLDDT-related strategies are helpful but insufficient to replace local geometric structural information. Directly replacing Foldseek with per-residue pLDDT values decreases Avg. SP MCC1/MCC2 by 0.018/0.023 and lowers the CS F1-scores for Sec/SPI, Sec/SPII, and Tat/SPI by 0.028, 0.006, and 0.028, respectively. This suggests that structural confidence contains useful information related to disorder tendency, but its discriminative capacity remains weaker than the local geometric patterns encoded by Foldseek.

Among the pLDDT-based enhancement strategies, pLDDT filtering performs closest to the full Foldseek model, suggesting that pLDDT is more suitable as a reliability cue for screening structural features than as a standalone structural representation. Although pLDDT gating improves over direct pLDDT replacement, it remains slightly weaker than filtering. This may be because filtering explicitly removes low-confidence Foldseek tokens by replacing them with padding tokens, whereas gating only rescales their embeddings, allowing residual noisy structural signals to enter the downstream fusion module. Moreover, since Foldseek is a discrete structural-token representation, continuously scaling its embeddings may not always have a clear structural interpretation. Therefore, pLDDT filtering provides a simpler and more robust confidence-aware strategy in this task.

FoldExplorer, as a continuous global structural embedding, achieves the best average classification performance, with Avg. SP MCC1/MCC2 of 0.891/0.865, slightly higher than those of the Foldseek-based model with 0.884/0.861. However, its advantage is not consistent for CS prediction. For example, FoldExplorer slightly outperforms Foldseek for Sec/SPI CS prediction (0.689 *vs.* 0.688) but performs slightly lower than Foldseek for Sec/SPII and Tat/SPI (0.924 *vs.* 0.927; 0.603 *vs.* 0.615). This indicates that different structural representations contribute differently to different task objectives: continuous global structural embeddings are more beneficial for overall SP classification, whereas local geometric tokens remain advantageous for some CS prediction tasks.

Taken together, these results suggest that structural information mainly plays a complementary role in signal peptide prediction. Foldseek and FoldExplorer represent 2 different structural-representation paradigms: Foldseek emphasizes residue-level local geometric patterns, whereas FoldExplorer provides a more global structural summary at the sequence level. Therefore, in the subsequent benchmark evaluation, we retain both the Foldseek-based and FoldExplorer-based complete variants of Signal-3L 4.0 and systematically compare them with SignalP 6.0.

### Benchmark performance

To compare the performance of Signal-3L 4.0 with the widely recognized predictor SignalP 6.0, we evaluate the signal peptide classification performance (MCC1 and MCC2) and CS prediction performance (precision, recall, and F1 under tolerance windows of ±0 to ±3 residues) across 6 cross-validation model sets. We report 2 variants of Signal-3L 4.0 using different structural representations, namely, the default Foldseek-based version and the FoldExplorer-based version, and compare both against the literature-reported results of SignalP 6.0. In addition to the standard coupled CS metric, we further report unconditional and conditional CS prediction metrics under the strict ±0 criterion to better separate prediction performance from the influence of SP-type classification.

**Fig. 2.**
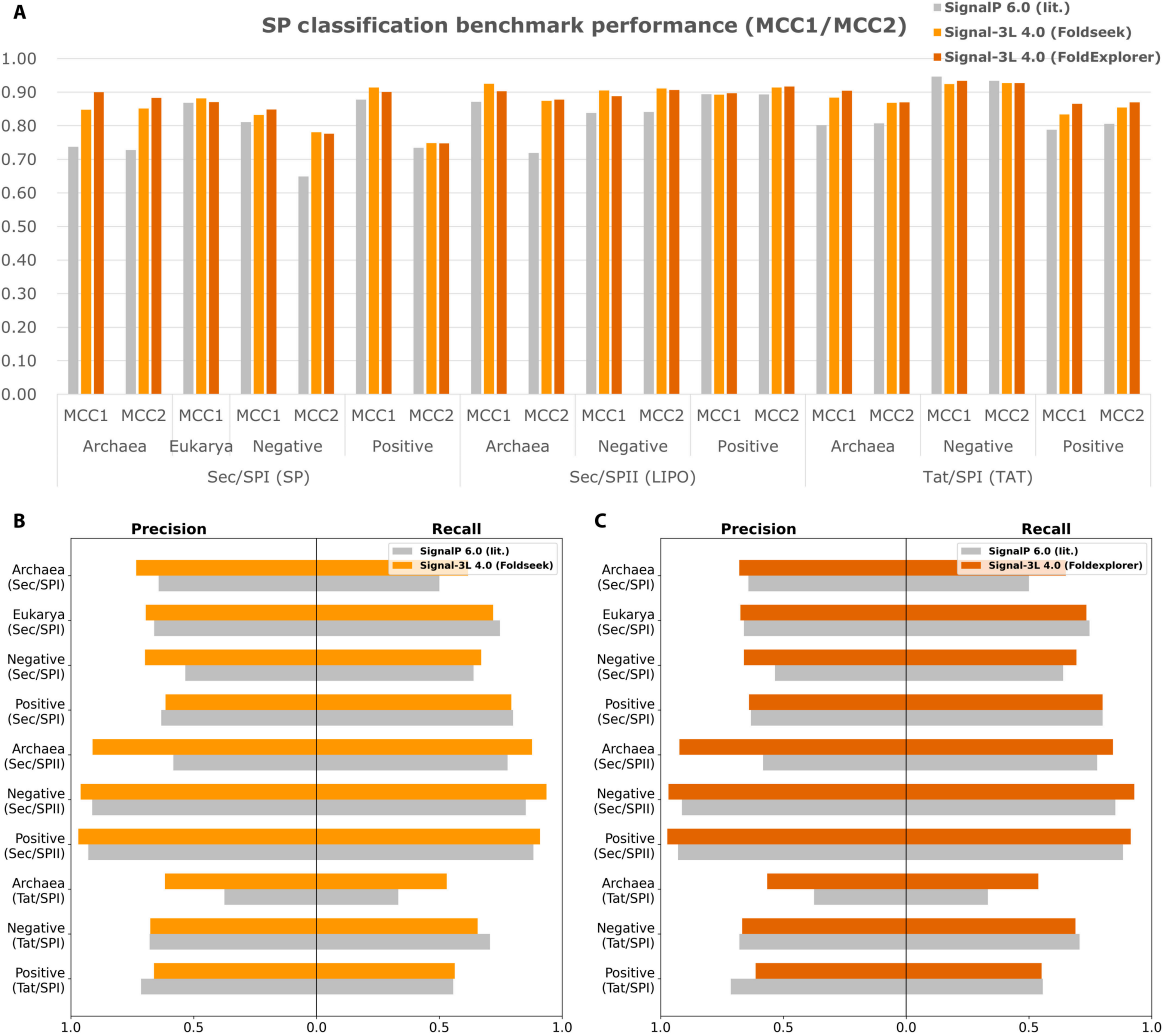
Benchmark performance comparison of Signal-3L 4.0 and SignalP 6.0 on signal peptide classification and cleavage-site prediction. (A) Signal peptide classification performance on the benchmark dataset, comparing SignalP 6.0, Signal-3L 4.0 (Foldseek), and Signal-3L 4.0 (FoldExplorer) across the 3 major secreted signal peptide (SP) types (Sec/SPI, Sec/SPII, and Tat/SPI), evaluated by MCC1 and MCC2. (B and C) Benchmark comparison of cleavage-site prediction performance between SignalP 6.0 and the 2 Signal-3L 4.0 variants, shown as precision and recall at a strict tolerance window of ±0 across organism × SP-type categories, where (B) corresponds to the Foldseek-based model and (C) corresponds to the FoldExplorer-based model.

Signal peptide classification performance: As shown in Fig. 2A and Table S4, both variants of Signal-3L 4.0 outperform SignalP 6.0 in most low-resource or challenging settings while remaining competitive in the remaining categories. For Sec/SPI, clear gains are observed in Archaea and gram-negative bacteria, with smaller improvements in gram-positive bacteria and near-parity in Eukarya. For Sec/SPII, both variants show consistent improvements in Archaea, gram-negative bacteria, and gram-positive bacteria. For Tat/SPI, improvements are most evident in Archaea and gram-positive bacteria, whereas performance in gram-negative bacteria is comparable to that for SignalP 6.0. Overall, the FoldExplorer variant achieves the best macro-average classification performance, with Avg. MCC1/MCC2 of 0.891/0.865, slightly above those of the Foldseek variant (0.884/0.861), and both clearly exceed those for SignalP 6.0 (0.843/0.798) (Table 2). These results indicate that both versions of Signal-3L 4.0 provide stronger sequence-level discrimination than SignalP 6.0 on the benchmark setting, with the FoldExplorer-based structural representation giving a modest additional gain in overall classification.

**Table 2.** Average benchmark performance of Signal-3L 4.0 variants and SignalP 6.0. For each organism × SP-type category, we report (a) MCC1 and MCC2 for signal peptide classification and (b) exact-match cleavage-site F1-scores under the strict ±0 residue tolerance window for Sec/SPI, Sec/SPII, and Tat/SPI. The reported values are macro-averaged across all organism × SP-type conditions. Two Signal-3L 4.0 variants are shown, using Foldseek-based and FoldExplorer-based structural representations, respectively. SignalP 6.0 results are obtained from their original study. Bold values indicate the best performance for each metric.

Model	Avg. SP		Avg. CS F1 (±0)		
	MCC1	MCC2	Sec/SPI	Sec/SPII	Tat/SPI
Signal-3L 4.0 (Foldseek)	0.884	0.861	0.688	**0.927**	**0.615**
Signal-3L 4.0 (FoldExplorer)	**0.891**	**0.865**	**0.689**	0.924	0.603
SignalP 6.0	0.843	0.798	0.638	0.818	0.557

SP, secreted signal peptide; CS, cleavage site

CS prediction performance: We compared CS prediction using the standard coupled evaluation and the unconditional and conditional prediction metrics. Under the coupled evaluation, both Signal-3L 4.0 variants outperform SignalP 6.0 in macro-average exact-match CS F1 for Sec/SPI and Sec/SPII while also improving Tat/SPI overall (Table 2). Specifically, the FoldExplorer variant gives the best macro-average Sec/SPI exact-match CS F1 (0.689), whereas the Foldseek variant performs best for Sec/SPII and Tat/SPI (0.927, 0.615). At the category level as shown in Tables S5 to S7, both variants show particularly strong gains for Archaea across the 3 major SP types, and they also improve Sec/SPII CS prediction in gram-negative and gram-positive bacteria, consistent with the classification trend shown in Fig. 2B and C. For Eukaryotic Sec/SPI, classification is broadly similar across methods, but both Signal-3L 4.0 variants maintain competitive or improved CS prediction. By contrast, several Tat/SPI and gram-positive Sec/SPI settings remain more challenging, and the relative advantage of the 2 structural variants is not uniform across all categories. Detailed coupled CS results, including precision, recall, and F1 across all evaluated tolerance windows, are provided in Tables S5 to S7.

To further decouple prediction from global SP-type recognition, Table 3 summarizes unconditional and conditional exact-match CS F1 (±0) for the 2 Signal-3L 4.0 variants. For unconditional prediction, the FoldExplorer variant yields the best macro-average performance for Sec/SPI (0.730), whereas the Foldseek variant performs best for Sec/SPII and Tat/SPI (0.945 and 0.640). For conditional prediction, the Foldseek variant achieves the highest average exact-match CS F1 for Sec/SPI and Tat/SPI (0.824 and 0.686), while the FoldExplorer variant is marginally higher for Sec/SPII (0.991). These results indicate that the 2 structural representations exhibit slightly different strengths: FoldExplorer provides a small advantage in overall classification and in some Sec/SPI prediction settings, whereas Foldseek remains slightly stronger in several exact-match CS metrics, particularly for Sec/SPII and Tat/SPI.

**Table 3.** Average benchmark cleavage-site prediction performance of Signal-3L 4.0 variants under unconditional and conditional evaluation. We report macro-averaged exact-match CS F1-scores (±0) for Sec/SPI, Sec/SPII, and Tat/SPI. In the unconditional setting, prediction is evaluated without requiring the global SP-type prediction to be correct. In the conditional setting, prediction is evaluated only on proteins whose SP type is correctly predicted. Two Signal-3L 4.0 variants using Foldseek-based and FoldExplorer-based structural representations are compared. Bold values indicate the best performance for each metric.

Model	Avg. unconditional CS F1 (±0)			Avg. conditional CS F1 (±0)		
	Sec/SPI	Sec/SPII	Tat/SPI	Sec/SPI	Sec/SPII	Tat/SPI
Signal-3L 4.0 (Foldseek)	0.727	**0.945**	**0.640**	**0.824**	0.990	**0.686**
Signal-3L 4.0 (FoldExplorer)	**0.730**	0.941	0.635	0.821	**0.991**	0.635

CS, cleavage site; SP, secreted signal peptide

We note that the corresponding conditional and unconditional CS prediction metrics for SignalP 6.0 are not reported here. The available SignalP 6.0 program evaluates the ensemble of the 6 nested cross-validation models as a single full model, whereas our benchmark setting reports the average performance obtained from 6 individual cross-validation model sets. Because these 2 evaluation settings are not directly aligned, we did not compute conditional and unconditional CS prediction metrics for SignalP 6.0 to avoid an unfair comparison. The detailed unconditional and conditional prediction results for the 2 Signal-3L 4.0 variants are provided in Tables S8 to S10.

Taken together, the benchmark results show that both Foldseek-based and FoldExplorer-based Signal-3L 4.0 variants consistently improve over SignalP 6.0 at the classification level and under the standard coupled CS evaluation, while the additional conditional and unconditional analyses further reveal the prediction behavior of the 2 Signal-3L 4.0 variants after decoupling CS prediction from SP-type recognition. The FoldExplorer variant offers the best overall classification performance, whereas the Foldseek variant remains highly competitive and yields the strongest macro-average exact-match CS F1 for Sec/SPII and Tat/SPI, supporting its use as the default structural configuration in the final framework.

### The performance of Signal-3L 4.0 on the independent test set

To further evaluate model robustness on temporally independent and previously unseen data, we assessed an independent test set using the publicly released full-train checkpoints of recent methods, when available. We reproduced mainstream predictors and ran them on this dataset to obtain predictions. PEFT-SP was excluded because only 6 cross-validation checkpoints are available and no full-train model is provided. Because the independent test set contains only positive examples, the GOSP2024 evaluation is conditioned on SP presence and is intended to assess SP-type classification and CS prediction rather than full SP-versus-non-SP detection performance. Accordingly, the reported accuracy is not directly comparable to Matthews correlation coefficient (MCC)-based benchmark results. Signal peptide classification accuracy is defined as the proportion of correctly predicted sequences out of the total sequences, while CS prediction accuracy is defined as the proportion of sequences with correct CS predictions under different tolerance windows.

**Fig. 3.**
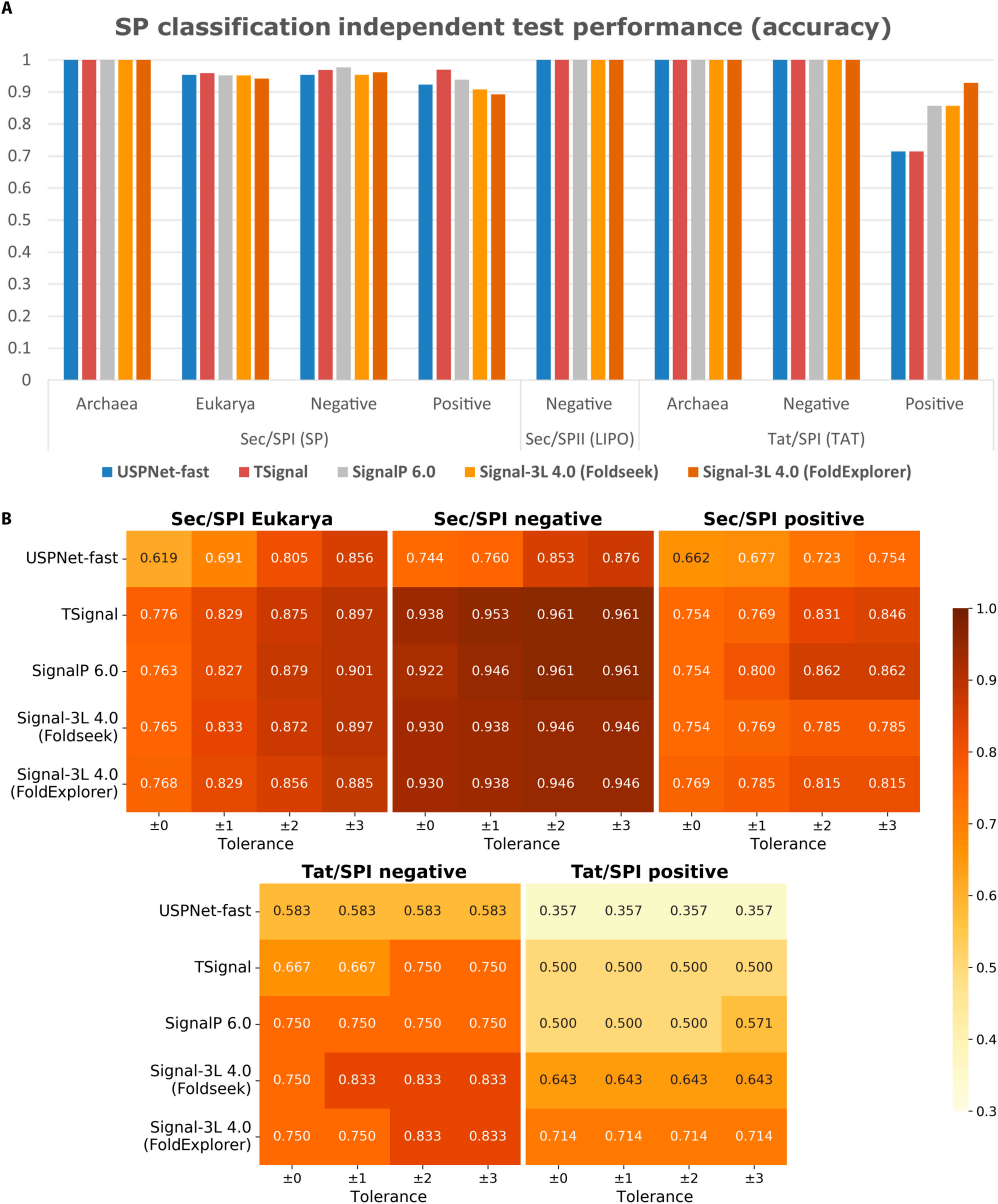
Comparative performance of Signal-3L 4.0 variants and baseline methods on the independent test set. (A) Independent-test comparison of signal peptide classification accuracy, conditional on secreted signal peptide (SP) presence. The panel compares USPNet-fast, TSignal, SignalP 6.0, and 2 variants of Signal-3L 4.0 across the major organism × SP-type combinations in GOSP2024. (B) Cleavage-site prediction performance heatmap for the 5 major organism × SP-type combinations with sufficient sample sizes in the independent test set, including Sec/SPI in Eukarya, gram-negative bacteria, and gram-positive bacteria and Tat/SPI in gram-negative and gram-positive bacteria. Values denote cleavage-site prediction accuracy under tolerance windows of ±0, ±1, ±2, and ±3.

Signal peptide classification performance (Fig. 3A): On the independent test set, the 2 Signal-3L 4.0 variants remain competitive across most organism × SP-type combinations, although their relative strengths are not uniform across categories. For Sec/SPI, both variants perform comparably to the baselines in Eukarya and gram-negative bacteria but remain weaker in gram-positive bacteria. For Tat/SPI, the FoldExplorer variant achieves the highest accuracy in gram-positive bacteria, clearly outperforming SignalP 6.0, whereas the Foldseek variant remains competitive with SignalP 6.0. These results suggest that the independent-test classification performance is stable overall while also indicating category-dependent differences between the 2 structural variants.

CS prediction performance (Fig. 3B and Table 4): Because several organism × SP-type combinations contain extremely small sample sizes, namely, Sec/SPI in Archaea, Sec/SPII in gram-negative bacteria, and Tat/SPI in Archaea, these low-sample categories are presented separately in Table 4 as per-protein prediction counts rather than proportions. Across these 3 rare categories (6 proteins in total), TSignal, SignalP 6.0, Signal-3L 4.0 (Foldseek), and Signal-3L 4.0 (FoldExplorer) each correctly predicted 5 of 6 CSs.

**Table 4.** Cleavage-site prediction results for low-sample categories in the independent test set. Predicted cleavage-site positions are reported for 3 rare organism × SP-type combinations with extremely small sample sizes, namely, Sec/SPI in Archaea, Sec/SPII in gram-negative bacteria, and Tat/SPI in Archaea. For each protein, the table lists the annotated cleavage-site position and the corresponding predictions from USPNet-fast, TSignal, SignalP 6.0, Signal-3L 4.0 (Foldseek), and Signal-3L 4.0 (FoldExplorer). Full per-sequence results for all 740 proteins are provided in Table S22. Bold values indicate predictions that exactly match the annotated cleavage-site positions.

SP type-organism group	Sec/SPI-Archaea		Sec/SPII-Negative		Tat/SPI-Archaea	
Accession	Q980C7	Q50833	P15363	Q8RMH6	D4GPK6	O29751
Ground truth	35	23	23	22	31	26
USPNet-fast	30	18	21	**22**	**31**	**26**
TSignal	30	**23**	**23**	**22**	**31**	**26**
SignalP 6.0	30	**23**	**23**	**22**	**31**	**26**
Signal-3L 4.0 (Foldseek)	30	**23**	**23**	**22**	**31**	**26**
Signal-3L 4.0 (FoldExplorer)	30	**23**	**23**	**22**	**31**	**26**

SP, secreted signal peptide

For the remaining 5 categories with larger sample sizes, the results are summarized in Fig. 3B. For Sec/SPI in Eukarya, both Signal-3L 4.0 variants are competitive, but neither consistently surpasses SignalP 6.0 or TSignal across all tolerance windows. For Sec/SPI in gram-negative bacteria, both variants remain slightly below SignalP 6.0 and TSignal, consistent with the benchmark trend. For Sec/SPI in gram-positive bacteria, the FoldExplorer variant improves over the Foldseek variant under the strict tolerance window (±0 and ±1), but both variants remain below SignalP 6.0 at the more relaxed windows, indicating that gram-positive Sec/SPI CS prediction is still challenging. In contrast, for Tat/SPI, the results are more favorable: in gram-negative bacteria, the Signal-3L 4.0 variants match or exceed SignalP 6.0 at all tolerance windows, especially for the Foldseek variant at ±1 to ±3; in gram-positive bacteria, both variants outperform all baselines, with the FoldExplorer variant achieving the best CS accuracy across all tolerance windows.

Taken together, the independent-test results further support the robustness of Signal-3L 4.0 on previously unseen data, particularly for Tat/SPI in gram-positive bacteria, but they also reveal distinct strengths of the 2 structural variants. The Signal-3L 4.0 variant with FoldExplorer shows a clearer advantage in independent-test classification and in Tat/SPI CS prediction for gram-positive bacteria, whereas the Signal-3L 4.0 variant with Foldseek remains highly competitive in several other settings. Notably, these results should be interpreted carefully for categories with extremely limited sample sizes, especially Archaea in GOSP2024, where only 4 proteins are available in total. In addition, because this independent evaluation is conditioned on SP presence, the reported accuracy is not directly comparable to MCC-based benchmark results. Therefore, the independent-test results further support the robustness of Signal-3L 4.0 across unseen data distributions, although conclusions for extremely low-sample categories should be interpreted with caution.

### Ablation study on Signal-3L 4.0

#### Baseline selection for pretrained language models

To evaluate the impact of different protein language models on the signal peptide prediction task, we selected 3 representative backbones, namely, ESMGearNet, SaProt, and ESM2 (650 MB variant), as described in Note S1. For a fair comparison, each was combined with a CRF layer consistent with SignalP 6.0, resulting in 3 baseline variants: ESMGearNet + CRF, SaProt + CRF, and ESM2 + CRF. For all variants, the parameters of the pretrained models were frozen, and only the pretrained embeddings were used as inputs to the CRF as shown in Fig. S1. All 3 baselines were trained under the same setting as SignalP 6.0, using a CRF loss for residue-level sequence labeling together with an NLL loss for sequence-level classification. Training was performed under identical hyperparameter settings.

**Fig. 4.**
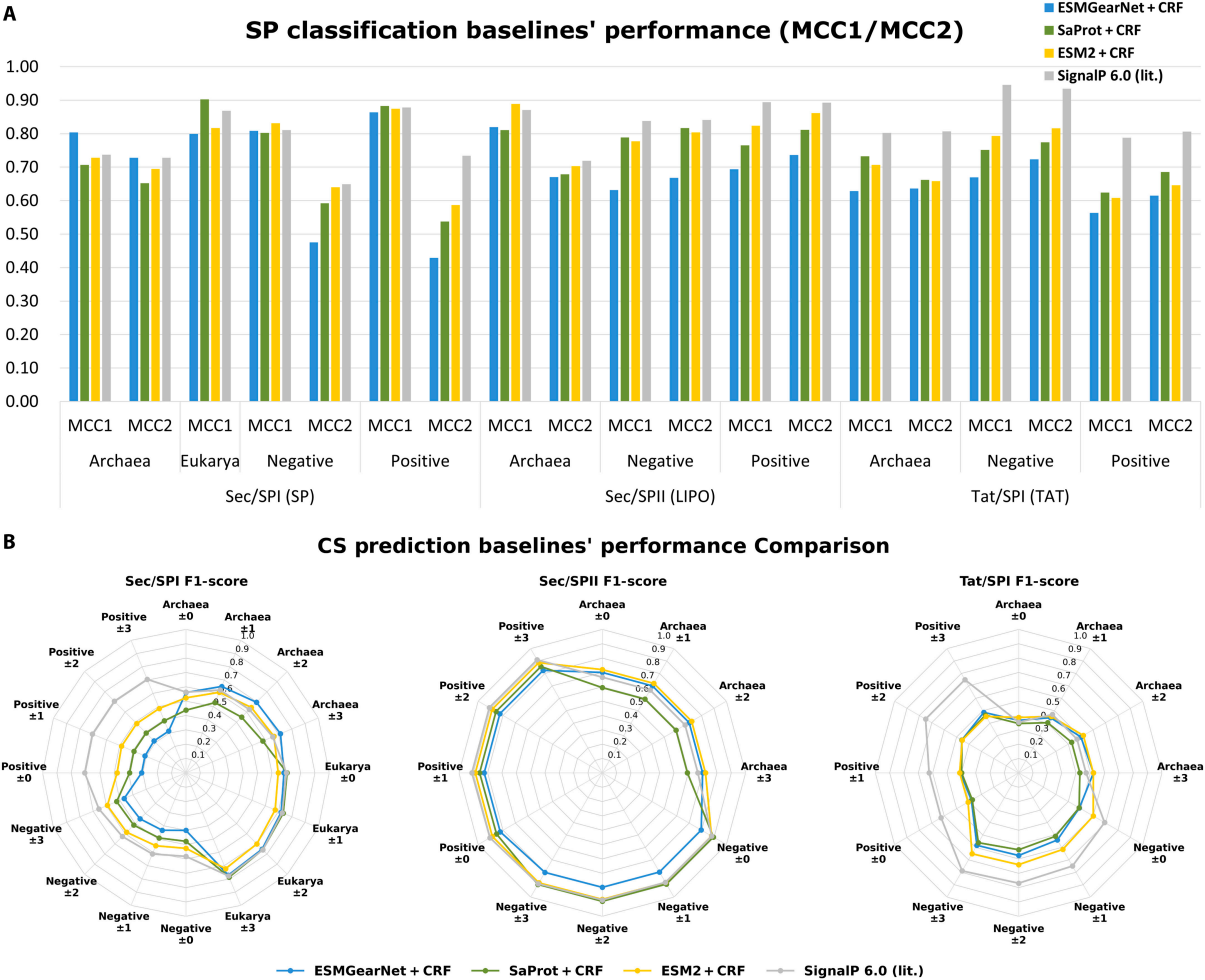
Baseline selection results for Signal-3L 4.0. (A) Performance comparison of different protein language model baselines on the benchmark set. Bar charts show signal peptide classification performance (MCC1/MCC2) for ESMGearNet + conditional random field (CRF), SaProt + CRF, ESM2 + CRF, and SignalP 6.0 across organism × secreted signal peptide (SP)-type combinations. (B) Cleavage-site prediction performance comparison of the same baselines. Radar plots report F1-scores for Sec/SPI, Sec/SPII, and Tat/SPI across organism groups and tolerance windows (±0, ±1, ±2, and ±3).

We evaluated these baselines on the benchmark dataset from SignalP 5.0 and compared them with the literature-reported performance of SignalP 6.0. We reported the performance on both SP classification (MCC1 and MCC2) and CS prediction (precision, recall, and F1-score) across different organisms and SP types. We primarily report MCC for classification and F1-score for CS prediction. The results are summarized in Fig. 4 and Table 5, and the complete per-organism and per-SP-type baseline ablation metrics are provided in Tables S11 to S14.

**Table 5.** Benchmark performance of Signal-3L 4.0 variants and different language model baselines averaged across organism × SP-type categories. Metrics include MCC1/MCC2 for signal peptide classification and cleavage-site F1-scores for Sec/SPI, Sec/SPII, and Tat/SPI. All values are macro-averaged across categories. SignalP 6.0 results are obtained from their original study. Bold values indicate the best performance for each metric.

Baselines	Avg. SP		Avg. CS F1 (±0)		
	MCC1	MCC2	Sec/SPI	Sec/SPII	Tat/SPI
ESMGearNet + CRF	0.746	0.675	0.586	0.806	0.483
SaProt + CRF	0.780	0.727	0.597	0.826	0.482
ESM2 + CRF	0.826	0.778	0.663	**0.867**	0.616
SignalP 6.0	**0.843**	**0.798**	**0.699**	0.823	**0.669**

SP, secreted signal peptide; CS, cleavage site; CRF, conditional random field

In SP classification task, the 3 baselines show a clear overall ranking: ESM2 + CRF consistently outperforms ESMGearNet + CRF and SaProt + CRF across most organism × SP-type combinations, and its performance is more stable and balanced across different species and signal peptide types. Although both ESMGearNet and SaProt introduce structure-aware features during pretraining, ESM2 + CRF still achieves the strongest overall classification performance, especially under the stricter MCC2 evaluation. The same trend is observed for CS prediction: ESM2 + CRF yields the best overall F1 performance among the 3 baselines across Sec/SPI, Sec/SPII, and Tat/SPI. These results indicate that ESM2 provides the most robust and transferable pretrained representation in our setting, rather than excelling only in a limited subset of categories. Given that our downstream tasks require reliable performance across both different organism groups and different SP types, we adopt ESM2 + CRF as the default backbone for Signal-3L 4.0.

Table 5 summarizes the averaged performance across tasks. ESM2 + CRF achieves the highest average SP MCCs (0.826 and 0.778) and the best average CS F1 (0.663/0.867/0.616), surpassing SaProt + CRF (0.597/0.826/0.482) and ESMGearNet + CRF (0.586/0.806/0.483). Compared to SignalP 6.0, ESM2 + CRF is slightly lower on average SP MCC (−0.017/−0.020), Sec/SPI CS F1 (−0.036), and Tat/SPI CS F1 (−0.053) and higher on average Sec/SPII CS F1 (+0.044). These trends, consistent with the per-category analyses in Fig. 3A and B, indicate that ESM2 + CRF offers the better baseline for low-resource regimes; we therefore use it as the default backbone of Signal-3L 4.0 in subsequent experiments.

#### Pipeline ablation

To systematically evaluate the contributions of different components in Signal-3L 4.0, we ablate 4 axes of the full model while keeping training settings fixed: (a) pretrained sequence prior (remove ESM2 embeddings), (b) fusion (remove co-attention), (c) modality (drop the sequence or structural branch), and (d) optimization for imbalance (remove class-balanced loss or replace CB-LDAM loss with NLL loss). Results are shown in Table 6. From the ablation studies on different Signal-3L 4.0 variants, we obtain 4 observations as follows:

• Pretrained knowledge is indispensable. Removing ESM2 (“w/o ESM2 emb”) leads to a noticeable performance drop across several metrics. Specifically, removing ESM2 decreases Avg. SP MCC1/MCC2 by 0.059/0.078, from 0.884/0.861 to 0.825/0.783, and decreases CS F1 by 0.144, 0.025, and 0.109 for Sec/SPI, Sec/SPII, and Tat/SPI, respectively. While removing the sequence branch (“w/o CoAttn & Seq-branch”) also causes substantial drops, particularly for MCC and Sec/SPI CS F1, the removal of ESM2 produces a more pervasive impact across all categories, particularly in Sec/SPI and Tat/SPI CS prediction, where the losses are greater than those observed when the sequence branch is removed.
• Cross-modal fusion is consistently helpful. Without co-attention, Avg. SP MCC1/MCC2 decreases by 0.038/0.053, from 0.884/0.861 to 0.846/0.808. The average CS F1 also drops across all 3 SP types, with decreases of 0.067 for Sec/SPI, 0.042 for Sec/SPII, and 0.059 for Tat/SPI. These results indicate that co-attention is more effective than simple feature concatenation, as it enables the 2 modalities to interact and improves both classification and CS prediction.
• Sequence is primary; structure is complementary. Dropping the sequence branch (“w/o CoAttn & Seq-branch”) decreases more (MCC −0.068/−0.089; CS F1: Sec/SPI −0.087, Sec/SPII −0.069, and Tat/SPI −0.072) than dropping structure (“w/o CoAttn & Struc-branch”: MCC −0.052/−0.062; CS F1: Sec/SPI −0.054, Sec/SPII −0.040, and Tat/SPI −0.072), indicating that the sequence branch remains the dominant source of predictive information. Nevertheless, the structural branch provides additional complementary gains, especially for Sec/SPI and Sec/SPII, as shown by the higher performance of the full model over both single-branch ablations.
• Class-imbalance-aware objectives are beneficial overall, but their effects are category dependent. Removing class-balanced weighting or replacing class-balanced label-distribution-aware margin (CB-LDAM loss) with NLL loss leads to small but consistent reductions in average classification performance relative to the full model. Specifically, “w/o CB-weighting” gives 0.884/0.853 (MCC1/MCC2) and “Replace CB-LDAM loss with NLL loss” gives 0.882/0.857, both of which are slightly below or roughly comparable to the full model in average MCC and CS F1. These results indicate that imbalance-aware learning contributes to the overall robustness of the model, although the magnitude of the gain differs across SP types. These findings suggest that class-imbalance-aware training improves the model’s ability to handle minority classes, but its effect varies depending on the SP type and the specific dataset.

**Table 6.** Settings and performance of ablation experiments. Metrics include MCC1/MCC2 for signal peptide classification and cleavage-site F1-scores (tolerance ±0) for Sec/SPI, Sec/SPII, and Tat/SPI. All values are macro-averaged across categories. FLOPs are reported as GFLOPs. Bold values indicate the best performance for each metric.

Methods	Parameters (M)	GFLOPs	Avg. SP		Avg. CS F1 (±0)		
			MCC1	MCC2	Sec/SPI	Sec/SPII	Tat/SPI
Full model (with Foldseek emb)	25.2	3.66	0.884	0.861	0.688	**0.927**	0.615
Full model (with FoldExplorer emb)	25.3	3.68	**0.891**	**0.865**	**0.689**	0.924	0.603
w/o CoAttn	16.8	2.40	0.846	0.808	0.621	0.885	0.556
w/o CoAttn & Struc-branch	10.4	1.48	0.832	0.799	0.634	0.887	0.543
w/o ESM2 emb	25.2	3.65	0.825	0.783	0.544	0.902	0.506
w/o CoAttn & Seq-branch	6.4	0.93	0.816	0.774	0.601	0.858	0.543
w/o CB-weighting	25.2	3.66	0.884	0.853	0.685	0.914	0.611
Replace CB-LDAM loss with NLL loss	25.2	3.66	0.882	0.857	0.685	0.921	0.608

SP, secreted signal peptide; CS, cleavage site; M, million parameters; FLOPs, floating-point operations; GFLOPs, giga floating-point operations; emb, embedding(s); CoAttn, co-attention; Struc-branch, structural branch; Seq-branch, sequence branch; CB-weighting, class-balanced weighting; CB-LDAM loss, class-balanced label-distribution-aware margin loss; NLL loss, negative log-likelihood loss

The studies show that pretrained sequence knowledge (ESM2) and the sequence branch are foundational—removing ESM2 produces the comparable decreases, and dropping sequence hurts more than dropping structure. Co-attention consistently improves both classification and CS prediction and is superior to naive concatenation, as it aligns modalities to sharpen boundaries. The structural branch adds complementary information, but the advantage of replacing Foldseek with FoldExplorer is limited and not uniform across all tasks. Imbalance-aware training with CB-LDAM loss is beneficial overall, although its effects remain category dependent. Overall, the best performance arises from the combination of pretrained knowledge, co-attentive multimodal fusion, and calibrated imbalance-aware objectives.

### Interpretability analysis

**Fig. 5.**
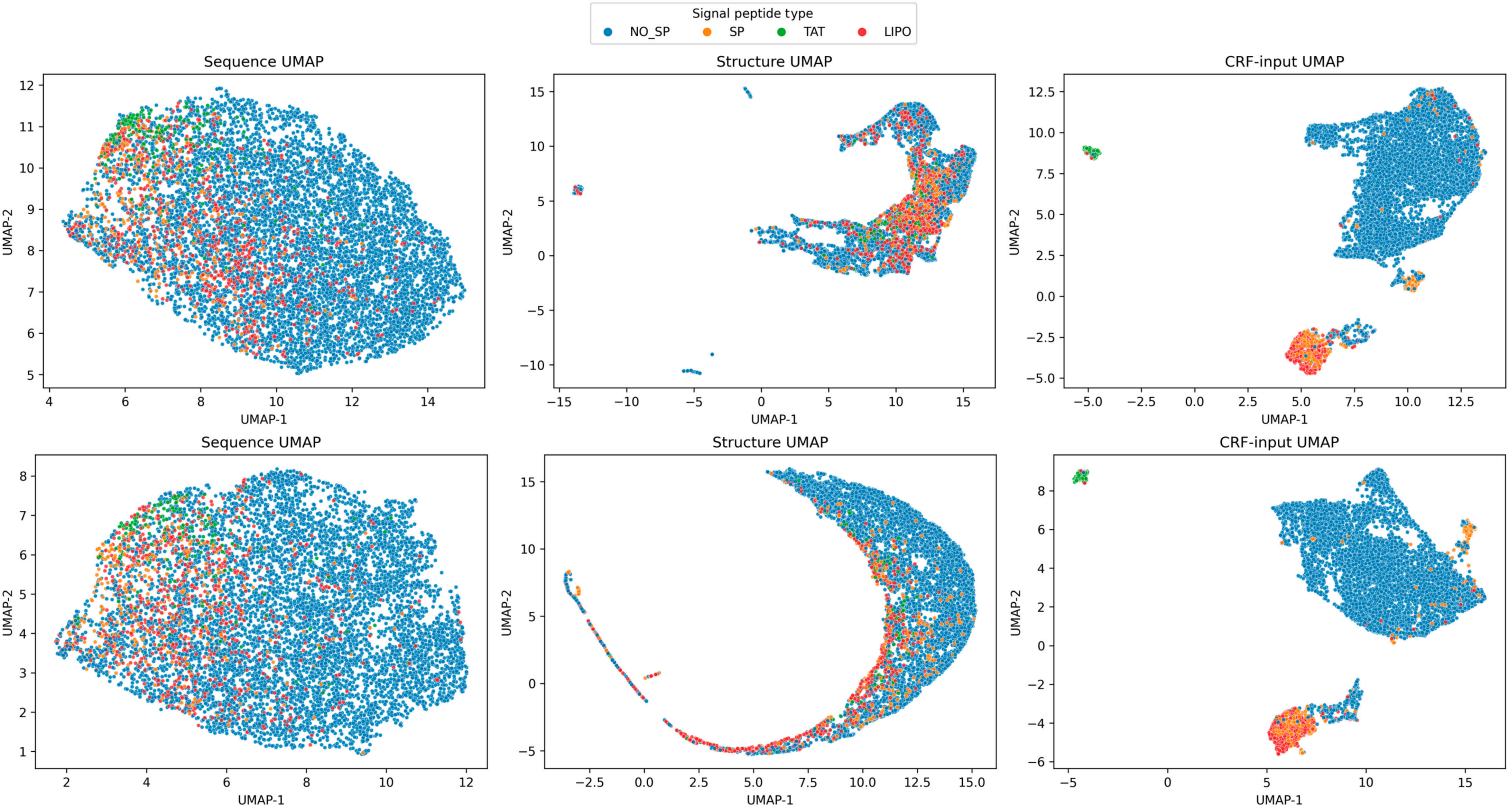
Uniform manifold approximation and projection (UMAP) visualization of intermediate and fused embeddings in the 2 full-model variants of Signal-3L 4.0. The top row shows the Foldseek-based variant, including the sequence-branch embedding, structure-branch embedding, and conditional random field (CRF)-input embedding from left to right. The bottom row shows the FoldExplorer-based variant in the same order. Colors indicate signal peptide types and the NO_SP class.

To better visualize the representation characteristics of the 2 full-model variants, we illustrate the distributions of sequence-branch embeddings, structure-branch embeddings, and CRF-input embeddings in the uniform manifold approximation and projection (UMAP)-reduced space. The top row in Fig. 5 corresponds to the Foldseek-based full model, whereas the bottom row corresponds to the FoldExplorer-based full model.

As shown in Fig. 5, the sequence-branch embeddings of the Foldseek and FoldExplorer variants are highly similar. This is expected because the 2 variants share the same sequence-processing pipeline and differ only in their structural representations. In both cases, the sequence branch already shows partial enrichment of SP-containing proteins in some local regions, but substantial overlap with the dominant NO_SP class remains, indicating that the sequence branch alone provides only limited class separation at this intermediate stage.

The structure-branch embeddings exhibit more distinct differences between the 2 variants. In the Foldseek-based model, the structure embedding space appears more fragmented and locally organized, suggesting that Foldseek captures residue-level structural patterns in a relatively discrete manner. In contrast, the FoldExplorer-based structure embeddings form a smoother and more continuous manifold, consistent with a more global structural representation. These results indicate that the 2 structural encoders organize protein structure information in different ways, even when used within the same downstream framework.

The clearest separation emerges in the CRF-input embedding space after multimodal fusion. In both variants, the fused embeddings show a markedly stronger partition between the NO_SP-dominated major region and the signal-peptide-containing regions than is observed in either single branch alone, indicating that multimodal fusion substantially improves the organization of the representation space before sequence decoding. At the same time, the minority TAT class forms a region that is separated farther from the major NO_SP cluster than the intermediate-frequency SP and LIPO classes. Although this observation is qualitative, it is consistent with the design motivation of CB-LDAM loss, which encourages larger decision margins for underrepresented classes and may therefore contribute to improved minority-class separability in the fused space.

Taken together, these visualizations suggest that the sequence branch provides a stable representational basis across the 2 variants, whereas Foldseek and FoldExplorer introduce structurally distinct embedding geometries. More importantly, the CRF-input embeddings indicate that the final fused representation is substantially more organized than either individual branch, especially for the coarse separation between NO_SP and signal-peptide-containing proteins.

## Discussion

In this study, we proposed Signal-3L 4.0, a deep learning framework designed to address key challenges in signal peptide prediction, particularly the long-tail distribution in the training data, in which some categories contain only a small number of examples and are therefore difficult to model accurately. By incorporating both sequence and structural features, Signal-3L 4.0 improves prediction performance, particularly for certain rare or complex signal peptide types.

Signal-3L 4.0 integrates sequence and structural features through a dual-path encoder architecture followed by a VisualBERT-style co-attention module for cross-modal fusion. The ESM2 embeddings provide strong pretrained sequence representations, while the additional sequence branch captures task-specific local sequence patterns. The structural branch introduces complementary geometric cues, which are further integrated with sequence-derived features through co-attention. In addition, CB-LDAM loss helps mitigate class imbalance, especially for underrepresented categories, although its effect can be category and metric dependent.

Through extensive benchmarking on the SignalP dataset, we demonstrate that Signal-3L 4.0 achieves strong performance in both overall classification and CS prediction. The model shows particularly clear improvements in Archaea and Sec/SPII, highlighting its effectiveness in low-resource settings as reflected by the benchmark results. The additional conditional and unconditional CS analyses further indicate that CS performance should be interpreted together with SP-type classification accuracy. The unconditional metric reflects end-to-end utility, whereas the conditional metric helps isolate localization ability once the SP type has been correctly identified. It also shows competitive performance on previously unseen data in the independent test set, particularly for Tat/SPI in gram-negative and gram-positive bacteria. Although some categories in GOSP2024 remain limited in sample size, especially Archaea, the independent-test results still provide additional support for the generalization ability of Signal-3L 4.0.

Nevertheless, several limitations remain, particularly for challenging categories such as gram-positive Sec/SPI and some Tat/SPI settings. Although the current 70-residue input window is well aligned with the N-terminal nature of signal peptide recognition and usually covers the major local structural transition around the CS neighborhood, it may still provide limited mature-region structural context for proteins with unusually long signal peptides. More broadly, the structural encoding strategy still requires further optimization. Our comparison between pLDDT-based variants, Foldseek, and FoldExplorer indicates that different structural representations capture different aspects of protein structure: pLDDT mainly reflects confidence or disorder tendency, Foldseek captures local geometric patterns, and FoldExplorer provides a more global structural summary. Designing structural encoders that better combine local geometric resolution with global structural context remains an important direction for future work. In addition, integrating high-dimensional ESM2 embeddings with lower-dimensional structural features remains a nontrivial challenge. Future work may therefore benefit from exploring richer structural representations, more effective multimodal alignment strategies, extended input lengths, and broader datasets to further improve the robustness and generalization ability of Signal-3L 4.0.

## Methods

### Data collection and preprocessing

This study used a training set, a benchmark test set, and an independent test set, together with their corresponding predicted protein structures.

#### Training set

The training dataset used in this work was adopted from SignalP 6.0. It contains the organism groups—Archaea, Eukarya, gram-positive, and gram-negative—as well as the signal peptide and non-signal-peptide categories defined in SignalP 6.0. In addition, it incorporates newly added SP sequences from UniProt [30] and Prosite [31], as well as soluble and transmembrane proteins from UniProt and Topology Data Bank of Transmembrane Proteins (TOPDB) [32]. Homologous sequence clustering and redundancy reduction were performed based on the method proposed by Gíslason *et al.* [33], whereby protein sequences with high sequence similarity were removed from the dataset.

The original training set contained 20,290 protein sequences. During data reorganization, we found that 7 sequence entries had been updated. Details of these updates are provided in Table S19. The final training dataset comprises 20,289 sequences, including 15,622 negative samples (i.e., soluble or transmembrane proteins that do not contain signal peptides) and 4,667 positive samples. The positive samples include 3,352 Sec/SPI sequences, 2,261 Sec/SPII sequences, 113 Sec/SPIII sequences, 595 Tat/SPI sequences, and 36 Tat/SPII sequences. In this 6-class classification task, the Sec/SPIII, Tat/SPI, and Tat/SPII types are underrepresented, resulting in a long-tail label distribution.

To ensure a fair comparison with previous results, we followed the original data partitioning strategy in SignalP 6.0 and directly adopted its 3-way split of the training data. These subsets were preprocessed to ensure low sequence similarity across different partitions while maintaining a balanced representation of organism groups (Archaea, Eukarya, gram-positive bacteria, and gram-negative bacteria) within each subset. Additionally, similar sequences were grouped within the same partition to avoid homologous overlapping between subsets. Each protein sequence in the dataset is annotated with its signal peptide type and residue-level labels, which indicate the signal peptide region and CS.

#### Benchmark test set

The benchmark test dataset in this study is the reorganized SignalP 5.0 benchmark test dataset, as used in SignalP 6.0. The original benchmark set contained 8,811 protein sequences across 4 classes: Sec/SPI, Sec/SPII, Tat/SPI, and NO_SP. During dataset preparation, we found that 4 sequences had been updated. The final benchmark set consists of 8,810 sequences, including 7,579 NO_SP, 375 Sec/SPI, 662 Sec/SPII, and 194 Tat/SPI.

#### Independent test set

We constructed a new independent test dataset named GOSP2024 to further evaluate the performance of our model and other signal peptide predictors on previously unseen data. This dataset was derived from the UniProtKB database and contains protein sequences released after December 2020, ensuring that it was compiled later than the SignalP 6.0 dataset. The construction process was as follows:

1. All sequences released before December 2020 were removed.
2. All sequences from virus species were excluded, retaining only proteins from Archaea, Eukarya, gram-positive, and gram-negative groups.
3. Sequences annotated with signal peptides and carrying high-confidence experimental evidence tags (ECO:0000269) were selected.

An initial set of 3,807 signal peptide-containing protein sequences was collected. To reduce redundancy, we used CD-HIT [34] to remove sequences with greater than 40% identity to any sequence in the union of the SignalP 6.0 training and benchmark datasets while also keeping the internal redundancy of GOSP2024 below 80%. The final GOSP2024 dataset contains 740 nonredundant sequences: 4 from Archaea, 514 from Eukarya, 143 from gram-negative bacteria, and 79 from gram-positive bacteria. The details of the dataset are shown in Table S20.

#### Structure dataset

The structural data corresponding to each protein sequence were obtained using a unified pipeline across the training set, benchmark set, and independent test set. Specifically, we first searched AlphaFold DB [35,36] for available predicted structures for each retained protein sequence. If no corresponding AlphaFold structure was available for a given sequence, we used ESMFold [37] to predict its structure. Importantly, sequence-level filtering and de-redundancy were completed before structural feature extraction, and structural representations were retrieved or generated only for the final retained sequence set. As a result, 147 additional protein structures were predicted for the training and benchmark datasets of SignalP 6.0, and 9 additional structures were predicted for the GOSP2024 dataset. The UniProt accession numbers of the corresponding proteins are listed in Table S21.

### Sequence feature extraction module

The sequence feature extraction module accepts protein sequences with lengths up to 70 amino acids. First, each amino acid is converted into a unique numerical index, tokenizing the protein sequence to obtain a vector $X\in {\mathbb{R}}^{70\times 1}$, where L≤70 is the sequence length. Then, the vector passes sequentially through an embedding layer and a linear mapping layer, projecting the feature vector into a higher-dimensional space to yield the base sequence vector $X\in {\mathbb{R}}^{70\times 256}$.

Subsequently, the sequence feature vector is fed into 2 modules for further feature extraction: the local feature extraction module and the global feature extraction module. To better capture multiscale contextual dependencies within local regions, the local feature extraction module employs 3 parallel 2-layer 1D convolutional branches with kernel sizes 3, 5, and 7, respectively. Smaller kernels are more sensitive to local patterns, such as short-range interactions within a signal peptide subregion, while larger kernels capture broader context to recognize local structural or functional signals spanning across subregions. Through the parallel combination of multiscale convolutions, the model can extract rich local sequence features at different granularities, improving its ability to identify signal peptide boundaries and key residues. The outputs of the 3 convolutional branches corresponding to different kernel sizes are summed element-wise to fuse local features under varying receptive fields, enhancing the response signals at amino acid positions highly relevant to the signal peptide region.

Hlocal=Conv1D3X+Conv1D5X+Conv1D7X (1)

where $X\in {\mathbb{R}}^{70\times 256}$ denotes the input sequence representation with a length 70; $\text{Conv}1D^{k}\left(\cdot \right)$ denotes a 1D convolutional operation with kernel size k; $\text{Conv}1D^{3}\left(X\right)$, $\text{Conv}1D^{5}\left(X\right)$, and $\text{Conv}1D^{7}\left(X\right)$ extract local contextual features under receptive fields of different scales; and the element-wise summation integrates these multiscale convolutional outputs to produce a unified local representation that captures both short-range and slightly longer-range sequence patterns within the signal peptide region.

Since biologically functional key residues often exhibit strong signals across multiple contextual scales, this summation allows highly correlated positions to receive amplified feature scores, while positions weakly related to the task tend to have relatively flat responses at all scales, maintaining low overall features after summation. This fusion strategy helps magnify representation differences in critical regions, thereby improving the model’s discriminative ability for signal peptide recognition tasks.

To further explore long-range dependencies among residues in protein sequences, we introduce an encoding module composed of 8 stacked transformer encoder layers, each containing an 8-head self-attention mechanism. This module can model interactions between any 2 residues in parallel, overcoming the limitations of traditional convolutional or recurrent networks in capturing long-range dependencies. The transformer’s self-attention dynamically computes attention weights between every pair of positions based on learned Query, Key, and Value vectors derived from the input vector, updating each position’s representation according to the global context. By stacking multiple layers and employing multihead mechanisms, the model can capture semantic relations at different hierarchical levels, learning deep representations with global information. The input to the lth transformer encoder layer is $X^{\left(l\right)}\in {\mathbb{R}}^{70\times 256}$, and the computation proceeds as

AttentionQKV=softmaxQKTdkV (2)

headi=AttentionXWiQXWiKXWiV (3)

MultiHeadX=Concathead1…headhWO∈ℝ70×256 (4)

X1=LayerNormX+MultiHeadX (5)

X=LayerNormX1+FFNX1 (6)

where $1/\sqrt{d_{k}}$ is a scaling factor to prevent excessively large dot-product values between Query and Key vectors that could push the softmax toward one-hot vectors. $d_{k}$ is the dimension of each attention head’s Query/Key vectors, specifically 256/8=32. Tdenotes matrix transpose. $W_{i}^{Q},W_{i}^{K},W_{i}^{V}\in {\mathbb{R}}^{256\times 32}$, and $W^{O}\in {\mathbb{R}}^{256\times 256}$ are learnable linear projection matrices.

Finally, the outputs from the 2 modules, the local feature vector $H_{\mathrm{seq}-\text{local}}\in {\mathbb{R}}^{70\times 256}$ and the global feature vector $H_{\mathrm{seq}-\text{global}}\in {\mathbb{R}}^{70\times 256}$, are summed element-wise to obtain the enhanced high-dimensional sequence feature vector $H_{\mathrm{seq}}\in {\mathbb{R}}^{70\times 256}$.

### Structure feature extraction module

In the structure feature processing module, we considered several alternative types of structural representations, namely, pLDDT feature, pLDDT filtering, pLDDT gating, Foldseek-based structural tokens [38], and FoldExplorer-based protein embeddings [39]. For both settings, the corresponding protein structures were first obtained from the structure dataset described in the “Structure dataset” section, and only the N-terminal region within the first 70 amino acid residues was used for subsequent modeling.

We explored 3 pLDDT-based confidence-aware strategies. The motivation is that signal peptide regions often show low AlphaFold confidence, and low-pLDDT positions may contain stronger disorder or higher structural noise as shown in Fig. S4 and Tables S1 to S3. Let $p=[p_{1},\;p_{2},\;\ldots ,\;p_{70}]$, $p_{t}\in \left[0,100\right]$, denote the per-residue pLDDT scores within the N-terminal 70-residue window. The first strategy, pLDDT feature, directly uses pLDDT as structural input by projecting each scalar score into a 256-dimensional vector: $Y_{t}={\mathrm{MLP}}_{\text{pLDDT}}\left(p_{t}\right)$, $Y\in {\mathbb{R}}^{70\times 256}$.

The second strategy, pLDDT filter, retains Foldseek tokens but masks low-confidence positions. Specifically, when $p_{\tau }<\tau$ with τ=50, the corresponding Foldseek token is replaced by the padding token:

st′=st,pt≥τPAD,pt<τ (7)

The filtered token sequence is then embedded as Y=${\mathrm{Emb}}_{\text{Foldseek}}\left(s^{\prime }\right)$. This strategy treats pLDDT as a reliability filter for removing low-confidence structural signals.

The third strategy, pLDDT gating, uses pLDDT to softly modulate Foldseek embeddings. Given the Foldseek embedding $E_{t}$, the gate is computed as

pt^=pt100 (8)

gt=0.2+0.8·σWgpt^+bg (9)

Yt=gt·Et (10)

Here, $g_{t}\in \left[0.2,1.0\right]$, so low-confidence structural features are weakened but not completely removed. These 3 variants respectively test whether pLDDT is more suitable as a standalone confidence feature, a hard reliability filter, or a soft modulation signal for Foldseek-based geometric representations.

For the Foldseek-based representation, each protein structure in Protein Data Bank (PDB) format was encoded by Foldseek, which discretizes local geometric information through a structural alphabet defined by spatial relationships between residues and their neighboring residues. We extracted the 20-state discrete structural tokens generated by Foldseek, which were aligned to residue positions. These tokens were directly converted into 256-dimensional trainable vector representations through an embedding layer, yielding a residue-level structural representation $Y\in {\mathbb{R}}^{70\times 256}$.

For the FoldExplorer-based representation, each protein structure was encoded into a continuous 512-dimensional embedding by jointly modeling structural topology and sequence information using a graph attention network and a protein language model. This global structural embedding was first projected into a 256-dimensional latent space through a linear layer. Since the downstream multimodal fusion operates at the residue level, the projected FoldExplorer embedding was then expanded along the sequence dimension and replicated across the first 70 residue positions, resulting in a residue-aligned structural feature matrix $Y\in {\mathbb{R}}^{70\times 256}$.

To further model contextual relationships in the structural features, we applied an 8-layer transformer encoder to the resulting residue-level structural representation, obtaining the high-dimensional structural feature vectors $H_{\text{struc}}\in {\mathbb{R}}^{70\times 256}$. This design keeps the dimensionality and downstream processing of the 2 structural representations consistent, thereby facilitating subsequent multimodal fusion and comparative evaluation under the same framework.

### Feature fusion module

To fully integrate the protein sequence and structure features, we draw inspiration from the co-attention fusion mechanism proposed in VisualBERT [28] and construct a 4-layer multimodal co-attention network. This module performs symmetric information exchange between the input sequence and structure representations, progressively capturing fine-grained correlations between the 2 modalities. In each co-attention layer, the following operations are performed sequentially:

1. Cross-attention: For each modality (e.g., sequence modality), it acts as the Query, while the other modality (e.g., structure modality) acts as Key and Value to compute attention responses. In each layer of cross-attention, the attention interaction between the sequence vectors $H_{\mathrm{seq}}$ and the structure vectors $H_{\text{struc}}$ is implemented as follows:

H~seql=MultiHeadQseqlKstruclVstruclH~strucl=MultiHeadQstruclKseqlVseql (11)

Here, l denotes the layer index, and ${\tilde{H}}_{\mathrm{seq}}^{\left(l\right)},{\tilde{H}}_{\text{struc}}^{\left(l\right)}\in {\mathbb{R}}^{70\times 256}$ represent the fused sequence and structure features output by the lth co-attention layer.

2. Self-attention: Each modality then separately executes a multihead self-attention mechanism to capture internal long-range dependencies.
3. Feedforward neural network (FFN): Each modality passes through a 2-layer feedforward network to enhance the expressiveness.
4. Residual connection and layer normalization: After each of the above operations, residual connections and layer normalization are applied to improve training stability and model performance.

After passing through the 4-layer co-attention encoder, both modalities are fused and mutually enriched in contextual information, producing the fused sequence vector ${\tilde{H}}_{\mathrm{seq}}\in {\mathbb{R}}^{70\times 256}$ and the fused structure vector ${\tilde{H}}_{\text{struc}}\in {\mathbb{R}}^{70\times 256}$.

To further enrich feature representations, ${\tilde{H}}_{\mathrm{seq}}$, ${\tilde{H}}_{\text{struc}}$, and the sequence embedding $H_{\mathrm{ESM}2}\in {\mathbb{R}}^{70\times 1,280}$ produced by the ESM2-650M model are concatenated, resulting in the final fused feature vector $H_{\text{fused}}\in {\mathbb{R}}^{70\times 1,792}$. This design integrates the co-attention-enhanced sequence and structural features with the pretrained sequence representation before CRF decoding.

### CRF for label decoding

The fused feature vector $H_{\text{fused}}$ from the feature fusion module is passed through a linear projection layer to obtain the CRF-input representation $H_{\mathrm{CRF}-\text{input}}\in {\mathbb{R}}^{70\times 37}$, where 37 is the number of residue-level labels used in this study. The projected features are then decoded by a linear-chain CRF, which models dependencies between adjacent labels for sequence labeling [40]. Given the input hidden state sequence $h=\left(h_{1},\;h_{2},\ldots ,\;h_{T}\right)$ and the corresponding output label sequence $y=\left(y_{1},\;y_{2},\ldots ,\;y_{T}\right)$, the conditional probability is defined as follows:

$$\begin{array}{l} P\left(y|h\right)=\frac{1}{Z\left(h\right)}\exp\left(\sum_{t=1}^{T}\psi \left(h_{t}\right)+\sum_{t=1}^{T-1}\phi \left(y_{t},y_{t+1}\right)\right)\\ \;\psi \left(h_{t}\right)=W_{\psi }h_{t}+b_{\psi } \end{array}$$ (12)

where $\phi \left(y_{t},\;y_{t+1}\right)$ denotes the transition score between adjacent labels, parameterized by a learnable state transition matrix of size $n_{c}\times n_{c}$, where $n_{c}$ is the number of labels used in the CRF for this task. $\psi \left(h_{t}\right)$ is the emission score obtained by mapping the hidden vector $h_{t}$ to the label space. During training, the CRF is optimized using the NLL of the target label sequence. During inference, the most probable label path is obtained by Viterbi decoding:

y^=argmaxyPy∣h (13)

The decoded residue-level label sequence is then used to determine the signal peptide region, CS boundary, and the corresponding sequence-level signal peptide type.

### Implementation details

#### Loss function and regularization in Signal-3L 4.0

To jointly optimize the accuracy of signal peptide region prediction and address the class imbalance in signal peptide label distribution, we design a composite loss function consisting of CRF loss and label-distribution-aware margin (LDAM) loss [29]. CRF loss optimizes the sequence labeling task, and LDAM loss optimizes the sequence-level signal peptide classification task. The sequence-level SP-type label is derived from the same annotation schema as the residue-level labels. Specifically, the residue labels within the signal peptide region determine the corresponding global SP type, so the 2 supervision signals are naturally aligned rather than independently defined.

We implement the CRF loss as the NLL of a linear-chain CRF, following the residue-level annotation setting adopted in SignalP 6.0. Let $h=\left(h_{1}\;,\;h_{2}\;,\ldots ,h_{T}\right)$ denote the sequence of hidden states, $y=\left(y_{1}\;,\;y_{2}\;,\;\ldots ,\;y_{T}\right)$ denote the corresponding label sequence, and $M_{t}$ denote the set of ground-truth labels assigned to position t. The conditional probability of the label sequence is defined as

−logP(y∣h)=logZh−logexp∑t=1Tψht+∑yt∈MtΦytyt−1 (14)

In the signal peptide classification task, we address the training bias caused by class imbalance by introducing CB-LDAM loss. This loss combines LDAM margin adjustment with class-balanced reweighting to improve minority-class discrimination. The core idea of LDAM loss is to add a class-dependent margin to the standard cross-entropy objective, imposing stronger constraints on majority classes and encouraging the model to allocate wider decision boundaries for minority classes. Specifically, the margin for class j is defined as

Δyj=Cnj1/4 (15)

where $n_{j}$ is the number of samples in class j and C is a tunable hyperparameter that controls the overall margin scale. During training, this margin is subtracted from the logit corresponding to the ground-truth class to suppress overconfident predictions for majority classes. In this task, the ground-truth logit $z_{y}$ is adjusted by the margin Δy before applying the softmax operation:

Lxy=−logezy−Δysezy−Δys+∑j≠yezjs (16)

$z_{y}$ is the logit for the true label y, $z_{j}$ are the logits for all other classes, Δy is the class-specific margin, and s is the softmax scaling factor (typically set to 30) that controls the sharpness of the output distribution.

To further enhance the model’s capability of handling class imbalance, we incorporate class-balanced reweighting. This strategy computes class-wise loss weights based on the notion of “effective number of samples”:

wj=1−β1−βnj (17)

where $\beta \in \left(0,\;1\right)$ is a balancing hyperparameter (typically around 0.99) that controls the degree of reweighting. This weight reflects the relative importance of each class in the overall training loss and effectively mitigates the dominance of majority classes during optimization.

In practice, we first adjust the logit of the true class by its LDAM margin and then compute the cross-entropy loss. This sample-wise loss is then multiplied by the corresponding CB weight, resulting in the final CB-LDAM loss, used to improve minority-class discrimination under the long-tail training distribution:

LCB−LDAMxy=wy·Lxy (18)

The final overall loss function is composed of the CRF loss for CS prediction and the CB-LDAM loss for SP classification:

L=LCRF+LCB−LDAM (19)

In this study, no additional weighting coefficient was introduced between the 2 loss terms; the total loss is defined as a direct 1:1 summation of the residue-level CRF loss and the sequence-level CB-LDAM loss. We also did not apply any explicit gradient-balancing strategy, in order to keep the interaction between the 2 supervision signals.

#### Experiment setting

In the final framework, ESM2-650M was used as the pretrained sequence backbone. During training, the parameters of ESM2-650M were kept frozen, and only the downstream modules were updated.

We adopted a nested cross-validation strategy similar to SignalP 6.0. The training dataset was divided into 3 subsets with minimal sequence similarity between them, ensuring roughly balanced distributions of different organism groups and signal peptide types within each subset while minimizing sequence similarity across subsets. On this basis, a 3-fold outer cross-validation was performed, with 2-fold inner cross-validation in each outer fold for model selection and early stopping. In total, 3×2=6 models were trained for evaluation.

Model training was conducted on a single NVIDIA RTX 3090 graphics processing unit using the Adam optimizer with a learning rate of 0.0001 and a weight decay coefficient of 1e−5. The batch size was set to 512. Additionally, a warm-up strategy was applied during the first 5% of the total training steps to improve training stability. Model selection and early stopping were based on 2 validation-set metrics, namely, detection MCC1 (for signal peptide classification) and CS F1-score (for CS prediction). A checkpoint was retained only when both metrics improved over their previous best values according to the predefined stopping criterion; otherwise, the early-stopping counter was increased.

### Performance evaluation

For the signal peptide classification task, we used MCC to evaluate SP identification performance. Two MCC variants were considered. MCC1 treats the target SP type as the positive class and both soluble proteins and transmembrane proteins as the negative class. MCC2 is a stricter metric in which the target SP type is treated as the positive class, whereas all other SP types and non-SP sequences are treated as the negative class. MCC2 therefore provides a more challenging evaluation of discriminative ability. MCC is calculated as follows:

MCC=TP×TN−FP×FNTP+FP×TP+FN×TN+FP×TN+FN (20)

For the CS prediction task, we used precision, recall, and F1-score to evaluate prediction performance. To account for annotation uncertainty, a tolerance-window mechanism was adopted: a prediction was considered correct if it fell within ±0, ±1, ±2, or ±3 residues of the annotated CS.

We reported 3 types of CS evaluation metrics. Under the coupled evaluation, a CS prediction was counted as correct only when the corresponding SP type was also predicted correctly. Under the unconditional evaluation, CS prediction was assessed without requiring the global SP-type prediction to be correct; for a given SP type, unconditional recall was defined over all true proteins of that type, whereas unconditional precision was defined over all proteins predicted as that type. Under the conditional evaluation, CS prediction was assessed only on the subset of proteins whose SP type was predicted correctly. In this setting, conditional precision and conditional recall are mathematically identical, and therefore, the conditional prediction performance was summarized using a single conditional CS F1/accuracy metric. Detailed definitions and formulas for these 3 evaluation protocols are provided in Note S2.

## Data Availability

Signal-3L 4.0 is freely available for academic use as a web server at http://www.csbio.sjtu.edu.cn/bioinf/Signal-3L/ and as open-source code at https://github.com/chandlevier/Signal-3L-4.0.

## Supplementary Materials

Supplementary 1 Notes S1 and S2 Figs. S1 to S4 Tables S1 to S22
