# Supplementary material for: A Co-attention Mechanism of Jointly Capturing Sequence and Structure Features for Signal Peptide Prediction
Source: Comput Struct Biotechnol J. 2026 Jul 8;35(1):0120. doi: 10.34133/csbj.0120 (PMC13440004; doi:10.34133/csbj.0120)
Supplement: Supplementary file 1 [file csbj.0120.f1.docx]

**A Co-Attention Mechanism of Jointly Capturing Sequence and Structure Features for Signal Peptide Prediction**

**Supplementary Information**

Zhixuan Piao, Yuan Liu, Xiaoyong Pan, and Hong-Bin Shen^*^

**Supplementary note 1: Baseline model**

To retain the strengths of the SignalP 6.0 framework while exploring more expressive pretrained encoders, we designed a unified prediction architecture that replaces ProtBERT [1] with three state-of-the-art pretrained models: ESM2 [2], ESMGearNet [3], and SaProt [4]. This resulted in three model variants—ESM2+CRF, ESMGearNet+CRF, and SaProt+CRF. All models preserve the CRF-based sequence labeling module from SignalP 6.0 to enable fair comparisons of different encoders in signal peptide prediction tasks.

These three encoders were selected for the following reasons: (1) ESM2 features a deeper architecture and is pretrained on a larger corpus, demonstrating superior performance across a range of downstream protein-related tasks, and is thus expected to capture more fine-grained semantic features in sequences; (2) ESMGearNet and SaProt incorporate protein structural information during pretraining, enabling their representations to simultaneously capture both sequence and structure contexts, effectively integrating multi-modal features.

**1 ESM2**

ESM2 is a new generation of large-scale protein language model family developed by Meta AI, designed to capture evolutionary and structural information embedded in protein sequences. It adopts a BERT-style encoder-only Transformer architecture and is pretrained on over 60 million protein sequences sourced from the UniRef50 database (September 2021 release). The ESM2 model series ranges in size from hundreds of millions to 15 billion parameters. In this study, we utilize the ESM2-650M variant.

To enhance generalization, the ESM2 pretraining dataset is rigorously filtered to exclude artificial and de novo designed sequences. Additionally, MMseqs2 is employed to deduplicate sequences between the training and validation sets, ensuring that sequence identity remains below 50%. Unlike traditional absolute positional encoding methods, ESM2 adopts Rotary Position Embedding (RoPE) to enable better extrapolation of positional information beyond the training context window, thereby improving its ability to model long sequences. During training, dropout in both attention and hidden layers is removed to free up capacity.

Compared to earlier models such as ESM-1b and ProtBERT, ESM2 demonstrates significant improvements in representation quality, particularly in downstream tasks like protein structure and function prediction. Its embeddings have been widely applied to protein classification, functional annotation, and structure prediction, and have shown strong expressiveness in capturing fine-grained semantic features in protein sequences.

**2 ESMGearNet**

ESMGearNet is a hybrid representation learning framework that integrates protein language models (PLMs) with structural encoders, combining the deep semantic features learned by ESM2 with the geometric structural features extracted by structural graph neural networks (such as GearNet) to bridge the gap between sequence-only and structure-only modeling paradigms.

For sequence modeling, ESMGearNet employs a pretrained ESM2 model (specifically, the ESM2-650M variant used in this study) to encode protein sequences. For structural modeling, it uses GearNet to construct a multi-relational residue graph incorporating sequential edges, spatial radius edges, and k-nearest neighbor (K-NN) edges, which capture sequence order, distance, and local topology respectively. The model then performs multi-relational message passing to update residue representations and encode structural information effectively. To fuse the two modalities, ESMGearNet introduces three fusion strategies: serial fusion, parallel fusion, and cross fusion. In the experimental evaluations, the authors compared these fusion methods across several downstream tasks, using three structural encoders (GearNet, GVP, and CDConv) in combination with ESM2.

Results show that the serial fusion strategy consistently outperforms the others across most tasks. Combining GearNet with ESM2 yields significant improvements. Although GearNet alone underperforms compared to other structural encoders, its performance improves markedly when integrated with a language model, highlighting the complementary strength of sequence-based representations in structural modeling. Moreover, compared to using ESM2 alone, ESMGearNet demonstrates superior generalization and expressiveness, especially in tasks that involve structural information.

**3 SaProt**

SaProt is a structure-aware protein language model designed to integrate primary amino acid sequences with tertiary structural information to enhance performance on downstream protein prediction tasks. The model introduces a Structure-Aware Vocabulary, in which each amino acid residue is paired with a corresponding structure token—derived using Foldseek [5]—to form a combined token. This results in a vocabulary of 441 tokens representing all possible combinations of residue and structural states.

SaProt adopts the same Transformer encoder architecture as ESM2-650M, with modifications to the input embedding layer to accommodate the structure-aware tokens. It is pretrained using a masked language modeling (MLM) objective on approximately 40 million protein sequences with AlphaFold2-predicted structures, following the same training strategy as ESM2. Although the structural data is computationally predicted rather than experimentally determined, the introduction of structure-aware tokens provides valuable structural priors that enhance the model’s generalization capability.

Evaluation across ten downstream protein tasks shows that SaProt consistently outperforms mainstream PLMs, including ESM2 and ProtBERT, in areas such as function prediction and mutation effect analysis. The learned protein embeddings capture fine-grained sequence semantics while also encoding higher-order structural information, demonstrating strong general-purpose representation learning capabilities applicable to a wide range of bioinformatics tasks.

Based on comparative experimental results, using ESM2-650M as the backbone yields the best performance. Building on this, we enrich the feature representation and feed it, together with the pretrained embeddings from ESM2-650M, into a CRF for decoding.

**Supplementary note 2: Definitions of coupled, unconditional, and conditional CS metrics**

For a given SP type $c$ and tolerance window $w$, let $m_{i}^{(w)}$ denote whether the predicted cleavage site of sample $i$falls within $w$ residues of the annotated cleavage site. Based on this indicator, we define three complementary evaluation protocols for cleavage-site (CS) prediction: coupled, unconditional, and conditional evaluation.

Under the coupled evaluation, a cleavage-site prediction is counted as correct only when the corresponding SP type is also predicted correctly. This protocol jointly evaluates global SP-type recognition and cleavage-site localization, and therefore reflects the end-to-end prediction performance of the model.

$$\begin{aligned} \mathrm{Recall}_{\mathrm{coupled}}^{\left( c,w \right)}=\frac{\sum_{i=1}^{N} \boldsymbol{1}\left( y_{i}= c, \hat{y_{i}}= c \right) m_{i}^{\left( w \right)}}{\sum_{i=1}^{N} \boldsymbol{1}\left( y_{i}= c \right)}\#\left( 1 \right) \end{aligned}$$

$$\begin{aligned} \mathrm{Precision}_{\mathrm{coupled}}^{\left( c,w \right)}=\frac{\sum_{i=1}^{N} \boldsymbol{1}\left( y_{i}= c, \hat{y_{i}}= c \right) m_{i}^{\left( w \right)}}{\sum_{i=1}^{N} \boldsymbol{1}\left( \hat{y_{i}}= c \right)}\#\left( 2 \right) \end{aligned}$$

$$\begin{aligned} {F1}_{\mathrm{coupled}}^{\left( c,w \right)}=\frac{2\cdot\mathrm{Precision}_{\mathrm{coupled}}^{\left( c,w \right)}\cdot\mathrm{Recall}_{\mathrm{coupled}}^{\left( c,w \right)}}{\mathrm{Precision}_{\mathrm{coupled}}^{\left( c,w \right)}+\mathrm{Recall}_{\mathrm{coupled}}^{\left( c,w \right)}}\#\left( 3 \right) \end{aligned}$$

Under the unconditional evaluation, cleavage-site localization is assessed without requiring correct SP-type prediction. For a given SP type, unconditional recall is defined over all true proteins of that type, whereas unconditional precision is defined over all proteins predicted as that type. This protocol reduces the influence of global SP-type classification and more directly reflects cleavage-site localization ability.

$$\begin{aligned} \mathrm{Recall}_{\mathrm{uncond}}^{\left( c,w \right)}=\frac{\sum_{i=1}^{N} \boldsymbol{1}\left( y_{i}= c \right) m_{i}^{\left( w \right)}}{\sum_{i=1}^{N} \boldsymbol{1}\left( y_{i}= c \right)}\#\left( 4 \right) \end{aligned}$$

$$\begin{aligned} \mathrm{Precision}_{\mathrm{uncond}}^{\left( c,w \right)}=\frac{\sum_{i=1}^{N} \boldsymbol{1}\left( \hat{y_{i}}= c \right) m_{i}^{\left( w \right)}}{\sum_{i=1}^{N} \boldsymbol{1}\left( \hat{y_{i}}= c \right)}\#\left( 5 \right) \end{aligned}$$

$$\begin{aligned} {F1}_{\mathrm{uncond}}^{\left( c,w \right)}=\frac{2\cdot\mathrm{Precision}_{\mathrm{uncond}}^{\left( c,w \right)}\cdot\mathrm{Recall}_{\mathrm{uncond}}^{\left( c,w \right)}}{\mathrm{Precision}_{\mathrm{uncond}}^{\left( c,w \right)}+\mathrm{Recall}_{\mathrm{uncond}}^{\left( c,w \right)}}\#\left( 6 \right) \end{aligned}$$

Under the conditional evaluation, cleavage-site localization is assessed only on the subset of samples whose SP type is predicted correctly, described as $\mathcal{S}_{c}= \left\{ i\mid y_{i}= c,\hat{y_{i}}= c \right\}$. In this setting, because both the numerator and denominator are restricted to the same subset of correctly classified proteins, conditional precision and conditional recall become mathematically identical. Therefore, the conditional localization performance is summarized using a single conditional CS F1/accuracy metric.

$$\begin{aligned} {F1}_{\mathrm{cond}}^{\left( c,w \right)}=\mathrm{Accuracy}_{\mathrm{co}nd}^{\left( c,w \right)}=\frac{\sum_{i=1}^{N} \boldsymbol{1}\left( y_{i}= c, \hat{y_{i}}= c \right) m_{i}^{\left( w \right)}}{\sum_{i=1}^{N} \boldsymbol{1}\left( y_{i}= c, \hat{y_{i}}= c \right)}\#\left( 7 \right) \end{aligned}$$

Together, these three evaluation protocols provide complementary perspectives on cleavage-site prediction: the coupled metric reflects end-to-end practical performance, the unconditional metric evaluates localization with reduced dependence on classification correctness, and the conditional metric measures localization quality after correct SP-type recognition.

**Supplementary references**

1. Elnaggar A, Heinzinger M, Dallago C, et al. ProtTrans: Toward Understanding the Language of Life Through Self-Supervised Learning. IEEE Transactions on Pattern Analysis and Machine Intelligence 2022; 44:7112–7127

2. Lin Z, Akin H, Rao R, et al. Evolutionary-scale prediction of atomic-level protein structure with a language model. Science 2023; 379:1123–1130

3. Zhang Z, Wang C, Xu M, et al. ESM-GearNet: A Systematic Study of Joint Representation Learning on Protein Sequences and Structures. 2023;

4. Su J, Han C, Zhou Y, et al. SaProt: Protein Language Modeling with Structure-aware Vocabulary. 2023;

5. van Kempen M, Kim SS, Tumescheit C, et al. Fast and accurate protein structure search with Foldseek. Nat Biotechnol 2023; 42:243–246

**Figure S1.** Baseline architectures for evaluating different protein language models on signal peptide prediction. Three SOTA pretrained encoders (ESM2-650M, ESMGearNet and SaProt) are frozen and used to generate residue-level embeddings, which are fed into a CRF tagging layer identical to that of SignalP 6.0, yielding three variants (ESM2-

650M+CRF, ESMGearNet+CRF and SaProt+CRF) trained under identical hyperparameter settings.

**Figure S4.** Cleavage-site-centered AlphaFold2 pLDDT profiles for Sec/SPII & Tat/SPI proteins across organism groups. Top: Sec/SPII. Bottom: Tat/SPI. Dashed line indicates the cleavage site (CS).

**Table S1.** Quantitative summary of AlphaFold2 pLDDT statistics for Sec/SPI proteins across organism groups. The table reports the sample size (n), along with the mean, median, and standard deviation (Std) of pLDDT scores within the signal peptide region (relative positions < 0), and the mean pLDDT score within a cleavage-site-centered local window ([-5, +5]).

| **Organism** | **Sample**  **Size (n)** | **SP Region pLDDT Scores** | | | | | **Local Window [-5, +5] Mean** |
| --- | --- | --- | --- | --- | --- | --- | --- |
|  |  | **Mean** | **Median** | | **Std** | |  |
| Archaea | 44 | 41.29 | | 37.28 | | 15.04 | 54.05 |
| Eukarya | 2041 | 57.72 | | 55.42 | | 16.96 | 64.79 |
| Negative | 356 | 48.6 | | 46.93 | | 14.14 | 64.6 |
| Positive | 142 | 44.76 | | 41.35 | | 13.69 | 59.5 |

**Table S2.** Quantitative summary of AlphaFold2 pLDDT statistics for Sec/SPII proteins across organism groups.

| **Organism** | **Sample**  **Size (n)** | **SP Region pLDDT Scores** | | | | | **Local Window [-5, +5] Mean** |
| --- | --- | --- | --- | --- | --- | --- | --- |
|  |  | **Mean** | **Median** | | **Std** | |  |
| Archaea | 12 | 44.87 | | 42.34 | | 10.11 | 45.73 |
| Negative | 1088 | 57.38 | | 56.34 | | 15.91 | 58.4 |
| Positive | 516 | 49.95 | | 47.65 | | 13.9 | 53.65 |

**Table S3.** Quantitative summary of AlphaFold2 pLDDT statistics for Tat/SPI proteins across organism groups.

| **Organism** | **Sample**  **Size (n)** | **SP Region pLDDT Scores** | | | | | **Local Window [-5, +5] Mean** |
| --- | --- | --- | --- | --- | --- | --- | --- |
|  |  | **Mean** | **Median** | | **Std** | |  |
| Archaea | 13 | 33.65 | | 31.24 | | 9.49 | 47.08 |
| Negative | 313 | 57.96 | | 56.48 | | 16.72 | 56.91 |
| Positive | 39 | 47.22 | | 43.84 | | 15.3 | 62.41 |

**Table S4.** Signal-peptide classification performance of Signal-3L 4.0 variants and SignalP 6.0 on the benchmark dataset. MCC1 and MCC2 are reported for each organism × SP-type category. Two Signal-3L 4.0 variants are shown, using Foldseek-based and FoldExplorer-based structural representations, respectively. Bold indicates the best performance among the compared methods.

| **Sec/SPI (SP)** | | | | | | | | | | | |
| --- | --- | --- | --- | --- | --- | --- | --- | --- | --- | --- | --- |
| Methods | ARCHAEA | | EUKARYA | NEGATIVE | | | POSITIVE | |  |  |  |
|  | MCC1 | MCC2 | MCC1 | MCC1 | MCC2 | | MCC1 | MCC2 |  |  |  |
| Signal-3L 4.0 (Foldseek) | 0.848 | 0.852 | **0.881** | 0.832 | **0.781** | | **0.914** | **0.749** |  |  |  |
| Signal-3L 4.0 (FoldExplorer) | **0.900** | **0.883** | 0.871 | **0.848** | 0.777 | | 0.901 | 0.748 |  |  |  |
| SignalP 6.0 (Lit.) | 0.737 | 0.728 | 0.868 | 0.811 | 0.649 | | 0.878 | 0.734 |  |  |  |
| **Sec/SPII (LIPO)** | | | | | | | | | | | |
| Methods | ARCHAEA | | - | NEGATIVE | | | POSITIVE | | | |  |
|  | MCC1 | MCC2 | - | MCC1 | MCC2 | | MCC1 | MCC2 |  |  |  |
| Signal-3L 4.0 (Foldseek) | **0.925** | 0.874 | - | **0.905** | **0.911** | | 0.892 | 0.914 |  |  |  |
| Signal-3L 4.0 (FoldExplorer) | 0.903 | **0.878** | - | 0.888 | 0.906 | | **0.897** | **0.917** |  |  |  |
| SignalP 6.0 (Lit.) | 0.871 | 0.719 | - | 0.838 | 0.841 | | 0.894 | 0.893 |  |  |  |
| **Tat/SPI (TAT)** | | | | | | | | | | | |
| Methods | ARCHAEA | | - | NEGATIVE | | POSITIVE | | | |  |  |
|  | MCC1 | MCC2 | - | MCC1 | MCC2 | | MCC1 | MCC2 | | | |
| Signal-3L 4.0 (Foldseek) | 0.884 | 0.868 | - | 0.924 | 0.927 | | 0.833 | 0.854 | | | |
| Signal-3L 4.0 (FoldExplorer) | **0.904** | **0.870** | - | 0.934 | 0.927 | | **0.865** | **0.870** | | | |
| SignalP 6.0 (Lit.) | 0.802 | 0.807 | - | **0.946** | **0.934** | | 0.788 | 0.806 | | | |

**Table S4.** Signal-peptide classification performance of Signal-3L 4.0 variants and SignalP 6.0 on the benchmark dataset. MCC1 and MCC2 are reported for each organism × SP-type category. Two Signal-3L 4.0 variants are shown, using Foldseek-based and FoldExplorer-based structural representations, respectively. Bold indicates the best performance among the compared methods.

| **Sec/SPI (SP)** | | | | | | | | | | | |
| --- | --- | --- | --- | --- | --- | --- | --- | --- | --- | --- | --- |
| Methods | ARCHAEA | | EUKARYA | NEGATIVE | | | POSITIVE | |  |  |  |
|  | MCC1 | MCC2 | MCC1 | MCC1 | MCC2 | | MCC1 | MCC2 |  |  |  |
| Signal-3L 4.0 (Foldseek) | 0.848 | 0.852 | **0.881** | 0.832 | **0.781** | | **0.914** | **0.749** |  |  |  |
| Signal-3L 4.0 (FoldExplorer) | **0.900** | **0.883** | 0.871 | **0.848** | 0.777 | | 0.901 | 0.748 |  |  |  |
| SignalP 6.0 (Lit.) | 0.737 | 0.728 | 0.868 | 0.811 | 0.649 | | 0.878 | 0.734 |  |  |  |
| **Sec/SPII (LIPO)** | | | | | | | | | | | |
| Methods | ARCHAEA | | - | NEGATIVE | | | POSITIVE | | | |  |
|  | MCC1 | MCC2 | - | MCC1 | MCC2 | | MCC1 | MCC2 |  |  |  |
| Signal-3L 4.0 (Foldseek) | **0.925** | 0.874 | - | **0.905** | **0.911** | | 0.892 | 0.914 |  |  |  |
| Signal-3L 4.0 (FoldExplorer) | 0.903 | **0.878** | - | 0.888 | 0.906 | | **0.897** | **0.917** |  |  |  |
| SignalP 6.0 (Lit.) | 0.871 | 0.719 | - | 0.838 | 0.841 | | 0.894 | 0.893 |  |  |  |
| **Tat/SPI (TAT)** | | | | | | | | | | | |
| Methods | ARCHAEA | | - | NEGATIVE | | POSITIVE | | | |  |  |
|  | MCC1 | MCC2 | - | MCC1 | MCC2 | | MCC1 | MCC2 | | | |
| Signal-3L 4.0 (Foldseek) | 0.884 | 0.868 | - | 0.924 | 0.927 | | 0.833 | 0.854 | | | |
| Signal-3L 4.0 (FoldExplorer) | **0.904** | **0.870** | - | 0.934 | 0.927 | | **0.865** | **0.870** | | | |
| SignalP 6.0 (Lit.) | 0.802 | 0.807 | - | **0.946** | **0.934** | | 0.788 | 0.806 | | | |

**Table S5.** Coupled cleavage-site prediction performance of Signal-3L 4.0 variants and SignalP 6.0 on the benchmark dataset for Sec/SPI signal peptides. Recall, precision, and F1-score are reported under tolerance windows of ±0, ±1, ±2, and ±3 residues for each organism group. Two Signal-3L 4.0 variants are shown, using Foldseek-based and FoldExplorer-based structural representations, respectively. Bold indicates the best performance among the compared methods.

| Methods | ARCHAEA | | | | EUKARYA | | | | NEGATIVE | | | | POSITIVE | | | |
| --- | --- | --- | --- | --- | --- | --- | --- | --- | --- | --- | --- | --- | --- | --- | --- | --- |
|  | ±0 | ±1 | ±2 | ±3 | ±0 | ±1 | ±2 | ±3 | ±0 | ±1 | ±2 | ±3 | ±0 | ±1 | ±2 | ±3 |
| Coupled CS recall | | | | | | | | | | | | | | | | |
| Signal-3L 4.0 (Foldseek) | 0.617 | 0.680 | 0.703 | 0.727 | 0.719 | 0.770 | 0.799 | 0.819 | 0.670 | 0.710 | 0.738 | 0.749 | 0.793 | **0.807** | **0.807** | **0.807** |
| Signal-3L 4.0 (FoldExplorer) | **0.650** | **0.720** | **0.753** | **0.777** | 0.734 | **0.783** | **0.810** | 0.828 | **0.693** | **0.728** | **0.757** | **0.768** | **0.8** | **0.807** | **0.807** | **0.807** |
| SignalP 6.0 (Lit.) | 0.500 | 0.556 | 0.556 | 0.583 | **0.747** | 0.774 | 0.808 | **0.829** | 0.639 | 0.672 | 0.689 | 0.721 | **0.8** | 0.8 | 0.8 | 0.8 |
| Coupled CS precision | | | | | | | | | | | | | | | | |
| Signal-3L 4.0 (Foldseek) | **0.734** | **0.807** | **0.835** | **0.862** | **0.695** | **0.744** | **0.772** | **0.791** | **0.698** | **0.738** | **0.767** | **0.779** | 0.615 | 0.624 | 0.624 | 0.624 |
| Signal-3L 4.0 (FoldExplorer) | 0.680 | 0.752 | 0.788 | 0.812 | 0.675 | 0.720 | 0.746 | 0.762 | 0.661 | 0.693 | 0.719 | 0.730 | **0.640** | **0.644** | **0.644** | **0.644** |
| SignalP 6.0 (Lit.) | 0.643 | 0.714 | 0.714 | 0.750 | 0.661 | 0.685 | 0.715 | 0.733 | 0.534 | 0.562 | 0.575 | 0.603 | 0.632 | 0.622 | 0.632 | 0.632 |
| Coupled CS F1 score | | | | | | | | | | | | | | | | |
| Signal-3L 4.0 (Foldseek) | **0.707** | **0.757** | **0.785** | **0.805** | 0.684 | 0.724 | 0.752 | 0.764 | 0.693 | 0.704 | 0.704 | 0.704 | **0.707** | **0.757** | **0.785** | **0.805** |
| Signal-3L 4.0 (FoldExplorer) | 0.703 | 0.750 | 0.777 | 0.793 | 0.677 | 0.711 | 0.737 | 0.748 | **0.711** | **0.716** | **0.716** | **0.716** | 0.703 | 0.750 | 0.777 | 0.793 |
| SignalP 6.0 (Lit.) | 0.563 | 0.625 | 0.625 | 0.656 | **0.701** | **0.727** | **0.759** | **0.778** | 0.582 | 0.612 | 0.627 | 0.657 | 0.706 | 0.700 | 0.706 | 0.706 |

**Table S6.** Coupled cleavage-site prediction performance of Signal-3L 4.0 variants and SignalP 6.0 on the benchmark dataset for Sec/SPII signal peptides.

| Methods | ARCHAEA | | | | NEGATIVE | | | | POSITIVE | | | |
| --- | --- | --- | --- | --- | --- | --- | --- | --- | --- | --- | --- | --- |
|  | ±0 | ±1 | ±2 | ±3 | ±0 | ±1 | ±2 | ±3 | ±0 | ±1 | ±2 | ±3 |
| Coupled CS recall | | | | | | | | | | | | |
| Signal-3L 4.0 (Foldseek) | **0.877** | **0.877** | **0.877** | **0.877** | **0.936** | **0.937** | **0.937** | **0.940** | 0.910 | 0.910 | 0.910 | 0.910 |
| Signal-3L 4.0 (FoldExplorer) | 0.842 | 0.842 | 0.842 | 0.842 | 0.929 | 0.929 | 0.929 | 0.933 | **0.915** | **0.915** | **0.915** | **0.915** |
| SignalP 6.0 (Lit.) | 0.778 | 0.778 | 0.778 | 0.778 | 0.852 | 0.852 | 0.856 | 0.864 | 0.883 | 0.883 | 0.883 | 0.883 |
| Coupled CS precision | | | | | | | | | | | | |
| Signal-3L 4.0 (Foldseek) | 0.912 | 0.912 | 0.912 | 0.912 | 0.960 | 0.961 | 0.961 | 0.964 | 0.970 | 0.970 | 0.970 | 0.970 |
| Signal-3L 4.0 (FoldExplorer) | **0.924** | **0.924** | **0.924** | **0.924** | **0.968** | **0.968** | **0.968** | **0.972** | **0.973** | **0.973** | **0.974** | **0.974** |
| SignalP 6.0 (Lit.) | 0.583 | 0.583 | 0.583 | 0.583 | 0.913 | 0.913 | 0.917 | 0.925 | 0.929 | 0.938 | 0.938 | 0.938 |
| Coupled CS F1 score | | | | | | | | | | | | |
| Signal-3L 4.0 (Foldseek) | **0.894** | **0.894** | **0.894** | **0.894** | **0.948** | **0.949** | **0.949** | **0.952** | 0.939 | 0.939 | 0.939 | 0.939 |
| Signal-3L 4.0 (FoldExplorer) | 0.881 | 0.881 | 0.881 | 0.881 | **0.948** | 0.948 | 0.948 | **0.952** | **0.943** | **0.943** | **0.944** | **0.944** |
| SignalP 6.0 (Lit.) | 0.667 | 0.667 | 0.667 | 0.667 | 0.881 | 0.881 | 0.886 | 0.893 | 0.905 | 0.910 | 0.910 | 0.910 |

**Table S7.** Coupled cleavage-site prediction performance of Signal-3L 4.0 variants and SignalP 6.0 on the benchmark dataset for Tat/SPI signal peptides.

| Methods | ARCHAEA | | | | NEGATIVE | | | | POSITIVE | | | |
| --- | --- | --- | --- | --- | --- | --- | --- | --- | --- | --- | --- | --- |
|  | ±0 | ±1 | ±2 | ±3 | ±0 | ±1 | ±2 | ±3 | ±0 | ±1 | ±2 | ±3 |
| Coupled CS recall | | | | | | | | | | | | |
| Signal-3L 4.0 (Foldseek) | 0.530 | 0.629 | **0.689** | **0.697** | 0.656 | 0.694 | 0.719 | 0.741 | **0.563** | **0.570** | 0.588 | 0.592 |
| Signal-3L 4.0 (FoldExplorer) | **0.538** | **0.644** | 0.682 | 0.689 | 0.689 | 0.728 | 0.769 | 0.789 | 0.552 | 0.561 | 0.595 | 0.608 |
| SignalP 6.0 (Lit.) | 0.333 | 0.444 | 0.444 | 0.444 | **0.706** | **0.765** | **0.784** | **0.804** | 0.556 | 0.556 | **0.667** | **0.667** |
| Coupled CS precision | | | | | | | | | | | | |
| Signal-3L 4.0 (Foldseek) | **0.617** | **0.730** | **0.805** | **0.816** | 0.677 | 0.716 | 0.742 | 0.765 | 0.662 | 0.669 | 0.690 | 0.695 |
| Signal-3L 4.0 (FoldExplorer) | 0.566 | 0.676 | 0.717 | 0.727 | 0.668 | 0.707 | 0.747 | 0.766 | 0.613 | 0.623 | 0.661 | 0.676 |
| SignalP 6.0 (Lit.) | 0.375 | 0.500 | 0.500 | 0.500 | **0.679** | **0.736** | **0.755** | **0.774** | **0.714** | **0.714** | **0.857** | **0.857** |
| Coupled CS F1 score | | | | | | | | | | | | |
| Signal-3L 4.0 (Foldseek) | **0.570** | **0.675** | **0.743** | **0.752** | 0.667 | 0.705 | 0.731 | 0.753 | 0.608 | 0.616 | 0.635 | 0.640 |
| Signal-3L 4.0 (FoldExplorer) | 0.552 | 0.659 | 0.699 | 0.708 | 0.678 | 0.717 | 0.758 | 0.778 | 0.581 | 0.590 | 0.626 | 0.640 |
| SignalP 6.0 (Lit.) | 0.353 | 0.470 | 0.470 | 0.470 | **0.692** | **0.750** | **0.769** | **0.789** | **0.625** | **0.625** | **0.750** | **0.750** |

**Table S8.** Unconditional and conditional cleavage-site predictioin performance of Signal-3L 4.0 variants and SignalP 6.0 on the benchmark dataset for Sec/SPI signal peptides. Unconditional recall, precision, and F1-score are reported under tolerance windows of ±0, ±1, ±2, and ±3 residues for each organism group, together with conditional CS F1/accuracy under the same tolerance windows. Two Signal-3L 4.0 variants are shown, using Foldseek-based and FoldExplorer-based structural representations, respectively. Bold indicates the best performance among the compared methods.

| Methods | ARCHAEA | | | | EUKARYA | | | | NEGATIVE | | | | POSITIVE | | | |
| --- | --- | --- | --- | --- | --- | --- | --- | --- | --- | --- | --- | --- | --- | --- | --- | --- |
|  | ±0 | ±1 | ±2 | ±3 | ±0 | ±1 | ±2 | ±3 | ±0 | ±1 | ±2 | ±3 | ±0 | ±1 | ±2 | ±3 |
| Unconditional CS recall | | | | | | | | | | | | | | | | |
| Signal-3L 4.0 (Foldseek) | 0.643 | 0.707 | 0.730 | 0.757 | 0.721 | 0.780 | 0.810 | 0.835 | 0.682 | 0.725 | 0.766 | 0.783 | **0.847** | **0.860** | **0.860** | **0.860** |
| Signal-3L 4.0 (FoldExplorer) | **0.663** | **0.733** | **0.767** | **0.790** | 0.735 | **0.790** | **0.818** | **0.836** | **0.719** | **0.755** | **0.794** | **0.805** | **0.847** | 0.853 | 0.853 | 0.853 |
| Unconditional CS precision | | | | | | | | | | | | | | | | |
| Signal-3L 4.0 (Foldseek) | **0.729** | **0.801** | **0.829** | **0.856** | **0.683** | **0.732** | **0.759** | **0.778** | **0.742** | **0.801** | **0.832** | **0.849** | 0.784 | 0.797 | 0.803 | 0.803 |
| Signal-3L 4.0 (FoldExplorer) | 0.691 | 0.764 | 0.800 | 0.838 | 0.654 | 0.697 | 0.722 | 0.737 | 0.706 | 0.773 | 0.815 | 0.834 | **0.829** | **0.847** | **0.865** | **0.869** |
| Unconditional CS F1 score | | | | | | | | | | | | | | | | |
| Signal-3L 4.0 (Foldseek) | **0.683** | **0.751** | 0.776 | 0.803 | **0.702** | **0.755** | **0.784** | **0.806** | 0.711 | 0.761 | 0.798 | 0.814 | 0.814 | 0.827 | 0.830 | 0.830 |
| Signal-3L 4.0 (FoldExplorer) | 0.677 | 0.748 | **0.783** | **0.813** | 0.692 | 0.741 | 0.767 | 0.783 | **0.712** | **0.764** | **0.804** | **0.819** | **0.838** | **0.850** | **0.859** | **0.861** |
| Conditional CS F1 score / Accuracy | | | | | | | | | | | | | | | | |
| Signal-3L 4.0 (Foldseek) | **0.755** | **0.830** | **0.859** | **0.887** | 0.790 | **0.846** | **0.877** | **0.900** | **0.836** | **0.885** | **0.920** | **0.935** | 0.916 | 0.932 | 0.932 | 0.932 |
| Signal-3L 4.0 (FoldExplorer) | 0.727 | 0.804 | 0.843 | 0.869 | **0.791** | 0.843 | 0.873 | 0.891 | 0.830 | 0.871 | 0.906 | 0.919 | **0.938** | **0.945** | **0.945** | **0.945** |

**Table S9.** Unconditional and conditional cleavage-site prediction performance of Signal-3L 4.0 variants and SignalP 6.0 on the benchmark dataset for Sec/SPII signal peptides.

| Methods | ARCHAEA | | | | NEGATIVE | | | | POSITIVE | | | |
| --- | --- | --- | --- | --- | --- | --- | --- | --- | --- | --- | --- | --- |
|  | ±0 | ±1 | ±2 | ±3 | ±0 | ±1 | ±2 | ±3 | ±0 | ±1 | ±2 | ±3 |
| Unconditional CS recall | | | | | | | | | | | | |
| Signal-3L 4.0 (Foldseek) | **0.930** | **0.965** | **0.965** | **0.965** | **0.947** | **0.952** | **0.953** | **0.958** | 0.931 | 0.932 | 0.933 | 0.934 |
| Signal-3L 4.0 (FoldExplorer) | 0.895 | 0.930 | 0.930 | 0.947 | 0.937 | 0.947 | 0.950 | 0.957 | **0.931** | **0.934** | **0.939** | **0.942** |
| Unconditional CS precision | | | | | | | | | | | | |
| Signal-3L 4.0 (Foldseek) | 0.933 | 0.933 | 0.933 | 0.933 | 0.962 | 0.963 | 0.966 | 0.970 | 0.969 | 0.969 | 0.969 | 0.969 |
| Signal-3L 4.0 (FoldExplorer) | **0.941** | **0.941** | **0.941** | **0.941** | **0.972** | **0.972** | **0.974** | **0.978** | **0.972** | **0.972** | **0.973** | **0.973** |
| Unconditional CS F1 score | | | | | | | | | | | | |
| Signal-3L 4.0 (Foldseek) | **0.931** | **0.949** | **0.949** | **0.949** | **0.954** | 0.958 | 0.959 | 0.964 | 0.950 | 0.950 | 0.950 | 0.951 |
| Signal-3L 4.0 (FoldExplorer) | 0.917 | 0.935 | 0.935 | 0.944 | **0.954** | **0.959** | **0.962** | **0.967** | **0.951** | **0.953** | **0.956** | **0.957** |
| Conditional CS F1 score / Accuracy | | | | | | | | | | | | |
| Signal-3L 4.0 (Foldseek) | **1** | **1** | **1** | **1** | 0.988 | 0.989 | 0.989 | 0.992 | 0.981 | 0.981 | 0.981 | 0.981 |
| Signal-3L 4.0 (FoldExplorer) | **1** | **1** | **1** | **1** | **0.991** | **0.991** | **0.991** | **0.995** | **0.983** | **0.983** | **0.984** | **0.984** |

**Table S10.** Unconditional and conditional cleavage-site prediction performance of Signal-3L 4.0 variants and SignalP 6.0 on the benchmark dataset for Tat/SPI signal peptides.

| Methods | ARCHAEA | | | | NEGATIVE | | | | POSITIVE | | | |
| --- | --- | --- | --- | --- | --- | --- | --- | --- | --- | --- | --- | --- |
|  | ±0 | ±1 | ±2 | ±3 | ±0 | ±1 | ±2 | ±3 | ±0 | ±1 | ±2 | ±3 |
| Unconditional CS recall | | | | | | | | | | | | |
| Signal-3L 4.0 (Foldseek) | 0.530 | 0.629 | **0.689** | 0.697 | 0.668 | 0.706 | 0.731 | 0.753 | 0.635 | 0.651 | 0.673 | 0.678 |
| Signal-3L 4.0 (FoldExplorer) | **0.538** | **0.644** | 0.682 | **0.705** | 0.706 | 0.745 | 0.786 | 0.806 | 0.619 | 0.633 | 0.671 | 0.685 |
| Unconditional CS precision | | | | | | | | | | | | |
| Signal-3L 4.0 (Foldseek) | **0.630** | **0.770** | **0.845** | **0.856** | 0.694 | 0.736 | 0.763 | 0.790 | 0.697 | 0.705 | 0.726 | 0.734 |
| Signal-3L 4.0 (FoldExplorer) | 0.604 | 0.744 | 0.785 | 0.794 | 0.702 | 0.750 | 0.794 | 0.817 | 0.647 | 0.660 | 0.703 | 0.722 |
| Unconditional CS F1 score | | | | | | | | | | | | |
| Signal-3L 4.0 (Foldseek) | **0.576** | **0.692** | **0.759** | **0.768** | 0.681 | 0.721 | 0.747 | 0.771 | 0.665 | 0.677 | 0.699 | 0.705 |
| Signal-3L 4.0 (FoldExplorer) | 0.569 | 0.690 | 0.730 | 0.747 | 0.704 | 0.747 | 0.790 | 0.812 | 0.633 | 0.646 | 0.687 | 0.703 |
| Conditional CS F1 score / Accuracy | | | | | | | | | | | | |
| Signal-3L 4.0 (Foldseek) | **0.644** | **0.762** | **0.840** | **0.850** | 0.715 | 0.756 | 0.784 | 0.808 | 0.698 | 0.706 | 0.728 | 0.734 |
| Signal-3L 4.0 (FoldExplorer) | 0.569 | 0.690 | 0.730 | 0.747 | 0.704 | 0.747 | 0.790 | 0.812 | 0.633 | 0.646 | 0.687 | 0.703 |

**Table S11.** Signal peptide classification performance (MCC1 and MCC2) of different baseline configurations and SignalP 6.0 on the benchmark dataset (ablation study).

| **Sec/SPI (SP)** | | | | | | | | | | | |
| --- | --- | --- | --- | --- | --- | --- | --- | --- | --- | --- | --- |
| Baseline methods | ARCHAEA | | EUKARYA | NEGATIVE | | | POSITIVE | |  |  |  |
|  | MCC1 | MCC2 | MCC1 | MCC1 | MCC2 | | MCC1 | MCC2 |  |  |  |
| ESMGearNet+CRF | **0.804** | **0.728** | 0.799 | 0.808 | 0.476 | | 0.864 | 0.429 |  |  |  |
| SaProt+CRF | 0.707 | 0.652 | **0.902** | 0.802 | 0.592 | | **0.883** | 0.538 |  |  |  |
| ESM2+CRF | 0.728 | 0.694 | 0.817 | **0.832** | 0.640 | | 0.874 | 0.587 |  |  |  |
| SignalP 6.0 (Lit.) | 0.737 | **0.728** | 0.868 | 0.811 | **0.649** | | **0.878** | **0.734** |  |  |  |
| **Sec/SPII (LIPO)** | | | | | | | | | | | |
| Baseline methods | ARCHAEA | | - | NEGATIVE | | | POSITIVE | | | |  |
|  | MCC1 | MCC2 | - | MCC1 | MCC2 | | MCC1 | MCC2 |  |  |  |
| ESMGearNet+CRF | 0.820 | 0.670 | - | 0.632 | 0.668 | | 0.694 | 0.737 |  |  |  |
| SaProt+CRF | 0.811 | 0.679 | - | 0.789 | 0.817 | | 0.765 | 0.811 |  |  |  |
| ESM2+CRF | **0.889** | 0.703 | - | 0.777 | 0.804 | | 0.823 | 0.862 |  |  |  |
| SignalP 6.0 (Lit.) | 0.871 | **0.719** | - | **0.838** | **0.841** | | **0.894** | **0.893** |  |  |  |
| **Tat/SPI (TAT)** | | | | | | | | | | | |
| Baseline methods | ARCHAEA | | - | NEGATIVE | | POSITIVE | | | |  |  |
|  | MCC1 | MCC2 | - | MCC1 | MCC2 | | MCC1 | MCC2 | | | |
| ESMGearNet+CRF | 0.629 | 0.636 | - | 0.670 | 0.724 | | 0.564 | 0.615 | | | |
| SaProt+CRF | 0.732 | 0.662 | - | 0.752 | 0.775 | | 0.624 | 0.686 | | | |
| ESM2+CRF | 0.707 | 0.658 | - | 0.794 | 0.816 | | 0.608 | 0.646 | | | |
| SignalP 6.0 (Lit.) | **0.802** | **0.807** | - | **0.946** | **0.934** | | **0.788** | **0.806** | | | |

**Table S12.** Cleavage-site prediction performance (recall, precision, and F1-score) of different baseline configurations and SignalP 6.0 on the benchmark dataset for Sec/SPI signal peptides at different tolerance windows (ablation study).

| Baseline methods | ARCHAEA | | | | EUKARYA | | | | NEGATIVE | | | | POSITIVE | | | |
| --- | --- | --- | --- | --- | --- | --- | --- | --- | --- | --- | --- | --- | --- | --- | --- | --- |
|  | ±0 | ±1 | ±2 | ±3 | ±0 | ±1 | ±2 | ±3 | ±0 | ±1 | ±2 | ±3 | ±0 | ±1 | ±2 | ±3 |
| CS recall | | | | | | | | | | | | | | | | |
| ESMGearNet+CRF | **0.607** | **0.707** | **0.753** | **0.773** | 0.754 | 0.791 | **0.828** | 0.850 | **0.652** | **0.704** | **0.742** | **0.760** | 0.747 | 0.747 | 0.760 | 0.760 |
| SaProt+CRF | 0.470 | 0.570 | 0.590 | 0.623 | **0.763** | **0.795** | 0.820 | **0.851** | 0.618 | 0.637 | 0.670 | 0.680 | **0.807** | **0.807** | **0.807** | **0.807** |
| ESM2+CRF | 0.473 | 0.547 | 0.580 | 0.600 | 0.751 | 0.787 | 0.817 | 0.844 | 0.633 | 0.663 | 0.706 | 0.715 | 0.740 | 0.753 | 0.753 | 0.753 |
| SignalP 6.0 (Lit.) | 0.500 | 0.556 | 0.556 | 0.583 | 0.747 | 0.774 | 0.808 | 0.829 | 0.639 | 0.672 | 0.689 | 0.721 | 0.8 | 0.8 | 0.8 | 0.8 |
| CS precision | | | | | | | | | | | | | | | | |
| ESMGearNet+CRF | 0.519 | 0.605 | 0.646 | 0.663 | 0.627 | 0.659 | 0.689 | 0.707 | 0.290 | 0.312 | 0.327 | 0.336 | 0.195 | 0.195 | 0.199 | 0.199 |
| SaProt+CRF | 0.408 | 0.495 | 0.513 | 0.542 | 0.657 | 0.684 | 0.706 | 0.732 | 0.389 | 0.399 | 0.419 | 0.425 | 0.260 | 0.260 | 0.260 | 0.260 |
| ESM2+CRF | 0.585 | 0.685 | 0.726 | 0.746 | 0.563 | 0.590 | 0.613 | 0.633 | 0.450 | 0.470 | 0.500 | 0.506 | 0.355 | 0.359 | 0.359 | 0.359 |
| SignalP 6.0 (Lit.) | **0.643** | **0.714** | **0.714** | **0.750** | **0.661** | **0.685** | **0.715** | **0.733** | **0.534** | **0.562** | **0.575** | **0.603** | **0.632** | **0.622** | **0.632** | **0.632** |
| CS F1 score | | | | | | | | | | | | | | | | |
| ESMGearNet+CRF | 0.560 | **0.652** | **0.696** | **0.714** | 0.684 | 0.719 | 0.752 | 0.772 | 0.401 | 0.433 | 0.453 | 0.466 | 0.309 | 0.309 | 0.315 | 0.315 |
| SaProt+CRF | 0.437 | 0.530 | 0.549 | 0.580 | **0.706** | **0.735** | 0.758 | **0.787** | 0.477 | 0.491 | 0.515 | 0.523 | 0.393 | 0.393 | 0.393 | 0.393 |
| ESM2+CRF | 0.523 | 0.608 | 0.645 | 0.665 | 0.644 | 0.674 | 0.700 | 0.723 | 0.526 | 0.550 | 0.585 | 0.593 | 0.480 | 0.486 | 0.486 | 0.486 |
| SignalP 6.0 (Lit.) | **0.563** | 0.625 | 0.625 | 0.656 | 0.701 | 0.727 | **0.759** | 0.778 | **0.582** | **0.612** | **0.627** | **0.657** | **0.706** | **0.700** | **0.706** | **0.706** |

**Table S13.** Cleavage-site prediction performance (recall, precision, and F1-score) of different baseline configurations and SignalP 6.0 on the benchmark dataset for Sec/SPII signal peptides at different tolerance windows (ablation study).

| Baseline methods | ARCHAEA | | | | NEGATIVE | | | | POSITIVE | | | |
| --- | --- | --- | --- | --- | --- | --- | --- | --- | --- | --- | --- | --- |
|  | ±0 | ±1 | ±2 | ±3 | ±0 | ±1 | ±2 | ±3 | ±0 | ±1 | ±2 | ±3 |
| CS recall | | | | | | | | | | | | |
| ESMGearNet+CRF | 0.789 | 0.789 | 0.789 | 0.789 | 0.713 | 0.713 | 0.713 | 0.716 | 0.750 | 0.751 | 0.753 | 0.753 |
| SaProt+CRF | 0.737 | 0.737 | 0.737 | 0.737 | **0.865** | **0.865** | **0.865** | **0.868** | 0.812 | 0.815 | 0.815 | 0.815 |
| ESM2+CRF | **0.825** | **0.825** | **0.825** | **0.825** | 0.837 | 0.839 | 0.839 | 0.842 | 0.852 | 0.852 | 0.854 | 0.854 |
| SignalP 6.0 (Lit.) | 0.778 | 0.778 | 0.778 | 0.778 | 0.852 | 0.852 | 0.856 | 0.864 | **0.883** | **0.883** | **0.883** | **0.883** |
| CS precision | | | | | | | | | | | | |
| ESMGearNet+CRF | 0.629 | 0.629 | 0.629 | 0.629 | 0.905 | 0.906 | 0.906 | 0.909 | 0.910 | 0.912 | 0.913 | 0.913 |
| SaProt+CRF | 0.498 | 0.498 | 0.498 | 0.498 | 0.927 | 0.927 | 0.927 | 0.930 | 0.896 | 0.900 | 0.900 | 0.900 |
| ESM2+CRF | **0.639** | **0.639** | **0.639** | **0.639** | **0.928** | **0.930** | **0.931** | **0.934** | 0.922 | 0.922 | 0.924 | 0.924 |
| SignalP 6.0 (Lit.) | 0.583 | 0.583 | 0.583 | 0.583 | 0.913 | 0.913 | 0.917 | 0.925 | **0.929** | **0.938** | **0.938** | **0.938** |
| CS F1 score | | | | | | | | | | | | |
| ESMGearNet+CRF | 0.700 | 0.700 | 0.700 | 0.700 | 0.797 | 0.798 | 0.798 | 0.801 | 0.822 | 0.824 | 0.825 | 0.825 |
| SaProt+CRF | 0.594 | 0.594 | 0.594 | 0.594 | **0.895** | **0.895** | **0.895** | **0.898** | 0.852 | 0.856 | 0.856 | 0.856 |
| ESM2+CRF | **0.720** | **0.720** | **0.720** | **0.720** | 0.880 | 0.882 | 0.882 | 0.885 | 0.886 | 0.886 | 0.887 | 0.887 |
| SignalP 6.0 (Lit.) | 0.667 | 0.667 | 0.667 | 0.667 | 0.881 | 0.881 | 0.886 | 0.893 | **0.905** | **0.910** | **0.910** | **0.910** |

**Table S14.** Cleavage-site prediction performance (recall, precision, and F1-score) of different baseline configurations and SignalP 6.0 on the benchmark dataset for Tat/SPI signal peptides at different tolerance windows (ablation study).

| Baseline methods | ARCHAEA | | | | NEGATIVE | | | | POSITIVE | | | |
| --- | --- | --- | --- | --- | --- | --- | --- | --- | --- | --- | --- | --- |
|  | ±0 | ±1 | ±2 | ±3 | ±0 | ±1 | ±2 | ±3 | ±0 | ±1 | ±2 | ±3 |
| CS recall | | | | | | | | | | | | |
| ESMGearNet+CRF | 0.303 | 0.364 | 0.409 | 0.424 | 0.435 | 0.480 | 0.510 | 0.517 | 0.306 | 0.329 | 0.369 | 0.392 |
| SaProt+CRF | **0.371** | 0.432 | 0.455 | 0.455 | 0.471 | 0.495 | 0.519 | 0.543 | 0.329 | 0.351 | 0.403 | 0.410 |
| ESM2+CRF | 0.348 | 0.409 | **0.470** | **0.470** | 0.577 | 0.592 | 0.616 | 0.626 | 0.360 | 0.365 | 0.401 | 0.401 |
| SignalP 6.0 (Lit.) | 0.333 | **0.444** | 0.444 | 0.444 | **0.706** | **0.765** | **0.784** | **0.804** | **0.556** | **0.556** | **0.667** | **0.667** |
| CS precision | | | | | | | | | | | | |
| ESMGearNet+CRF | **0.463** | **0.572** | **0.646** | **0.680** | 0.558 | 0.618 | 0.661 | 0.667 | 0.504 | 0.534 | 0.601 | 0.642 |
| SaProt+CRF | 0.320 | 0.380 | 0.402 | 0.402 | 0.504 | 0.528 | 0.555 | 0.582 | 0.433 | 0.458 | 0.527 | 0.537 |
| ESM2+CRF | 0.433 | 0.513 | 0.585 | 0.585 | 0.628 | 0.642 | 0.667 | 0.679 | 0.469 | 0.474 | 0.526 | 0.526 |
| SignalP 6.0 (Lit.) | 0.375 | 0.500 | 0.500 | 0.500 | **0.679** | **0.736** | **0.755** | **0.774** | **0.714** | **0.714** | **0.857** | **0.857** |
| CS F1 score | | | | | | | | | | | | |
| ESMGearNet+CRF | 0.366 | 0.444 | 0.501 | 0.522 | 0.489 | 0.540 | 0.576 | 0.583 | 0.381 | 0.407 | 0.458 | 0.487 |
| SaProt+CRF | 0.344 | 0.404 | 0.427 | 0.427 | 0.487 | 0.511 | 0.536 | 0.562 | 0.374 | 0.398 | 0.457 | 0.465 |
| ESM2+CRF | **0.386** | **0.455** | **0.521** | **0.521** | 0.601 | 0.616 | 0.640 | 0.651 | 0.408 | 0.412 | 0.455 | 0.455 |
| SignalP 6.0 (Lit.) | 0.353 | 0.470 | 0.470 | 0.470 | **0.692** | **0.750** | **0.769** | **0.789** | **0.625** | **0.625** | **0.750** | **0.750** |

**Table S15.** Detailed classification performance (MCC1 and MCC2) of the controlled build-up experiments, evaluating the incremental contributions of our proposed modules (ESM2, sequence branch, structure fusion, and CB-LDAM) relative to SignalP 6.0 across different signal peptide types and organism groups.

| **Sec/SPI (SP)** | | | | | | | | | |
| --- | --- | --- | --- | --- | --- | --- | --- | --- | --- |
| Methods | Loss Type | ARCHAEA | | EUKARYA | NEGATIVE | | POSITIVE | | |
|  |  | MCC1 | MCC2 | MCC1 | MCC1 | MCC2 | MCC1 | MCC2 | |
| SignalP 6.0 (Lit.) | NLL + CRF | 0.737 | 0.728 | 0.868 | 0.811 | 0.649 | 0.878 | 0.734 | |
| ESM2+CRF | NLL + CRF | 0.728 | 0.694 | 0.817 | 0.832 | 0.640 | 0.874 | 0.587 | |
| + Sequence Branch | NLL + CRF | 0.817 | 0.795 | 0.839 | **0.873** | 0.733 | 0.936 | 0.653 | |
| + Structure & Co-Att. | NLL + CRF | **0.876** | **0.868** | 0.872 | 0.858 | **0.792** | **0.918** | **0.766** | |
| Full Model (Foldseek) | CB-LDAM + CRF | 0.848 | 0.852 | **0.881** | 0.832 | 0.781 | 0.914 | 0.749 | |
| **Sec/SPII (LIPO)** | | | | | | | | | |
| Methods | Loss Type | ARCHAEA | | - | NEGATIVE | | POSITIVE | | |
|  |  | MCC1 | MCC2 | - | MCC1 | MCC2 | MCC1 | MCC2 | |
| SignalP 6.0 (Lit.) | NLL + CRF | 0.871 | 0.719 | - | 0.838 | 0.841 | 0.894 | 0.893 | |
| ESM2+CRF | NLL + CRF | 0.889 | 0.703 | - | 0.777 | 0.804 | 0.823 | 0.862 | |
| + Sequence Branch | NLL + CRF | 0.869 | 0.780 | - | 0.831 | 0.869 | 0.844 | 0.880 | |
| + Structure & Co-Att. | NLL + CRF | 0.919 | 0.868 | - | 0.902 | **0.917** | **0.904** | **0.918** | |
| Full Model (Foldseek) | CB-LDAM + CRF | **0.925** | **0.874** | **-** | **0.905** | 0.911 | 0.892 | 0.914 | |
| **Tat/SPI (TAT)** | | | | | | | | | |
| Methods | Loss Type | ARCHAEA | | - | NEGATIVE | | POSITIVE | |  |
|  |  | MCC1 | MCC2 | - | MCC1 | MCC2 | MCC1 | MCC2 |  |
| SignalP 6.0 (Lit.) | NLL + CRF | 0.802 | 0.807 | - | **0.946** | **0.934** | 0.788 | 0.806 |  |
| ESM2+CRF | NLL + CRF | 0.707 | 0.658 | - | 0.794 | 0.816 | 0.608 | 0.646 |  |
| + Sequence Branch | NLL + CRF | 0.730 | 0.720 | - | 0.806 | 0.831 | 0.667 | 0.720 |  |
| + Structure & Co-Att. | NLL + CRF | 0.860 | 0.825 | - | 0.901 | 0.907 | 0.806 | 0.836 |  |
| Full Model (Foldseek) | CB-LDAM + CRF | **0.884** | **0.868** | - | 0.924 | 0.927 | **0.833** | **0.854** |  |

**Table S16.** Detailed cleavage-site prediction metrics (Recall, Precision, and F1 score) of the controlled build-up experiments for Sec/SPI proteins. Results are evaluated at tolerance windows ranging from ±0 to ±3 residues to demonstrate the incremental localization gains of each architectural component.

| Methods | Loss Type | ARCHAEA | | | | EUKARYA | | | | NEGATIVE | | | | POSITIVE | | | |  |
| --- | --- | --- | --- | --- | --- | --- | --- | --- | --- | --- | --- | --- | --- | --- | --- | --- | --- | --- |
|  |  | ±0 | ±1 | ±2 | ±3 | ±0 | ±1 | ±2 | ±3 | ±0 | ±1 | ±2 | ±3 | ±0 | ±1 | ±2 | ±3 |  |
| CS recall | | | | | | | | | | | | | | | | | | |
| SignalP 6.0 (Lit.) | NLL + CRF | 0.500 | 0.556 | 0.556 | 0.583 | 0.747 | 0.774 | 0.808 | 0.829 | 0.639 | 0.672 | 0.689 | 0.721 | 0.8 | 0.8 | 0.8 | 0.8 |  |
| ESM2+CRF | NLL + CRF | 0.473 | 0.547 | 0.580 | 0.600 | **0.751** | **0.787** | **0.817** | 0.844 | 0.633 | 0.663 | 0.706 | 0.715 | 0.74 | 0.753 | 0.753 | 0.753 |  |
| + Sequence Branch | NLL + CRF | 0.553 | 0.627 | 0.650 | 0.673 | 0.710 | 0.750 | 0.773 | 0.797 | **0.702** | 0.713 | 0.742 | 0.749 | 0.773 | 0.8 | 0.8 | 0.8 |  |
| + Structure & Co-Att. | NLL + CRF | **0.647** | **0.727** | **0.747** | **0.770** | 0.729 | 0.777 | 0.803 | 0.831 | 0.691 | **0.730** | **0.772** | **0.787** | **0.820** | **0.820** | **0.820** | **0.820** |  |
| Full Model (Foldseek) | CB-LDAM + CRF | 0.617 | 0.680 | 0.703 | 0.727 | 0.719 | 0.770 | 0.799 | 0.819 | 0.670 | 0.710 | 0.738 | 0.749 | 0.793 | 0.807 | 0.807 | 0.807 |  |
| CS precision | | | | | | | | | | | | | | | | | | |
| SignalP 6.0 (Lit.) | NLL + CRF | 0.643 | 0.714 | 0.714 | 0.750 | 0.661 | 0.685 | 0.715 | 0.733 | 0.534 | 0.562 | 0.575 | 0.603 | 0.632 | 0.632 | 0.632 | 0.632 |  |
| ESM2+CRF | NLL + CRF | 0.585 | 0.685 | 0.726 | 0.746 | 0.563 | 0.590 | 0.613 | 0.633 | 0.450 | 0.470 | 0.500 | 0.506 | 0.355 | 0.359 | 0.359 | 0.359 |  |
| + Sequence Branch | NLL + CRF | 0.677 | 0.767 | 0.792 | 0.821 | 0.684 | 0.722 | 0.745 | 0.768 | 0.605 | 0.615 | 0.639 | 0.645 | 0.454 | 0.469 | 0.469 | 0.469 |  |
| + Structure & Co-Att. | NLL + CRF | 0.730 | **0.820** | **0.843** | **0.868** | 0.676 | 0.720 | 0.744 | 0.770 | 0.661 | 0.698 | 0.739 | 0.753 | **0.635** | **0.635** | **0.635** | **0.635** |  |
| Full Model (Foldseek) | CB-LDAM + CRF | **0.734** | 0.807 | 0.835 | 0.862 | **0.695** | **0.744** | **0.772** | **0.791** | **0.698** | **0.738** | **0.767** | **0.779** | 0.615 | 0.624 | 0.624 | 0.624 |  |
| CS F1 score | | | | | | | | | | | | | | | | | | |
| SignalP 6.0 (Lit.) | NLL + CRF | 0.563 | 0.625 | 0.625 | 0.656 | 0.701 | 0.727 | 0.759 | 0.778 | 0.582 | 0.612 | 0.627 | 0.657 | 0.706 | 0.706 | 0.706 | 0.706 |  |
| ESM2+CRF | NLL + CRF | 0.523 | 0.608 | 0.645 | 0.665 | 0.644 | 0.674 | 0.700 | 0.723 | 0.526 | 0.550 | 0.585 | 0.593 | 0.480 | 0.486 | 0.486 | 0.486 |  |
| + Sequence Branch | NLL + CRF | 0.609 | 0.690 | 0.714 | 0.740 | 0.697 | 0.736 | 0.759 | 0.782 | 0.650 | 0.661 | 0.686 | 0.693 | 0.572 | 0.591 | 0.591 | 0.591 |  |
| + Structure & Co-Att. | NLL + CRF | **0.686** | **0.770** | **0.792** | **0.816** | 0.702 | 0.748 | 0.772 | 0.799 | 0.676 | 0.714 | **0.755** | **0.769** | **0.716** | **0.716** | **0.716** | **0.716** |  |
| Full Model (Foldseek) | CB-LDAM + CRF | 0.670 | 0.738 | 0.763 | 0.789 | **0.707** | **0.757** | **0.785** | **0.805** | **0.684** | **0.724** | 0.752 | 0.764 | 0.693 | 0.704 | 0.704 | 0.704 |  |

**Table S17.** Detailed cleavage-site prediction metrics (Recall, Precision, and F1 score) of the controlled build-up experiments for Sec/SPII proteins. Results are evaluated at tolerance windows ranging from ±0 to ±3 residues to demonstrate the incremental localization gains of each architectural component.

| Methods | Loss Type | ARCHAEA | | | | NEGATIVE | | | | POSITIVE | | | | |
| --- | --- | --- | --- | --- | --- | --- | --- | --- | --- | --- | --- | --- | --- | --- |
|  |  | ±0 | ±1 | ±2 | ±3 | ±0 | ±1 | ±2 | ±3 | ±0 | ±1 | ±2 | ±3 | |
| CS recall | | | | | | | | | | | | | |  |
| SignalP 6.0 (Lit.) | NLL + CRF | 0.778 | 0.778 | 0.778 | 0.778 | 0.852 | 0.852 | 0.856 | 0.864 | 0.883 | 0.883 | 0.883 | 0.883 | |
| ESM2+CRF | NLL + CRF | 0.825 | 0.825 | 0.825 | 0.825 | 0.837 | 0.839 | 0.839 | 0.842 | 0.852 | 0.852 | 0.854 | 0.854 | |
| + Sequence Branch | NLL + CRF | 0.825 | 0.825 | 0.825 | 0.825 | 0.883 | 0.883 | 0.884 | 0.887 | 0.862 | 0.862 | 0.863 | 0.863 | |
| + Structure & Co-Att. | NLL + CRF | **0.877** | **0.877** | **0.877** | **0.877** | 0.935 | 0.935 | 0.935 | 0.939 | **0.915** | **0.915** | **0.915** | **0.915** | |
| Full Model (Foldseek) | CB-LDAM + CRF | **0.877** | **0.877** | **0.877** | **0.877** | **0.936** | **0.937** | **0.937** | **0.940** | 0.910 | 0.910 | 0.910 | 0.910 | |
| CS precision | | | | | | | | | | | | | |  |
| SignalP 6.0 (Lit.) | NLL + CRF | 0.583 | 0.583 | 0.583 | 0.583 | 0.913 | 0.913 | 0.917 | 0.925 | 0.929 | 0.938 | 0.938 | 0.938 | |
| ESM2+CRF | NLL + CRF | 0.639 | 0.639 | 0.639 | 0.639 | 0.928 | 0.930 | 0.931 | 0.934 | 0.922 | 0.922 | 0.924 | 0.924 | |
| + Sequence Branch | NLL + CRF | 0.768 | 0.768 | 0.768 | 0.768 | **0.967** | **0.967** | **0.968** | **0.970** | 0.965 | 0.965 | 0.967 | 0.967 | |
| + Structure & Co-Att. | NLL + CRF | 0.871 | 0.871 | 0.871 | 0.871 | 0.966 | 0.966 | 0.966 | **0.970** | 0.963 | 0.963 | 0.963 | 0.963 | |
| Full Model (Foldseek) | CB-LDAM + CRF | **0.912** | **0.912** | **0.912** | **0.912** | 0.960 | 0.961 | 0.961 | 0.964 | **0.970** | **0.970** | **0.970** | **0.970** | |
| CS F1 score | | | | | | | | | | | | | |  |
| SignalP 6.0 (Lit.) | NLL + CRF | 0.667 | 0.667 | 0.667 | 0.667 | 0.881 | 0.881 | 0.886 | 0.893 | 0.905 | 0.910 | 0.910 | 0.910 | |
| ESM2+CRF | NLL + CRF | 0.720 | 0.720 | 0.720 | 0.720 | 0.880 | 0.882 | 0.882 | 0.885 | 0.886 | 0.886 | 0.887 | 0.887 | |
| + Sequence Branch | NLL + CRF | 0.795 | 0.795 | 0.795 | 0.795 | 0.924 | 0.924 | 0.924 | 0.927 | 0.910 | 0.910 | 0.912 | 0.912 | |
| + Structure & Co-Att. | NLL + CRF | 0.874 | 0.874 | 0.874 | 0.874 | **0.950** | **0.950** | **0.950** | **0.954** | **0.939** | **0.939** | **0.939** | **0.939** | |
| Full Model (Foldseek) | CB-LDAM + CRF | **0.894** | **0.894** | **0.894** | **0.894** | 0.948 | 0.949 | 0.949 | 0.952 | **0.939** | **0.939** | **0.939** | **0.939** | |

**Table S18.** Detailed cleavage-site prediction metrics (Recall, Precision, and F1 score) of the controlled build-up experiments for Tat/SPI proteins. Results are evaluated at tolerance windows ranging from ±0 to ±3 residues to demonstrate the incremental localization gains of each architectural component.

| Methods | Loss Type | ARCHAEA | | | | NEGATIVE | | | | POSITIVE | | | | |
| --- | --- | --- | --- | --- | --- | --- | --- | --- | --- | --- | --- | --- | --- | --- |
|  |  | ±0 | ±1 | ±2 | ±3 | ±0 | ±1 | ±2 | ±3 | ±0 | ±1 | ±2 | ±3 | |
| CS recall | | | | | | | | | | | | | |  |
| SignalP 6.0 (Lit.) | NLL + CRF | 0.333 | 0.444 | 0.444 | 0.444 | **0.706** | **0.765** | **0.784** | **0.804** | 0.556 | 0.556 | **0.667** | **0.667** | |
| ESM2+CRF | NLL + CRF | 0.348 | 0.409 | 0.470 | 0.470 | 0.577 | 0.592 | 0.616 | 0.626 | 0.360 | 0.365 | 0.401 | 0.401 | |
| + Sequence Branch | NLL + CRF | 0.386 | 0.447 | 0.500 | 0.500 | 0.583 | 0.607 | 0.628 | 0.641 | 0.390 | 0.394 | 0.430 | 0.430 | |
| + Structure & Co-Att. | NLL + CRF | 0.523 | 0.629 | 0.659 | 0.659 | 0.648 | 0.679 | 0.711 | 0.738 | 0.536 | 0.538 | 0.572 | 0.581 | |
| Full Model (Foldseek) | CB-LDAM + CRF | **0.530** | **0.629** | **0.689** | **0.697** | 0.656 | 0.694 | 0.719 | 0.741 | **0.563** | **0.570** | 0.588 | 0.592 | |
| CS precision | | | | | | | | | | | | | |  |
| SignalP 6.0 (Lit.) | NLL + CRF | 0.375 | 0.500 | 0.500 | 0.500 | 0.679 | 0.736 | 0.755 | 0.774 | **0.714** | **0.714** | **0.857** | **0.857** | |
| ESM2+CRF | NLL + CRF | 0.433 | 0.513 | 0.585 | 0.585 | 0.628 | 0.642 | 0.667 | 0.679 | 0.469 | 0.474 | 0.526 | 0.526 | |
| + Sequence Branch | NLL + CRF | 0.596 | 0.682 | 0.761 | 0.761 | **0.731** | **0.762** | **0.787** | **0.804** | 0.643 | 0.649 | 0.717 | 0.717 | |
| + Structure & Co-Att. | NLL + CRF | 0.601 | 0.719 | 0.760 | 0.760 | 0.688 | 0.721 | 0.755 | 0.786 | 0.672 | 0.674 | 0.716 | 0.727 | |
| Full Model (Foldseek) | CB-LDAM + CRF | **0.617** | **0.730** | **0.805** | **0.816** | 0.677 | 0.716 | 0.742 | 0.765 | 0.662 | 0.669 | 0.690 | 0.695 | |
| CS F1 score | | | | | | | | | | | | | |  |
| SignalP 6.0 (Lit.) | NLL + CRF | 0.353 | 0.470 | 0.470 | 0.470 | **0.692** | **0.750** | **0.769** | **0.789** | **0.625** | **0.625** | **0.750** | **0.750** | |
| ESM2+CRF | NLL + CRF | 0.386 | 0.455 | 0.521 | 0.521 | 0.601 | 0.616 | 0.640 | 0.651 | 0.408 | 0.412 | 0.455 | 0.455 | |
| + Sequence Branch | NLL + CRF | 0.469 | 0.540 | 0.604 | 0.604 | 0.649 | 0.676 | 0.698 | 0.713 | 0.485 | 0.490 | 0.538 | 0.538 | |
| + Structure & Co-Att. | NLL + CRF | 0.559 | 0.671 | 0.706 | 0.706 | 0.667 | 0.699 | 0.733 | 0.761 | 0.596 | 0.599 | 0.636 | 0.646 | |
| Full Model (Foldseek) | CB-LDAM + CRF | **0.570** | **0.675** | **0.743** | **0.752** | 0.667 | 0.705 | 0.731 | 0.753 | 0.608 | 0.616 | 0.635 | 0.640 | |

**Table S19.** Protein Sequence Updates in the SignalP 6.0 Training Set

| Old Accession | Old Info. | Updated Accession | Updated Info. |
| --- | --- | --- | --- |
| Q9QXA1 | EUKARYA, NO_SP | P0DW86 | EUKARYA, SP, CS=32 (taxID=10090) |
|  |  | P0DW87 | EUKARYA, NO_SP (taxID=10090) |
| Q8NG57 | EUKARYA, NO_SP | DELETED | - |
| B2G331 | EUKARYA, SP, CS=22 | B2G331 | EUKARYA, SP, CS=22 (taxID=175771) |
| P0DMJ5 | EUKARYA, SP, CS=22 | P0DMJ5 | EUKARYA, SP, CS=22 (taxID=175771) |
| C2R3K4 | POSITIVE, NO_SP | DELETED | - |
| P0A3N6 | NEGATIVE, LIPO, CS=16 | P0DW55 | NEGATIVE, LIPO, CS=16 (taxID=136) |
|  |  | Q0SLZ0 | NEGATIVE, LIPO, CS=16 (taxID=390236) |
| D3QL74 | NEGATIVE, NO_SP | DELETED | - |

**Table S20.** Distribution of the GOSP2024 Dataset

| Sec/SPI (SP) | | | | Sec/SPII (LIPO) | | | Tat/SPI (TAT) | | |
| --- | --- | --- | --- | --- | --- | --- | --- | --- | --- |
| EUKARYA | ARCHAEA | NEGATIVE | POSITIVE | ARCHAEA | NEGATIVE | POSITIVE | ARCHAEA | NEGATIVE | POSITIVE |
| 514 | 2 | 129 | 65 | 0 | 2 | 0 | 2 | 12 | 14 |

**Table S21.** List of protein UniProt accessions without AlphaFold-predicted structures in the SignalP training and benchmark test sets

| A2A9C3 | P36970 | Q868Z9 | A2AAJ9 | P38110 | Q8C8R3 | A8DYP0 | Q9Z0J5 | P19158 | Q9PKX2 |
| --- | --- | --- | --- | --- | --- | --- | --- | --- | --- |
| P40806 | Q8I8U7 | A8ICS9 | P40872 | Q8IN94 | A8J666 | P42858 | P35555 | Q6ZRS2 | P21359 |
| Q8IZQ1 | E9Q394 | P46013 | Q8IZT6 | E9QAM5 | P46448 | Q8N3K9 | Q7Z2C4 | Q6U6H1 | Q5VT06 |
| F4HZB2 | P49792 | Q8R0W0 | F4JTS8 | P51587 | Q8SX83 | F8VPN2 | Q9Z4Q7 | Q9VDW6 | Q9QZZ4 |
| P55073 | Q8TD26 | G4SLH0 | P58107 | Q8WUY3 | G5EEV2 | P78527 | P36022 | Q9VC56 | P13611 |
| Q8WXX0 | H2QII6 | Q01484 | Q8WZ42 | O01761 | Q02224 | Q8X235 | Q7Z6Z7 | P24126 | Q62504 |
| O13290 | Q03001 | Q93442 | O24310 | Q03164 | Q96Q15 | O31782 | Q9ZBF6 | P20659 | P18963 |
| Q03280 | Q96RL7 | O60229 | Q04652 | Q96T58 | O75592 | Q04826 | Q709C8 | P19137 | Q6KC79 |
| Q97V37 | O75691 | Q05470 | Q99MD6 | O83276 | Q0KL02 | Q9BVL4 | Q9Y4A5 | Q6VNB8 | Q9U943 |
| O83774 | Q12019 | Q9GV28 | O87444 | Q12802 | Q9I7U4 | O94248 | P24183 | P15924 | Q9VCA8 |
| Q13315 | Q9JJ79 | O95359 | Q14315 | Q9JLC3 | O95613 | Q15149 | Q7TMY8 | Q6KCD5 | Q9P7T1 |
| Q9JLT4 | P01891 | Q15911 | Q9N2I8 | P03989 | Q20497 | Q9NDJ2 | Q9Y8I8 | Q63HN8 | P15822 |
| P04229 | Q23551 | Q9NNW7 | P04275 | Q24119 | Q9NR48 | P05790 | P25054 | Q9V496 | Q5VST9 |
| Q3KNY0 | Q9NU22 | P0DW55 | Q3L8U1 | Q9NZJ4 | P0DW86 | Q45VK7 | Q7YZH1 | Q9SMH5 | Q9QZR8 |
| Q9P2D1 | P0DW87 | Q5TBA9 | Q9P2P6 | P11531 | P14217 | Q61001 |  |  |  |

**Table S22.** Full per-sequence prediction results (signal peptide type and cleavage site) of Signal-3L 4.0 and three recent baseline models (SignalP 6.0, TSignal, USPNet) on the independent test set of 740 protein sequences.

| accession | kingdom | Groundtruth | | Signal-3L 4.0 (Fold-seek) | | Signal-3L 4.0 (Fold-explorer) | | SignalP 6.0 | | TSignal | | USPNet | | |
| --- | --- | --- | --- | --- | --- | --- | --- | --- | --- | --- | --- | --- | --- | --- |
|  |  | SP type | CS index | SP type | CS index | SP type | CS index | SP type | CS index | SP type | CS index | SP type | CS index |  |
| A0A7J6K7Y0 | EUKARYA | SP | 70 | NO_SP | -1 | NO_SP | -1 | NO_SP | -1 | NO_SP | -1 | NO_SP | -1 |  |
| A0A7J6K7I9 | EUKARYA | SP | 64 | NO_SP | -1 | NO_SP | -1 | NO_SP | -1 | NO_SP | -1 | NO_SP | -1 |  |
| P55203 | EUKARYA | SP | 56 | NO_SP | -1 | NO_SP | -1 | NO_SP | -1 | NO_SP | -1 | NO_SP | -1 |  |
| Q9FXT4 | EUKARYA | SP | 55 | SP | 33 | SP | 33 | SP | 33 | SP | 33 | SP | 33 |  |
| Q55U20 | EUKARYA | SP | 51 | SP | 20 | SP | 20 | SP | 20 | SP | 32 | SP | 20 |  |
| P05164 | EUKARYA | SP | 48 | SP | 48 | SP | 48 | SP | 48 | SP | 48 | SP | 48 |  |
| Q9BXI9 | EUKARYA | SP | 46 | NO_SP | -1 | NO_SP | -1 | NO_SP | -1 | SP | 46 | NO_SP | -1 |  |
| Q63415 | EUKARYA | SP | 45 | SP | 44 | SP | 45 | SP | 45 | SP | 45 | SP | 43 |  |
| P49256 | EUKARYA | SP | 44 | SP | 44 | SP | 44 | SP | 44 | SP | 44 | SP | 44 |  |
| Q06703 | EUKARYA | SP | 44 | SP | 25 | SP | 25 | SP | 25 | SP | 25 | NO_SP | -1 |  |
| Q9ZT66 | EUKARYA | SP | 43 | SP | 25 | SP | 25 | SP | 25 | SP | 25 | SP | 25 |  |
| Q10E50 | EUKARYA | SP | 43 | SP | 37 | SP | 37 | SP | 37 | SP | 37 | SP | 37 |  |
| Q6Z232 | EUKARYA | SP | 42 | SP | 21 | SP | 21 | SP | 21 | SP | 21 | SP | 21 |  |
| Q8NHP8 | EUKARYA | SP | 41 | SP | 41 | SP | 41 | SP | 41 | SP | 41 | NO_SP | -1 |  |
| P11717 | EUKARYA | SP | 40 | SP | 40 | SP | 40 | SP | 40 | SP | 40 | SP | 40 |  |
| Q9UIK5 | EUKARYA | SP | 40 | NO_SP | 33 | NO_SP | -1 | NO_SP | -1 | SP | 33 | NO_SP | -1 |  |
| Q9M653 | EUKARYA | SP | 39 | SP | 22 | SP | 22 | SP | 22 | SP | 22 | SP | 22 |  |
| P13689 | EUKARYA | SP | 38 | NO_SP | -1 | NO_SP | -1 | SP | 38 | SP | 38 | NO_SP | -1 |  |
| O75462 | EUKARYA | SP | 37 | SP | 37 | SP | 37 | SP | 37 | SP | 36 | SP | 36 |  |
| Q13421 | EUKARYA | SP | 36 | SP | 38 | SP | 38 | SP | 33 | SP | 38 | SP | 38 |  |
| Q04756 | EUKARYA | SP | 35 | SP | 35 | NO_SP | 35 | SP | 35 | NO_SP | -1 | SP | 32 |  |
| Q9NPF0 | EUKARYA | SP | 35 | SP | 35 | SP | 35 | SP | 35 | SP | 35 | SP | 35 |  |
| Q9Y251 | EUKARYA | SP | 35 | SP | 35 | SP | 35 | SP | 35 | SP | 35 | SP | 27 |  |
| P08818 | EUKARYA | SP | 34 | SP | 34 | TAT | 34 | SP | 34 | SP | 34 | SP | 33 |  |
| P08174 | EUKARYA | SP | 34 | SP | 34 | SP | 34 | SP | 34 | SP | 34 | SP | 34 |  |
| P36897 | EUKARYA | SP | 33 | SP | 33 | SP | 33 | SP | 33 | SP | 33 | SP | 33 |  |
| Q28270 | EUKARYA | SP | 33 | SP | 31 | SP | 31 | SP | 31 | SP | 33 | SP | 31 |  |
| Q8NBK3 | EUKARYA | SP | 33 | SP | 33 | SP | 33 | SP | 33 | SP | 33 | SP | 33 |  |
| Q653V7 | EUKARYA | SP | 33 | SP | 33 | SP | 33 | SP | 33 | SP | 33 | SP | 33 |  |
| O55232 | EUKARYA | SP | 32 | SP | 32 | SP | 32 | SP | 32 | SP | 32 | SP | 29 |  |
| P30040 | EUKARYA | SP | 32 | SP | 32 | SP | 32 | SP | 32 | SP | 32 | SP | 32 |  |
| Q10588 | EUKARYA | SP | 32 | SP | 32 | SP | 32 | SP | 32 | SP | 32 | SP | 29 |  |
| P01076 | EUKARYA | SP | 32 | SP | 23 | SP | 23 | SP | 23 | SP | 23 | SP | 23 |  |
| Q8BHP7 | EUKARYA | SP | 32 | SP | 27 | SP | 32 | SP | 32 | SP | 32 | SP | 29 |  |
| P09619 | EUKARYA | SP | 32 | SP | 32 | SP | 32 | SP | 32 | SP | 32 | SP | 32 |  |
| Q96F46 | EUKARYA | SP | 32 | SP | 32 | SP | 31 | SP | 32 | SP | 32 | SP | 31 |  |
| Q9H7M9 | EUKARYA | SP | 32 | SP | 32 | SP | 32 | SP | 32 | SP | 32 | SP | 32 |  |
| Q6UW01 | EUKARYA | SP | 32 | SP | 32 | SP | 32 | SP | 32 | SP | 32 | SP | 32 |  |
| Q8MMG9 | EUKARYA | SP | 32 | SP | 32 | SP | 32 | SP | 32 | SP | 30 | SP | 32 |  |
| Q8MI35 | EUKARYA | SP | 32 | SP | 32 | SP | 32 | SP | 32 | SP | 32 | SP | 32 |  |
| Q6IUU3 | EUKARYA | SP | 32 | SP | 32 | SP | 32 | SP | 32 | SP | 32 | SP | 32 |  |
| Q9NZ53 | EUKARYA | SP | 32 | LIPO | 29 | LIPO | 29 | LIPO | 29 | SP | 32 | NO_SP | -1 |  |
| P15309 | EUKARYA | SP | 32 | SP | 32 | SP | 32 | SP | 32 | SP | 32 | SP | 32 |  |
| P26006 | EUKARYA | SP | 32 | SP | 32 | SP | 32 | SP | 32 | SP | 32 | SP | 32 |  |
| P97710 | EUKARYA | SP | 31 | SP | 31 | SP | 31 | SP | 31 | SP | 31 | SP | 29 |  |
| P50895 | EUKARYA | SP | 31 | SP | 31 | SP | 31 | SP | 31 | SP | 31 | SP | 31 |  |
| P15196 | EUKARYA | SP | 31 | SP | 27 | SP | 20 | SP | 22 | SP | 22 | SP | 22 |  |
| O54861 | EUKARYA | SP | 31 | SP | 31 | SP | 31 | SP | 31 | SP | 31 | SP | 31 |  |
| D4A540 | EUKARYA | SP | 31 | SP | 31 | SP | 31 | SP | 31 | SP | 31 | SP | 31 |  |
| P49755 | EUKARYA | SP | 31 | SP | 31 | SP | 31 | SP | 31 | SP | 31 | SP | 31 |  |
| Q969H8 | EUKARYA | SP | 31 | SP | 31 | SP | 31 | SP | 31 | SP | 31 | SP | 31 |  |
| Q8BPM6 | EUKARYA | SP | 31 | NO_SP | -1 | NO_SP | -1 | NO_SP | -1 | NO_SP | -1 | NO_SP | -1 |  |
| B4FZ87 | EUKARYA | SP | 31 | SP | 34 | SP | 34 | SP | 32 | SP | 32 | SP | 32 |  |
| P29007 | EUKARYA | SP | 31 | SP | 31 | SP | 31 | SP | 31 | SP | 31 | SP | 24 |  |
| P49767 | EUKARYA | SP | 31 | SP | 29 | SP | 30 | SP | 29 | NO_SP | -1 | SP | 17 |  |
| O75629 | EUKARYA | SP | 31 | SP | 31 | SP | 31 | SP | 31 | SP | 31 | SP | 31 |  |
| O94907 | EUKARYA | SP | 31 | NO_SP | -1 | SP | 22 | SP | 30 | NO_SP | -1 | SP | 28 |  |
| F1N2K1 | EUKARYA | SP | 31 | SP | 31 | SP | 31 | SP | 31 | SP | 31 | SP | 31 |  |
| O96935 | EUKARYA | SP | 30 | SP | 20 | SP | -1 | NO_SP | -1 | NO_SP | -1 | SP | 70 |  |
| Q14112 | EUKARYA | SP | 30 | SP | 30 | SP | 30 | SP | 30 | SP | 30 | SP | 30 |  |
| Q9ERQ6 | EUKARYA | SP | 30 | SP | 33 | SP | 33 | SP | 29 | SP | 31 | SP | 30 |  |
| P22063 | EUKARYA | SP | 30 | SP | 30 | SP | 30 | SP | 30 | SP | 30 | SP | 28 |  |
| P01083 | EUKARYA | SP | 30 | SP | 21 | SP | 21 | SP | 21 | SP | 21 | SP | 21 |  |
| Q6UW56 | EUKARYA | SP | 30 | SP | 30 | SP | 30 | SP | 30 | SP | 30 | SP | 30 |  |
| Q96PD4 | EUKARYA | SP | 30 | SP | 30 | SP | 30 | SP | 30 | SP | 30 | SP | 28 |  |
| Q9BWV1 | EUKARYA | SP | 30 | SP | 30 | SP | 30 | SP | 30 | SP | 30 | SP | 28 |  |
| Q96I82 | EUKARYA | SP | 30 | SP | 30 | NO_SP | -1 | SP | 30 | SP | 30 | SP | 30 |  |
| Q8NBP7 | EUKARYA | SP | 30 | SP | 30 | SP | 30 | SP | 30 | SP | 30 | SP | 28 |  |
| Q8L5C6 | EUKARYA | SP | 30 | SP | 30 | SP | 30 | SP | 30 | SP | 30 | SP | 30 |  |
| Q64550 | EUKARYA | SP | 29 | SP | 29 | SP | 29 | SP | 29 | SP | 29 | SP | 29 |  |
| P04278 | EUKARYA | SP | 29 | SP | 29 | SP | 29 | SP | 29 | SP | 29 | SP | 29 |  |
| P13087 | EUKARYA | SP | 29 | SP | 28 | SP | 28 | SP | 28 | SP | 28 | SP | 21 |  |
| Q00657 | EUKARYA | SP | 29 | NO_SP | -1 | NO_SP | -1 | SP | 29 | SP | 29 | SP | 30 |  |
| P05231 | EUKARYA | SP | 29 | SP | 27 | SP | 27 | SP | 27 | SP | 27 | SP | 27 |  |
| O00478 | EUKARYA | SP | 29 | SP | 29 | SP | 29 | SP | 29 | SP | 29 | SP | 29 |  |
| Q96AP7 | EUKARYA | SP | 29 | SP | 29 | SP | 29 | SP | 29 | SP | 29 | SP | 29 |  |
| Q9UHF4 | EUKARYA | SP | 29 | SP | 29 | SP | 29 | SP | 29 | SP | 29 | SP | 29 |  |
| P85213 | EUKARYA | SP | 29 | SP | 29 | SP | 29 | SP | 29 | SP | 29 | SP | 29 |  |
| Q9SLY8 | EUKARYA | SP | 29 | SP | 28 | SP | 28 | SP | 28 | SP | 28 | SP | 28 |  |
| Q7Q7R8 | EUKARYA | SP | 29 | SP | 29 | SP | 29 | SP | 29 | SP | 29 | SP | 28 |  |
| P82900 | EUKARYA | SP | 29 | SP | 29 | SP | 29 | SP | 29 | SP | 29 | SP | 29 |  |
| O64411 | EUKARYA | SP | 28 | SP | 28 | SP | 28 | SP | 28 | SP | 28 | SP | 28 |  |
| O02853 | EUKARYA | SP | 28 | SP | 28 | SP | 28 | SP | 28 | SP | 28 | SP | 26 |  |
| Q28631 | EUKARYA | SP | 28 | SP | 28 | SP | 28 | SP | 28 | SP | 28 | SP | 28 |  |
| P55803 | EUKARYA | SP | 28 | SP | 28 | SP | 28 | SP | 29 | SP | 28 | SP | 28 |  |
| P40259 | EUKARYA | SP | 28 | SP | 28 | SP | 28 | SP | 28 | SP | 28 | SP | 28 |  |
| P11279 | EUKARYA | SP | 28 | SP | 28 | SP | 28 | SP | 28 | SP | 28 | SP | 28 |  |
| P21588 | EUKARYA | SP | 28 | SP | 28 | SP | 28 | SP | 28 | SP | 28 | SP | 28 |  |
| Q02083 | EUKARYA | SP | 28 | SP | 28 | SP | 28 | SP | 28 | SP | 28 | SP | 27 |  |
| P06276 | EUKARYA | SP | 28 | SP | 28 | SP | 28 | SP | 28 | SP | 28 | SP | 26 |  |
| Q8H8C7 | EUKARYA | SP | 28 | SP | 28 | SP | 28 | SP | 28 | SP | 28 | SP | 28 |  |
| Q9H4G1 | EUKARYA | SP | 28 | SP | 28 | SP | 28 | NO_SP | -1 | SP | 28 | NO_SP | -1 |  |
| P10619 | EUKARYA | SP | 28 | SP | 28 | NO_SP | 28 | SP | 28 | SP | 28 | SP | 26 |  |
| Q945U0 | EUKARYA | SP | 28 | SP | 26 | SP | 21 | SP | 21 | SP | 21 | SP | 21 |  |
| Q9S7Y7 | EUKARYA | SP | 27 | SP | 27 | SP | 27 | SP | 27 | SP | 27 | SP | 27 |  |
| P01543 | EUKARYA | SP | 27 | SP | 27 | SP | 27 | SP | 27 | SP | 27 | SP | 27 |  |
| P55057 | EUKARYA | SP | 27 | LIPO | 26 | NO_SP | 26 | SP | 27 | SP | 27 | SP | 26 |  |
| P00655 | EUKARYA | SP | 27 | SP | 28 | SP | 27 | SP | 27 | SP | 27 | SP | 19 |  |
| P01588 | EUKARYA | SP | 27 | SP | 27 | SP | 27 | SP | 27 | SP | 27 | SP | 27 |  |
| P19656 | EUKARYA | SP | 27 | SP | 27 | SP | 27 | SP | 27 | SP | 27 | SP | 27 |  |
| Q99372 | EUKARYA | SP | 27 | SP | 27 | SP | 27 | SP | 27 | SP | 27 | SP | 27 |  |
| P09791 | EUKARYA | SP | 27 | SP | 27 | SP | 27 | SP | 27 | SP | 27 | SP | 25 |  |
| P35858 | EUKARYA | SP | 27 | SP | 27 | SP | 27 | SP | 27 | SP | 27 | SP | 21 |  |
| P01295 | EUKARYA | SP | 27 | SP | 27 | SP | 27 | SP | 27 | SP | 27 | SP | 25 |  |
| Q969P0 | EUKARYA | SP | 27 | SP | 27 | SP | 27 | SP | 27 | SP | 27 | SP | 27 |  |
| Q6UXD5 | EUKARYA | SP | 27 | SP | 27 | SP | 27 | SP | 27 | SP | 27 | SP | 27 |  |
| B3F051 | EUKARYA | SP | 27 | SP | 27 | SP | 27 | SP | 27 | SP | 27 | SP | 27 |  |
| Q9WVJ9 | EUKARYA | SP | 27 | SP | 27 | SP | 27 | SP | 27 | SP | 27 | SP | 25 |  |
| P16611 | EUKARYA | SP | 27 | SP | 27 | SP | 27 | SP | 27 | SP | 27 | SP | 27 |  |
| Q942A7 | EUKARYA | SP | 27 | SP | 27 | SP | 27 | SP | 27 | SP | 22 | SP | 22 |  |
| O43291 | EUKARYA | SP | 27 | SP | 27 | SP | 27 | SP | 27 | SP | 27 | SP | 27 |  |
| Q16568 | EUKARYA | SP | 27 | SP | 27 | SP | 27 | SP | 27 | SP | 27 | SP | 27 |  |
| Q5FWE3 | EUKARYA | SP | 27 | SP | 27 | SP | 27 | SP | 27 | SP | 27 | SP | 27 |  |
| P62520 | EUKARYA | SP | 27 | SP | 27 | SP | 27 | SP | 19 | SP | 21 | SP | 19 |  |
| Q867W1 | EUKARYA | SP | 27 | SP | 27 | SP | 27 | SP | 27 | SP | 27 | SP | 27 |  |
| Q8MP00 | EUKARYA | SP | 27 | SP | 32 | SP | 32 | SP | 32 | SP | 32 | SP | 30 |  |
| Q10ST8 | EUKARYA | SP | 27 | SP | 27 | SP | 27 | LIPO | 17 | LIPO | 17 | SP | 18 |  |
| O35701 | EUKARYA | SP | 27 | NO_SP | -1 | NO_SP | -1 | SP | 27 | SP | 27 | SP | 27 |  |
| P62931 | EUKARYA | SP | 26 | SP | 26 | SP | 26 | SP | 26 | SP | 26 | SP | 26 |  |
| P17948 | EUKARYA | SP | 26 | SP | 26 | SP | 26 | SP | 26 | SP | 26 | SP | 25 |  |
| O70280 | EUKARYA | SP | 26 | SP | 26 | SP | 26 | SP | 26 | SP | 26 | SP | 26 |  |
| Q41015 | EUKARYA | SP | 26 | SP | 23 | SP | 23 | SP | 23 | SP | 23 | SP | 23 |  |
| P01538 | EUKARYA | SP | 26 | SP | 26 | SP | 26 | SP | 26 | SP | 26 | SP | 26 |  |
| P16235 | EUKARYA | SP | 26 | SP | 26 | SP | 26 | SP | 26 | SP | 26 | SP | 26 |  |
| P13204 | EUKARYA | SP | 26 | SP | 26 | SP | 26 | SP | 26 | SP | 26 | SP | 23 |  |
| P17454 | EUKARYA | SP | 26 | SP | 26 | SP | 26 | SP | 26 | SP | 26 | SP | 26 |  |
| P16860 | EUKARYA | SP | 26 | SP | 26 | SP | 26 | SP | 26 | SP | 26 | SP | 26 |  |
| P23472 | EUKARYA | SP | 26 | SP | 26 | SP | 26 | SP | 26 | SP | 26 | SP | 26 |  |
| Q6T6T4 | EUKARYA | SP | 26 | SP | 24 | SP | 25 | SP | 24 | SP | 24 | SP | 24 |  |
| P0C1X6 | EUKARYA | SP | 26 | SP | 26 | SP | 26 | SP | 26 | SP | 26 | SP | 26 |  |
| P28088 | EUKARYA | SP | 26 | LIPO | 18 | LIPO | 18 | SP | 26 | SP | 26 | SP | 23 |  |
| Q6DWK3 | EUKARYA | SP | 26 | SP | 27 | SP | 27 | SP | 27 | SP | 27 | SP | 21 |  |
| A9UGV7 | EUKARYA | SP | 26 | SP | 26 | SP | 26 | SP | 26 | SP | 26 | SP | 26 |  |
| Q6ASY2 | EUKARYA | SP | 26 | SP | 26 | SP | 26 | SP | 26 | SP | 28 | SP | 26 |  |
| O00175 | EUKARYA | SP | 26 | SP | 18 | SP | 18 | SP | 18 | SP | 18 | SP | 18 |  |
| Q16270 | EUKARYA | SP | 26 | SP | 28 | SP | 26 | SP | 26 | SP | 26 | SP | 28 |  |
| P01034 | EUKARYA | SP | 26 | SP | 26 | SP | 26 | SP | 26 | SP | 26 | SP | 26 |  |
| P01189 | EUKARYA | SP | 26 | SP | 26 | SP | 26 | SP | 26 | SP | 26 | SP | 26 |  |
| O95881 | EUKARYA | SP | 26 | SP | 26 | SP | 26 | SP | 26 | SP | 26 | SP | 26 |  |
| Q86YD3 | EUKARYA | SP | 26 | SP | 26 | SP | 26 | SP | 26 | SP | 26 | SP | 24 |  |
| Q9BZR6 | EUKARYA | SP | 26 | SP | 25 | SP | 25 | SP | 26 | SP | 26 | SP | 25 |  |
| P24807 | EUKARYA | SP | 26 | SP | 26 | SP | 26 | SP | 26 | SP | 26 | SP | 26 |  |
| Q6TPK4 | EUKARYA | SP | 26 | SP | 23 | SP | 23 | SP | 23 | SP | 23 | SP | 22 |  |
| Q7YZS9 | EUKARYA | SP | 26 | SP | 26 | SP | 26 | SP | 26 | SP | 26 | SP | 26 |  |
| Q86SC0 | EUKARYA | SP | 26 | SP | 24 | SP | 26 | SP | 24 | SP | 27 | SP | 22 |  |
| Q9U3Z3 | EUKARYA | SP | 26 | SP | 26 | SP | 26 | SP | 26 | SP | 26 | SP | 26 |  |
| Q8BSU2 | EUKARYA | SP | 26 | SP | 26 | SP | 26 | SP | 26 | SP | 32 | SP | 26 |  |
| P01342 | EUKARYA | SP | 26 | SP | 26 | SP | 26 | SP | 24 | SP | 25 | SP | 24 |  |
| P81019 | EUKARYA | SP | 25 | SP | -1 | SP | 25 | SP | 25 | SP | 25 | SP | 14 |  |
| Q9V496 | EUKARYA | SP | 25 | SP | 25 | SP | 25 | SP | 25 | SP | 25 | SP | 25 |  |
| Q62703 | EUKARYA | SP | 25 | SP | 25 | SP | 25 | SP | 25 | SP | 25 | SP | 25 |  |
| O04004 | EUKARYA | SP | 25 | SP | 26 | SP | 25 | SP | 25 | SP | 25 | SP | 25 |  |
| P00751 | EUKARYA | SP | 25 | SP | 21 | SP | 21 | SP | 25 | SP | 25 | SP | 25 |  |
| P13224 | EUKARYA | SP | 25 | SP | 25 | SP | 25 | SP | 22 | SP | 25 | SP | 25 |  |
| Q5UCC4 | EUKARYA | SP | 25 | SP | 27 | SP | 27 | SP | 25 | SP | 29 | SP | 25 |  |
| P07634 | EUKARYA | SP | 25 | SP | 25 | SP | 25 | SP | 25 | SP | 25 | SP | 25 |  |
| P11654 | EUKARYA | SP | 25 | SP | 25 | SP | 25 | SP | 25 | SP | 25 | SP | 25 |  |
| A0A161AT60 | EUKARYA | SP | 25 | SP | 25 | SP | 25 | SP | 25 | SP | 25 | SP | 25 |  |
| P14925 | EUKARYA | SP | 25 | SP | 25 | SP | 25 | SP | 25 | SP | 25 | SP | 23 |  |
| P21743 | EUKARYA | SP | 25 | SP | 25 | SP | 25 | SP | 25 | SP | 25 | SP | 24 |  |
| P29022 | EUKARYA | SP | 25 | SP | 25 | SP | 25 | SP | 25 | SP | 25 | SP | 25 |  |
| P22969 | EUKARYA | SP | 25 | SP | 25 | SP | 25 | SP | 25 | SP | 25 | SP | 24 |  |
| P03973 | EUKARYA | SP | 25 | SP | 25 | SP | 25 | SP | 25 | SP | 25 | SP | 25 |  |
| D7UQ40 | EUKARYA | SP | 25 | SP | 19 | SP | 19 | SP | 19 | SP | 19 | SP | 19 |  |
| P35572 | EUKARYA | SP | 25 | SP | 28 | SP | 28 | SP | 28 | SP | 28 | SP | 28 |  |
| Q5I2B2 | EUKARYA | SP | 25 | SP | 25 | SP | 25 | SP | 25 | SP | 25 | SP | 25 |  |
| P13725 | EUKARYA | SP | 25 | SP | 26 | SP | 26 | SP | 25 | SP | 25 | SP | 25 |  |
| P01730 | EUKARYA | SP | 25 | SP | 25 | SP | 25 | SP | 25 | SP | 25 | SP | 23 |  |
| P52798 | EUKARYA | SP | 25 | SP | 25 | SP | 25 | SP | 22 | SP | 25 | SP | 22 |  |
| Q99944 | EUKARYA | SP | 25 | SP | 25 | SP | 25 | SP | 26 | SP | 28 | SP | 25 |  |
| Q9Y5U5 | EUKARYA | SP | 25 | SP | 25 | SP | 25 | SP | 25 | SP | 25 | SP | 25 |  |
| Q14641 | EUKARYA | SP | 25 | SP | 25 | SP | 25 | SP | 25 | SP | 25 | SP | 25 |  |
| Q84UV8 | EUKARYA | SP | 25 | SP | 25 | SP | 25 | SP | 25 | SP | 25 | SP | 23 |  |
| Q86R84 | EUKARYA | SP | 25 | SP | 25 | SP | 25 | SP | 17 | LIPO | 14 | SP | 17 |  |
| Q8GTM0 | EUKARYA | SP | 25 | SP | 25 | SP | 25 | SP | 25 | SP | 25 | SP | 25 |  |
| Q29381 | EUKARYA | SP | 25 | SP | 25 | SP | 25 | SP | 25 | SP | 25 | SP | 25 |  |
| Q969Z4 | EUKARYA | SP | 25 | SP | 24 | SP | 24 | SP | 26 | SP | 25 | SP | 25 |  |
| Q8T0W3 | EUKARYA | SP | 25 | SP | 25 | SP | 25 | SP | 25 | SP | 25 | SP | 25 |  |
| Q27972 | EUKARYA | SP | 24 | SP | 23 | SP | 23 | SP | 23 | SP | 24 | SP | 23 |  |
| Q41350 | EUKARYA | SP | 24 | SP | 24 | SP | 24 | SP | 24 | SP | 24 | SP | 27 |  |
| P80747 | EUKARYA | SP | 24 | SP | 24 | SP | 24 | SP | 24 | SP | 24 | SP | 24 |  |
| P82818 | EUKARYA | SP | 24 | SP | 22 | LIPO | 18 | LIPO | 18 | LIPO | 18 | SP | 19 |  |
| P01348 | EUKARYA | SP | 24 | SP | 24 | SP | 24 | SP | 24 | SP | 24 | SP | 24 |  |
| P40967 | EUKARYA | SP | 24 | NO_SP | -1 | SP | 23 | SP | 23 | SP | 24 | SP | 22 |  |
| P11643 | EUKARYA | SP | 24 | SP | 28 | SP | 28 | SP | 29 | SP | 29 | SP | 25 |  |
| Q0P569 | EUKARYA | SP | 24 | SP | 24 | SP | 24 | SP | 24 | SP | 24 | SP | 24 |  |
| Q24825 | EUKARYA | SP | 24 | SP | 15 | SP | 15 | SP | 17 | SP | 17 | SP | 15 |  |
| P02712 | EUKARYA | SP | 24 | SP | 24 | SP | 24 | SP | 24 | SP | 24 | SP | 24 |  |
| P02701 | EUKARYA | SP | 24 | SP | 25 | SP | 24 | SP | 24 | SP | 24 | SP | 24 |  |
| P01297 | EUKARYA | SP | 24 | SP | 24 | SP | 24 | SP | 24 | SP | 24 | SP | 24 |  |
| P01070 | EUKARYA | SP | 24 | SP | 24 | SP | 24 | SP | 24 | SP | 24 | SP | 24 |  |
| P01005 | EUKARYA | SP | 24 | SP | 24 | SP | 24 | SP | 24 | SP | 24 | SP | 24 |  |
| P16882 | EUKARYA | SP | 24 | SP | 24 | SP | 24 | SP | 24 | SP | 24 | SP | 20 |  |
| P0DUS6 | EUKARYA | SP | 24 | SP | 22 | SP | 22 | SP | 22 | SP | 22 | SP | 22 |  |
| P03971 | EUKARYA | SP | 24 | SP | 27 | SP | 27 | SP | 24 | SP | 27 | SP | 24 |  |
| P10675 | EUKARYA | SP | 24 | SP | 24 | SP | 24 | SP | 24 | SP | 24 | SP | 24 |  |
| M1MR49 | EUKARYA | SP | 24 | SP | 24 | SP | 24 | SP | 24 | SP | 24 | NO_SP | -1 |  |
| I2G9B4 | EUKARYA | SP | 24 | SP | 24 | SP | 24 | SP | 24 | SP | 24 | SP | 24 |  |
| P85261 | EUKARYA | SP | 24 | SP | 24 | SP | 24 | SP | 24 | SP | 24 | SP | 23 |  |
| P38029 | EUKARYA | SP | 24 | SP | 24 | SP | 24 | SP | 24 | SP | 24 | SP | 24 |  |
| P27177 | EUKARYA | SP | 24 | SP | 24 | SP | 24 | SP | 24 | SP | 24 | SP | 18 |  |
| P04090 | EUKARYA | SP | 24 | SP | 24 | SP | 24 | SP | 24 | SP | 24 | SP | 22 |  |
| O95750 | EUKARYA | SP | 24 | SP | 21 | SP | 26 | SP | 22 | SP | 22 | SP | 22 |  |
| P35916 | EUKARYA | SP | 24 | SP | 24 | SP | 24 | SP | 24 | SP | 24 | SP | 24 |  |
| Q6UXH1 | EUKARYA | SP | 24 | SP | 24 | SP | 24 | SP | 24 | SP | 24 | SP | 24 |  |
| Q8N126 | EUKARYA | SP | 24 | SP | 24 | SP | 24 | SP | 24 | SP | 24 | SP | 24 |  |
| Q8N441 | EUKARYA | SP | 24 | SP | 24 | SP | 24 | SP | 25 | SP | 24 | SP | 24 |  |
| Q9UHD0 | EUKARYA | SP | 24 | SP | 24 | SP | 22 | SP | 22 | SP | 20 | SP | 18 |  |
| Q7Z5L0 | EUKARYA | SP | 24 | SP | 24 | SP | 24 | SP | 24 | SP | 24 | SP | 20 |  |
| Q6GU94 | EUKARYA | SP | 24 | SP | 24 | SP | 24 | SP | 24 | SP | 24 | SP | 24 |  |
| Q84ZX5 | EUKARYA | SP | 24 | SP | 24 | SP | 24 | SP | 24 | SP | 24 | SP | 24 |  |
| Q8MJV4 | EUKARYA | SP | 24 | SP | 24 | SP | 24 | SP | 24 | SP | 24 | SP | 24 |  |
| P81761 | EUKARYA | SP | 24 | SP | 24 | SP | 24 | SP | 24 | SP | 24 | SP | 24 |  |
| Q90W22 | EUKARYA | SP | 24 | SP | 24 | SP | 24 | SP | 24 | SP | 24 | SP | 24 |  |
| P35555 | EUKARYA | SP | 24 | SP | 24 | SP | 27 | SP | 24 | SP | 27 | SP | 24 |  |
| Q6UX46 | EUKARYA | SP | 24 | SP | 24 | SP | 24 | SP | 24 | SP | 24 | SP | 20 |  |
| O15467 | EUKARYA | SP | 23 | SP | 22 | SP | 22 | SP | 23 | SP | 25 | SP | 20 |  |
| Q15465 | EUKARYA | SP | 23 | SP | 23 | SP | 23 | SP | 21 | SP | 23 | SP | 21 |  |
| Q64385 | EUKARYA | SP | 23 | SP | 22 | SP | 22 | SP | 25 | SP | 25 | SP | 25 |  |
| O00585 | EUKARYA | SP | 23 | SP | 23 | SP | 23 | SP | 23 | SP | 23 | SP | 17 |  |
| O93233 | EUKARYA | SP | 23 | SP | 23 | SP | 23 | SP | 21 | SP | 21 | SP | 21 |  |
| O11780 | EUKARYA | SP | 23 | SP | 23 | SP | 23 | SP | 23 | SP | 23 | SP | 23 |  |
| Q92583 | EUKARYA | SP | 23 | SP | 23 | SP | 23 | SP | 23 | SP | 23 | SP | 23 |  |
| P07712 | EUKARYA | SP | 23 | SP | 23 | SP | 23 | SP | 23 | SP | 23 | SP | 21 |  |
| Q05186 | EUKARYA | SP | 23 | SP | 23 | SP | 23 | SP | 23 | SP | 23 | SP | 20 |  |
| P08989 | EUKARYA | SP | 23 | SP | 23 | SP | 23 | SP | 23 | SP | 23 | SP | 23 |  |
| P63153 | EUKARYA | SP | 23 | SP | 23 | SP | 23 | SP | 23 | SP | 23 | SP | 23 |  |
| P50448 | EUKARYA | SP | 23 | SP | 23 | SP | 23 | SP | 23 | SP | 23 | SP | 23 |  |
| Q5B428 | EUKARYA | SP | 23 | SP | 23 | SP | 23 | SP | 23 | SP | 23 | SP | 23 |  |
| P63152 | EUKARYA | SP | 23 | SP | 23 | SP | 23 | SP | 23 | SP | 23 | SP | 23 |  |
| P13909 | EUKARYA | SP | 23 | SP | 23 | SP | 23 | SP | 23 | SP | 23 | SP | 23 |  |
| Q9NPA0 | EUKARYA | SP | 23 | SP | 23 | SP | 23 | SP | 23 | SP | 23 | SP | 23 |  |
| P20241 | EUKARYA | SP | 23 | SP | 23 | SP | 23 | SP | 23 | SP | 23 | SP | 20 |  |
| P01011 | EUKARYA | SP | 23 | SP | 23 | SP | 23 | SP | 23 | SP | 23 | SP | 23 |  |
| P01364 | EUKARYA | SP | 23 | SP | 23 | SP | 23 | SP | 23 | SP | 23 | SP | 20 |  |
| P20155 | EUKARYA | SP | 23 | SP | 20 | SP | 20 | SP | 20 | SP | 20 | SP | 20 |  |
| Q13445 | EUKARYA | SP | 23 | SP | 23 | SP | 23 | SP | 23 | SP | 23 | SP | 23 |  |
| Q8MVA6 | EUKARYA | SP | 23 | SP | 22 | SP | 22 | SP | 22 | SP | 22 | SP | 22 |  |
| C0STK7 | EUKARYA | SP | 23 | SP | 23 | SP | 23 | SP | 21 | SP | 21 | SP | 21 |  |
| Q18184 | EUKARYA | SP | 23 | SP | 23 | SP | 23 | SP | 23 | SP | 23 | SP | 23 |  |
| Q61450 | EUKARYA | SP | 23 | SP | 23 | SP | 23 | NO_SP | -1 | SP | 23 | SP | 23 |  |
| Q1HAY7 | EUKARYA | SP | 23 | SP | 23 | NO_SP | 23 | SP | 23 | SP | 23 | SP | 22 |  |
| Q6ZBZ2 | EUKARYA | SP | 23 | SP | 23 | SP | 23 | SP | 23 | SP | 23 | SP | 23 |  |
| Q70LM3 | EUKARYA | SP | 23 | SP | 20 | SP | 20 | SP | 20 | SP | 25 | SP | 20 |  |
| P80370 | EUKARYA | SP | 23 | SP | 23 | SP | 23 | SP | 23 | SP | 23 | SP | 23 |  |
| Q8N6C8 | EUKARYA | SP | 23 | SP | 23 | SP | 23 | SP | 23 | SP | 23 | SP | 23 |  |
| Q96IQ7 | EUKARYA | SP | 23 | SP | 23 | SP | 23 | SP | 23 | SP | 23 | SP | 23 |  |
| Q76CA2 | EUKARYA | SP | 23 | SP | 23 | SP | 23 | SP | 23 | SP | 23 | SP | 23 |  |
| Q95NJ8 | EUKARYA | SP | 23 | SP | 23 | SP | 23 | SP | 23 | SP | 23 | SP | 23 |  |
| Q8MTX2 | EUKARYA | SP | 23 | SP | 23 | SP | 23 | SP | 23 | SP | 23 | SP | 21 |  |
| Q96HE7 | EUKARYA | SP | 23 | SP | 23 | SP | 23 | SP | 23 | SP | 23 | SP | 21 |  |
| Q09098 | EUKARYA | SP | 23 | SP | 23 | SP | 23 | SP | 23 | SP | 23 | SP | 23 |  |
| Q7Z1K3 | EUKARYA | SP | 23 | SP | 23 | SP | 23 | SP | 23 | SP | 23 | SP | 21 |  |
| A0A1S3PBB7 | EUKARYA | SP | 23 | SP | 23 | SP | 23 | SP | 23 | SP | 23 | SP | 21 |  |
| Q9PVW8 | EUKARYA | SP | 23 | SP | 23 | SP | 23 | SP | 23 | SP | 23 | SP | 23 |  |
| Q9Y258 | EUKARYA | SP | 23 | SP | 26 | SP | 23 | SP | 23 | SP | 23 | SP | 23 |  |
| P07981 | EUKARYA | SP | 22 | SP | 22 | SP | 22 | SP | 22 | SP | 22 | SP | 22 |  |
| P81264 | EUKARYA | SP | 22 | SP | 23 | SP | 23 | SP | 23 | SP | 23 | SP | 22 |  |
| Q00293 | EUKARYA | SP | 22 | SP | 22 | SP | 22 | SP | 22 | SP | 22 | NO_SP | -1 |  |
| P01347 | EUKARYA | SP | 22 | SP | 22 | SP | 22 | SP | 22 | SP | 22 | SP | 22 |  |
| P05155 | EUKARYA | SP | 22 | SP | 22 | SP | 22 | SP | 22 | SP | 22 | SP | 22 |  |
| P04275 | EUKARYA | SP | 22 | SP | 22 | SP | 22 | SP | 22 | SP | 22 | SP | 22 |  |
| P07457 | EUKARYA | SP | 22 | SP | 23 | SP | 22 | SP | 21 | SP | 21 | SP | 21 |  |
| Q12546 | EUKARYA | SP | 22 | SP | 20 | SP | 20 | SP | 20 | SP | 20 | SP | 20 |  |
| P48307 | EUKARYA | SP | 22 | SP | 24 | SP | 24 | SP | 24 | SP | 24 | SP | 22 |  |
| Q8N766 | EUKARYA | SP | 22 | SP | 21 | SP | -1 | SP | 21 | SP | 21 | SP | 21 |  |
| O60931 | EUKARYA | SP | 22 | SP | 22 | SP | 22 | SP | 22 | SP | 22 | SP | 20 |  |
| P14209 | EUKARYA | SP | 22 | SP | 20 | SP | 20 | SP | 20 | SP | 20 | SP | 21 |  |
| F1N5C8 | EUKARYA | SP | 22 | SP | 22 | SP | 22 | SP | 22 | SP | 22 | SP | 22 |  |
| I6S390 | EUKARYA | SP | 22 | SP | 22 | SP | 22 | SP | 22 | SP | 22 | SP | 22 |  |
| P20333 | EUKARYA | SP | 22 | SP | 22 | SP | 22 | SP | 22 | SP | 22 | SP | 22 |  |
| P21956 | EUKARYA | SP | 22 | SP | 22 | SP | 22 | SP | 22 | SP | 22 | SP | 22 |  |
| P19872 | EUKARYA | SP | 22 | SP | 22 | SP | 22 | SP | 22 | SP | 22 | SP | 22 |  |
| P26216 | EUKARYA | SP | 22 | SP | 22 | SP | 22 | SP | 22 | SP | 22 | SP | 22 |  |
| P10297 | EUKARYA | SP | 22 | SP | 22 | SP | 22 | SP | 22 | SP | 22 | SP | 22 |  |
| Q9TY95 | EUKARYA | SP | 22 | SP | 22 | SP | -1 | SP | 24 | SP | 22 | SP | 22 |  |
| P30438 | EUKARYA | SP | 22 | SP | 22 | SP | 22 | SP | 22 | SP | 22 | SP | 22 |  |
| P83472 | EUKARYA | SP | 22 | SP | 22 | SP | 22 | SP | 22 | SP | 22 | SP | 22 |  |
| P84809 | EUKARYA | SP | 22 | SP | 22 | SP | 22 | SP | 22 | SP | 22 | SP | 22 |  |
| Q60GF7 | EUKARYA | SP | 22 | SP | 22 | SP | 21 | SP | 21 | SP | 22 | SP | 21 |  |
| Q02763 | EUKARYA | SP | 22 | SP | 22 | SP | 22 | SP | 22 | SP | 22 | SP | 22 |  |
| Q86Y78 | EUKARYA | SP | 22 | SP | 25 | SP | 25 | SP | 22 | SP | 22 | SP | 19 |  |
| Q8NC67 | EUKARYA | SP | 22 | SP | 26 | SP | 26 | SP | 26 | SP | 26 | SP | 26 |  |
| Q92563 | EUKARYA | SP | 22 | SP | 22 | SP | 22 | SP | 22 | SP | 22 | SP | 22 |  |
| Q9BYZ8 | EUKARYA | SP | 22 | SP | 22 | SP | 22 | SP | 22 | SP | 22 | SP | 22 |  |
| Q9P0V8 | EUKARYA | SP | 22 | SP | 22 | SP | 22 | SP | 22 | SP | 22 | SP | 22 |  |
| Q6UWM5 | EUKARYA | SP | 22 | SP | 23 | SP | 23 | SP | 23 | SP | 23 | SP | 22 |  |
| Q8NBM8 | EUKARYA | SP | 22 | SP | 20 | SP | 20 | SP | 20 | SP | 20 | SP | 19 |  |
| Q8LD43 | EUKARYA | SP | 22 | SP | 22 | SP | 22 | SP | 22 | SP | 22 | SP | 22 |  |
| Q6F495 | EUKARYA | SP | 22 | SP | 22 | SP | 22 | SP | 22 | SP | 22 | SP | 22 |  |
| Q6IV20 | EUKARYA | SP | 22 | SP | 22 | SP | 22 | SP | 22 | SP | 22 | SP | 22 |  |
| Q6T6C2 | EUKARYA | SP | 22 | SP | 22 | SP | 22 | SP | 22 | SP | 22 | SP | 24 |  |
| P29027 | EUKARYA | SP | 22 | SP | 22 | SP | 22 | SP | 22 | SP | 22 | SP | 22 |  |
| P28800 | EUKARYA | SP | 22 | SP | 21 | SP | 21 | SP | 21 | SP | 21 | SP | 21 |  |
| P08236 | EUKARYA | SP | 22 | LIPO | 18 | SP | 25 | SP | 25 | SP | 22 | SP | 22 |  |
| Q8MP06 | EUKARYA | SP | 22 | SP | 24 | SP | 22 | SP | 22 | SP | 22 | SP | 22 |  |
| Q966V2 | EUKARYA | SP | 22 | LIPO | 21 | LIPO | 21 | LIPO | 21 | LIPO | 21 | NO_SP | -1 |  |
| Q9NL38 | EUKARYA | SP | 22 | SP | 22 | SP | 22 | SP | 22 | SP | 22 | SP | 22 |  |
| Q9TT95 | EUKARYA | SP | 22 | SP | 23 | SP | 23 | SP | 23 | SP | 22 | SP | 21 |  |
| P81999 | EUKARYA | SP | 22 | SP | 22 | SP | 22 | SP | 22 | SP | 22 | SP | 22 |  |
| Q07325 | EUKARYA | SP | 22 | SP | 22 | SP | 22 | SP | 22 | SP | 22 | SP | 22 |  |
| P55000 | EUKARYA | SP | 22 | SP | 22 | SP | 22 | SP | 22 | SP | 22 | SP | 22 |  |
| B3EWG0 | EUKARYA | SP | 21 | SP | 21 | SP | 21 | SP | 21 | SP | 21 | SP | 21 |  |
| P07529 | EUKARYA | SP | 21 | SP | 21 | SP | 21 | SP | 21 | SP | 21 | SP | 21 |  |
| O42807 | EUKARYA | SP | 21 | SP | 21 | SP | 21 | SP | 21 | SP | 21 | SP | 21 |  |
| P55773 | EUKARYA | SP | 21 | SP | 21 | SP | 21 | SP | 21 | SP | 21 | SP | 18 |  |
| O59896 | EUKARYA | SP | 21 | SP | 21 | SP | 21 | SP | 21 | SP | 21 | SP | 21 |  |
| Q05895 | EUKARYA | SP | 21 | SP | 22 | SP | 21 | SP | 21 | SP | 21 | SP | 21 |  |
| Q02747 | EUKARYA | SP | 21 | SP | 19 | SP | 19 | SP | 19 | SP | 19 | SP | 19 |  |
| P53366 | EUKARYA | SP | 21 | SP | 21 | SP | 21 | SP | 21 | SP | 19 | SP | 15 |  |
| Q26643 | EUKARYA | SP | 21 | SP | 21 | SP | 21 | SP | 21 | SP | 21 | SP | 21 |  |
| P01901 | EUKARYA | SP | 21 | SP | 21 | SP | 21 | SP | 21 | SP | 21 | SP | 21 |  |
| P02748 | EUKARYA | SP | 21 | SP | 21 | SP | 21 | SP | 21 | SP | 21 | SP | 20 |  |
| P04217 | EUKARYA | SP | 21 | SP | 21 | SP | 21 | SP | 21 | SP | 21 | SP | 21 |  |
| P07982 | EUKARYA | SP | 21 | SP | 21 | SP | 21 | SP | 21 | SP | 21 | SP | 21 |  |
| B0CPC6 | EUKARYA | SP | 21 | SP | 21 | SP | 21 | SP | 21 | SP | 21 | SP | 21 |  |
| P18632 | EUKARYA | SP | 21 | SP | 21 | SP | 21 | SP | 21 | SP | 21 | SP | 19 |  |
| A0A482A9N4 | EUKARYA | SP | 21 | SP | 21 | SP | 21 | SP | 21 | SP | 21 | SP | 20 |  |
| P02779 | EUKARYA | SP | 21 | SP | 21 | SP | 21 | SP | 21 | SP | 21 | SP | 21 |  |
| P0DUQ6 | EUKARYA | SP | 21 | SP | 21 | SP | 21 | SP | 21 | SP | 21 | SP | 21 |  |
| Q9Y6C2 | EUKARYA | SP | 21 | SP | 21 | SP | 21 | SP | 23 | SP | 21 | SP | 20 |  |
| P25116 | EUKARYA | SP | 21 | SP | 22 | NO_SP | 22 | SP | 22 | SP | 22 | NO_SP | -1 |  |
| Q2KJF1 | EUKARYA | SP | 21 | SP | 20 | SP | 20 | SP | 20 | SP | 20 | SP | 21 |  |
| Q9D6Y1 | EUKARYA | SP | 21 | SP | 21 | SP | 21 | SP | 21 | SP | 21 | SP | 19 |  |
| Q8C1Q4 | EUKARYA | SP | 21 | SP | 21 | SP | 21 | SP | 21 | SP | 21 | SP | 19 |  |
| Q5MIW7 | EUKARYA | SP | 21 | SP | 21 | SP | 21 | SP | 21 | SP | 21 | SP | 21 |  |
| Q99935 | EUKARYA | SP | 21 | SP | 21 | SP | 21 | SP | 21 | SP | 21 | SP | 21 |  |
| Q3UN54 | EUKARYA | SP | 21 | SP | 21 | SP | 21 | SP | 21 | SP | 21 | SP | 21 |  |
| Q8NJ73 | EUKARYA | SP | 21 | SP | 17 | SP | 17 | SP | 19 | SP | 17 | SP | 17 |  |
| Q9UBP4 | EUKARYA | SP | 21 | SP | 16 | SP | 22 | SP | 29 | SP | 16 | SP | 21 |  |
| P22455 | EUKARYA | SP | 21 | SP | 21 | SP | 21 | SP | 21 | SP | 21 | SP | 21 |  |
| P35590 | EUKARYA | SP | 21 | SP | 21 | SP | 21 | SP | 21 | SP | 21 | SP | 21 |  |
| Q99435 | EUKARYA | SP | 21 | SP | 21 | SP | 21 | SP | 21 | SP | 21 | SP | 21 |  |
| Q6UWM7 | EUKARYA | SP | 21 | SP | 21 | SP | 21 | SP | 22 | SP | 21 | SP | 21 |  |
| Q6UWE3 | EUKARYA | SP | 21 | SP | 22 | SP | 22 | SP | 22 | SP | 22 | SP | 18 |  |
| Q6E0U4 | EUKARYA | SP | 21 | SP | 21 | SP | 21 | SP | 21 | SP | 21 | SP | 21 |  |
| P58912 | EUKARYA | SP | 21 | SP | 20 | SP | 20 | SP | 20 | SP | 20 | SP | 20 |  |
| P11684 | EUKARYA | SP | 21 | SP | 21 | SP | 21 | SP | 21 | SP | 21 | SP | 21 |  |
| Q969N2 | EUKARYA | SP | 21 | SP | 21 | SP | 21 | SP | 23 | SP | 23 | SP | 21 |  |
| Q9GZM7 | EUKARYA | SP | 21 | SP | 21 | SP | 21 | SP | 21 | SP | 21 | SP | 21 |  |
| Q9GU69 | EUKARYA | SP | 21 | SP | 21 | SP | 21 | SP | 21 | SP | 21 | SP | 21 |  |
| Q6UWV6 | EUKARYA | SP | 21 | SP | 21 | SP | 21 | SP | 21 | SP | 21 | SP | 21 |  |
| B2NHN2 | EUKARYA | SP | 20 | SP | 20 | SP | 20 | SP | 22 | SP | 20 | SP | 22 |  |
| O35310 | EUKARYA | SP | 20 | SP | 20 | SP | 20 | SP | 20 | SP | 20 | SP | 20 |  |
| O48818 | EUKARYA | SP | 20 | SP | 20 | SP | 20 | SP | 20 | SP | 20 | SP | 20 |  |
| P80961 | EUKARYA | SP | 20 | SP | 20 | SP | 20 | SP | 20 | SP | 20 | SP | 20 |  |
| Q29143 | EUKARYA | SP | 20 | SP | 20 | SP | 20 | SP | 20 | SP | 20 | SP | 19 |  |
| P91745 | EUKARYA | SP | 20 | SP | 20 | SP | 20 | SP | 20 | SP | 20 | SP | 20 |  |
| P28825 | EUKARYA | SP | 20 | SP | 20 | SP | 20 | SP | 20 | SP | 20 | SP | 20 |  |
| P05090 | EUKARYA | SP | 20 | SP | 20 | SP | 20 | SP | 20 | SP | 20 | SP | 20 |  |
| P43497 | EUKARYA | SP | 20 | SP | 20 | SP | 20 | SP | 20 | SP | 20 | SP | 22 |  |
| Q95121 | EUKARYA | SP | 20 | SP | 19 | SP | 19 | SP | 19 | SP | 19 | SP | 17 |  |
| P02781 | EUKARYA | SP | 20 | SP | 20 | SP | 20 | SP | 20 | SP | 20 | SP | 20 |  |
| Q4WL81 | EUKARYA | SP | 20 | SP | 20 | SP | 20 | SP | 20 | SP | 20 | SP | 20 |  |
| A0A4P9I8G4 | EUKARYA | SP | 20 | SP | 20 | SP | 20 | SP | 20 | SP | 20 | SP | 20 |  |
| P10820 | EUKARYA | SP | 20 | SP | 20 | SP | 20 | SP | 20 | SP | 20 | SP | 20 |  |
| C0HKR1 | EUKARYA | SP | 20 | SP | 20 | SP | 20 | SP | 20 | SP | 20 | SP | 15 |  |
| P19835 | EUKARYA | SP | 20 | SP | 20 | SP | 20 | SP | 20 | SP | 20 | SP | 20 |  |
| P24802 | EUKARYA | SP | 20 | SP | 23 | SP | 23 | SP | 20 | SP | 23 | SP | 20 |  |
| Q71DJ5 | EUKARYA | SP | 20 | SP | 19 | SP | 21 | SP | 21 | SP | 21 | SP | 19 |  |
| A0A7E5WTY7 | EUKARYA | SP | 20 | SP | 20 | SP | 20 | SP | 20 | SP | 20 | SP | 20 |  |
| P26776 | EUKARYA | SP | 20 | SP | 20 | SP | 20 | SP | 20 | SP | 20 | SP | 20 |  |
| Q14210 | EUKARYA | SP | 20 | SP | 20 | SP | 20 | SP | 20 | SP | 20 | SP | 20 |  |
| P25311 | EUKARYA | SP | 20 | SP | 20 | SP | 20 | SP | 20 | SP | 20 | SP | 18 |  |
| O95994 | EUKARYA | SP | 20 | SP | 20 | SP | 20 | SP | 20 | SP | 20 | SP | 20 |  |
| Q12841 | EUKARYA | SP | 20 | SP | 20 | SP | 20 | SP | 20 | SP | 20 | SP | 20 |  |
| Q6UWI2 | EUKARYA | SP | 20 | SP | 20 | SP | 20 | SP | 20 | SP | 20 | SP | 20 |  |
| Q6UWL6 | EUKARYA | SP | 20 | SP | 20 | SP | 20 | SP | 20 | SP | 22 | SP | 20 |  |
| Q86Y34 | EUKARYA | SP | 20 | SP | 20 | SP | 20 | SP | 20 | SP | 22 | SP | 20 |  |
| Q8N4F0 | EUKARYA | SP | 20 | SP | 20 | SP | 20 | SP | 20 | SP | 20 | SP | 20 |  |
| Q8NAC3 | EUKARYA | SP | 20 | NO_SP | -1 | NO_SP | -1 | SP | 20 | SP | 20 | SP | 20 |  |
| Q96DR8 | EUKARYA | SP | 20 | SP | 19 | SP | 19 | SP | 19 | SP | 19 | SP | 19 |  |
| Q8HYA9 | EUKARYA | SP | 20 | SP | 20 | SP | 20 | SP | 20 | SP | 20 | SP | 20 |  |
| O60046 | EUKARYA | SP | 20 | SP | 20 | SP | 20 | SP | 20 | SP | 20 | SP | 20 |  |
| Q2U4L7 | EUKARYA | SP | 20 | SP | 20 | SP | 20 | SP | 20 | SP | 20 | SP | 20 |  |
| P06681 | EUKARYA | SP | 20 | SP | 18 | SP | 20 | SP | 20 | SP | 20 | SP | 18 |  |
| Q8MMH5 | EUKARYA | SP | 20 | SP | 20 | SP | 20 | SP | 20 | SP | 20 | SP | 20 |  |
| P25033 | EUKARYA | SP | 19 | SP | 18 | SP | 18 | SP | 18 | SP | 18 | SP | 18 |  |
| Q9VA83 | EUKARYA | SP | 19 | SP | 19 | SP | 19 | SP | 19 | SP | 19 | SP | 19 |  |
| Q52085 | EUKARYA | SP | 19 | SP | 19 | SP | 19 | SP | 19 | SP | 19 | SP | 19 |  |
| P34754 | EUKARYA | SP | 19 | SP | 27 | SP | 19 | SP | 19 | SP | 19 | SP | 19 |  |
| P33575 | EUKARYA | SP | 19 | SP | 19 | SP | 19 | SP | 19 | SP | 19 | SP | 19 |  |
| P00719 | EUKARYA | SP | 19 | SP | 19 | SP | 19 | SP | 19 | SP | 19 | SP | 19 |  |
| P02760 | EUKARYA | SP | 19 | SP | 19 | SP | 19 | SP | 19 | SP | 19 | SP | 19 |  |
| P01729 | EUKARYA | SP | 19 | SP | 19 | SP | 19 | SP | 19 | SP | 19 | SP | 19 |  |
| P0DUS7 | EUKARYA | SP | 19 | SP | 19 | SP | 19 | SP | 19 | SP | 19 | SP | 19 |  |
| P32004 | EUKARYA | SP | 19 | SP | 19 | SP | 19 | SP | 19 | SP | 19 | SP | 70 |  |
| P05623 | EUKARYA | SP | 19 | SP | 19 | SP | 19 | SP | 19 | SP | 19 | SP | 19 |  |
| Q62894 | EUKARYA | SP | 19 | SP | 19 | SP | 24 | SP | 19 | SP | 19 | SP | 19 |  |
| G4ZHR2 | EUKARYA | SP | 19 | SP | 19 | SP | 19 | SP | 19 | SP | 19 | SP | 19 |  |
| Q9GZZ8 | EUKARYA | SP | 19 | SP | 19 | SP | 19 | SP | 19 | SP | 19 | SP | 15 |  |
| P08887 | EUKARYA | SP | 19 | SP | 20 | SP | 19 | SP | 19 | SP | 19 | SP | 19 |  |
| P35775 | EUKARYA | SP | 19 | SP | 19 | SP | 19 | SP | 19 | SP | 19 | SP | 14 |  |
| Q14392 | EUKARYA | SP | 19 | SP | 17 | SP | 17 | SP | 17 | SP | 17 | SP | 17 |  |
| P25236 | EUKARYA | SP | 19 | SP | 19 | SP | 19 | SP | 19 | SP | 19 | SP | 19 |  |
| C0STK6 | EUKARYA | SP | 19 | SP | 19 | SP | 19 | SP | 19 | SP | 19 | SP | 17 |  |
| P16150 | EUKARYA | SP | 19 | SP | 19 | SP | 19 | SP | 19 | SP | 19 | SP | 19 |  |
| Q06KA2 | EUKARYA | SP | 19 | SP | 19 | SP | 19 | SP | 19 | SP | 19 | SP | 19 |  |
| Q30HU9 | EUKARYA | SP | 19 | SP | 19 | SP | 19 | SP | 19 | SP | 19 | SP | 19 |  |
| A0A7E5WUT2 | EUKARYA | SP | 19 | SP | 19 | SP | 19 | SP | 19 | SP | 19 | SP | 19 |  |
| Q8T1G4 | EUKARYA | SP | 19 | SP | 19 | SP | 19 | SP | 19 | SP | 19 | SP | 19 |  |
| P07093 | EUKARYA | SP | 19 | SP | 20 | SP | 20 | SP | 20 | SP | 20 | SP | 19 |  |
| P81605 | EUKARYA | SP | 19 | SP | 19 | SP | 19 | SP | 19 | SP | 19 | SP | 15 |  |
| Q01151 | EUKARYA | SP | 19 | SP | 19 | SP | 19 | SP | 19 | SP | 19 | SP | 19 |  |
| Q86YT9 | EUKARYA | SP | 19 | SP | 19 | SP | 19 | SP | 19 | SP | 19 | SP | 19 |  |
| Q9BQ51 | EUKARYA | SP | 19 | SP | 19 | SP | 19 | SP | 19 | SP | 19 | SP | 19 |  |
| P58294 | EUKARYA | SP | 19 | SP | 19 | SP | 19 | SP | 19 | SP | 19 | SP | 19 |  |
| P02788 | EUKARYA | SP | 19 | SP | 19 | SP | 19 | SP | 19 | SP | 19 | SP | 15 |  |
| Q70SU8 | EUKARYA | SP | 19 | SP | 19 | SP | 19 | SP | 19 | SP | 19 | SP | 16 |  |
| O43852 | EUKARYA | SP | 19 | SP | 19 | SP | 19 | SP | 19 | SP | 19 | SP | 17 |  |
| Q95UE8 | EUKARYA | SP | 19 | SP | 19 | SP | 19 | SP | 19 | SP | 19 | SP | 19 |  |
| P01068 | EUKARYA | SP | 19 | SP | 14 | SP | 22 | NO_SP | -1 | SP | 14 | SP | 70 |  |
| Q9NBW4 | EUKARYA | SP | 19 | SP | 19 | SP | 19 | SP | 19 | SP | 19 | SP | 19 |  |
| B0XT72 | EUKARYA | SP | 19 | SP | 25 | SP | 26 | SP | 25 | SP | 26 | SP | 19 |  |
| P81058 | EUKARYA | SP | 19 | SP | 19 | SP | 19 | SP | 19 | SP | 19 | SP | 19 |  |
| P20160 | EUKARYA | SP | 19 | SP | 19 | SP | 19 | SP | 19 | SP | 19 | SP | 19 |  |
| P47178 | EUKARYA | SP | 19 | SP | 19 | SP | 19 | SP | 19 | SP | 19 | SP | 19 |  |
| P81447 | EUKARYA | SP | 18 | SP | 18 | SP | 18 | SP | 18 | SP | 18 | SP | 16 |  |
| O96910 | EUKARYA | SP | 18 | SP | 18 | SP | 18 | SP | 18 | SP | 18 | SP | 18 |  |
| O62644 | EUKARYA | SP | 18 | SP | 18 | SP | 18 | SP | 18 | SP | 18 | SP | 18 |  |
| Q15762 | EUKARYA | SP | 18 | SP | 18 | SP | 18 | NO_SP | -1 | SP | 18 | SP | 18 |  |
| Q08380 | EUKARYA | SP | 18 | SP | 18 | SP | 18 | SP | 18 | SP | 18 | SP | 18 |  |
| P42255 | EUKARYA | SP | 18 | SP | 18 | SP | 18 | SP | 18 | SP | 18 | SP | 18 |  |
| P38574 | EUKARYA | SP | 18 | SP | 18 | SP | 18 | SP | 18 | SP | 18 | SP | 18 |  |
| Q09022 | EUKARYA | SP | 18 | SP | 18 | SP | 18 | SP | 18 | SP | 18 | SP | 18 |  |
| P49763 | EUKARYA | SP | 18 | SP | 21 | SP | 21 | SP | 20 | SP | 20 | SP | 18 |  |
| Q9VMD9 | EUKARYA | SP | 18 | SP | 18 | SP | 18 | SP | 18 | SP | 18 | SP | 18 |  |
| Q64424 | EUKARYA | SP | 18 | SP | 18 | SP | 18 | SP | 18 | SP | 18 | SP | 16 |  |
| P02647 | EUKARYA | SP | 18 | SP | 18 | SP | 18 | SP | 18 | SP | 18 | SP | 18 |  |
| P02652 | EUKARYA | SP | 18 | SP | 18 | SP | 18 | SP | 23 | SP | 18 | SP | 18 |  |
| Q5B1W7 | EUKARYA | SP | 18 | SP | 18 | SP | 18 | SP | 18 | SP | 18 | SP | 15 |  |
| P01045 | EUKARYA | SP | 18 | SP | 18 | SP | 18 | SP | 18 | SP | 18 | SP | 18 |  |
| P01048 | EUKARYA | SP | 18 | SP | 18 | SP | 18 | SP | 18 | SP | 18 | SP | 18 |  |
| P01042 | EUKARYA | SP | 18 | SP | 18 | SP | 18 | SP | 23 | SP | 23 | SP | 16 |  |
| P31025 | EUKARYA | SP | 18 | SP | 18 | SP | 18 | SP | 23 | SP | 23 | SP | 18 |  |
| Q7SCE9 | EUKARYA | SP | 18 | SP | 18 | SP | 18 | SP | 18 | SP | 18 | SP | 18 |  |
| L8BRS3 | EUKARYA | SP | 18 | SP | 18 | SP | 18 | SP | 18 | SP | 18 | SP | 18 |  |
| P49125 | EUKARYA | SP | 18 | SP | 18 | SP | 18 | SP | 18 | SP | 18 | SP | 18 |  |
| Q1HLC0 | EUKARYA | SP | 18 | SP | 18 | SP | 18 | SP | 18 | SP | 18 | SP | 18 |  |
| P22748 | EUKARYA | SP | 18 | SP | 19 | SP | 19 | SP | 19 | SP | 19 | SP | 19 |  |
| B9A8D7 | EUKARYA | SP | 18 | SP | 18 | SP | 18 | SP | 18 | SP | 18 | SP | 20 |  |
| P01833 | EUKARYA | SP | 18 | SP | 18 | SP | 18 | SP | 18 | SP | 18 | SP | 17 |  |
| Q6RG02 | EUKARYA | SP | 18 | SP | 18 | SP | 18 | SP | 18 | SP | 18 | SP | 18 |  |
| C5P3X6 | EUKARYA | SP | 18 | SP | 18 | SP | 18 | SP | 18 | SP | 25 | SP | 18 |  |
| P08176 | EUKARYA | SP | 18 | SP | 18 | SP | 18 | SP | 18 | SP | 18 | SP | 18 |  |
| Q02809 | EUKARYA | SP | 18 | SP | 18 | SP | 18 | SP | 20 | SP | 18 | SP | 18 |  |
| P32478 | EUKARYA | SP | 18 | SP | 18 | SP | 18 | SP | 18 | SP | 18 | SP | 18 |  |
| P43307 | EUKARYA | SP | 18 | SP | 32 | SP | 32 | SP | 21 | SP | 21 | SP | 23 |  |
| Q6UXG3 | EUKARYA | SP | 18 | SP | 18 | SP | 18 | SP | 18 | SP | 18 | SP | 18 |  |
| Q8NBJ9 | EUKARYA | SP | 18 | SP | 18 | SP | 18 | SP | 18 | SP | 18 | SP | 18 |  |
| E0X9F6 | EUKARYA | SP | 18 | SP | 16 | SP | 16 | SP | 16 | SP | 16 | SP | 16 |  |
| P81054 | EUKARYA | SP | 18 | SP | 18 | SP | 18 | SP | 18 | SP | 18 | SP | 18 |  |
| Q95NY5 | EUKARYA | SP | 18 | SP | 18 | SP | 18 | SP | 18 | SP | 18 | SP | 18 |  |
| P15522 | EUKARYA | SP | 18 | SP | 18 | SP | 18 | SP | 18 | SP | 18 | SP | 18 |  |
| Q9UBT3 | EUKARYA | SP | 18 | SP | 18 | SP | 18 | SP | 18 | SP | 18 | SP | 18 |  |
| P0CB51 | EUKARYA | SP | 17 | SP | 17 | SP | 17 | SP | 17 | SP | 17 | SP | 17 |  |
| P28797 | EUKARYA | SP | 17 | SP | 17 | SP | 17 | SP | 17 | SP | 17 | SP | 17 |  |
| P55058 | EUKARYA | SP | 17 | SP | 17 | SP | 17 | SP | 17 | SP | 17 | SP | 17 |  |
| Q00017 | EUKARYA | SP | 17 | SP | 17 | SP | 17 | SP | 17 | SP | 17 | SP | 17 |  |
| P00635 | EUKARYA | SP | 17 | SP | 17 | SP | 17 | SP | 17 | SP | 17 | SP | 17 |  |
| P06908 | EUKARYA | SP | 17 | SP | 17 | SP | 17 | SP | 17 | SP | 17 | SP | 17 |  |
| P13213 | EUKARYA | SP | 17 | SP | 17 | SP | 17 | SP | 17 | SP | 17 | SP | 17 |  |
| P0DUS8 | EUKARYA | SP | 17 | SP | 17 | SP | 17 | SP | 17 | SP | 17 | SP | 17 |  |
| P11069 | EUKARYA | SP | 17 | SP | 17 | SP | 17 | SP | 17 | SP | 17 | SP | 17 |  |
| P15358 | EUKARYA | SP | 17 | SP | 17 | SP | 17 | SP | 17 | SP | 17 | SP | 17 |  |
| P07994 | EUKARYA | SP | 17 | NO_SP | -1 | SP | 17 | SP | 17 | SP | 20 | SP | 17 |  |
| P15399 | EUKARYA | SP | 17 | SP | 17 | SP | 17 | SP | 17 | SP | 17 | SP | 14 |  |
| Q00802 | EUKARYA | SP | 17 | SP | 17 | SP | 17 | SP | 17 | SP | 17 | SP | 17 |  |
| P02877 | EUKARYA | SP | 17 | SP | 17 | SP | 17 | SP | 17 | SP | 17 | SP | 17 |  |
| P29457 | EUKARYA | SP | 17 | SP | 17 | SP | 17 | SP | 17 | SP | 17 | SP | 17 |  |
| Q50KB1 | EUKARYA | SP | 17 | SP | 17 | SP | 17 | SP | 17 | SP | 17 | SP | 17 |  |
| O95813 | EUKARYA | SP | 17 | SP | 17 | SP | 17 | SP | 17 | SP | 17 | SP | 17 |  |
| O95866 | EUKARYA | SP | 17 | SP | 17 | SP | 17 | SP | 17 | SP | 17 | SP | 15 |  |
| P14384 | EUKARYA | SP | 17 | SP | 17 | SP | 17 | SP | 17 | SP | 17 | SP | 17 |  |
| Q8WZI8 | EUKARYA | SP | 17 | SP | 17 | SP | 17 | SP | 17 | SP | 17 | SP | 17 |  |
| Q875I9 | EUKARYA | SP | 17 | SP | 17 | SP | 17 | SP | 17 | SP | 17 | SP | 17 |  |
| Q9NRM6 | EUKARYA | SP | 17 | SP | 17 | SP | 14 | SP | 14 | SP | 14 | SP | 12 |  |
| Q9NAX4 | EUKARYA | SP | 17 | SP | 17 | SP | 17 | SP | 17 | SP | 17 | SP | 17 |  |
| P80686 | EUKARYA | SP | 16 | SP | 16 | SP | -1 | SP | 16 | SP | 16 | SP | 16 |  |
| Q04457 | EUKARYA | SP | 16 | SP | 16 | SP | 16 | SP | 16 | SP | 16 | SP | 16 |  |
| P48842 | EUKARYA | SP | 16 | SP | 16 | SP | 16 | SP | 16 | SP | 16 | SP | 16 |  |
| P00773 | EUKARYA | SP | 16 | SP | 16 | SP | 16 | SP | 16 | SP | 16 | SP | 16 |  |
| P08056 | EUKARYA | SP | 16 | SP | 16 | SP | 16 | SP | 16 | SP | 16 | SP | 16 |  |
| D3Z9M3 | EUKARYA | SP | 16 | SP | 16 | SP | 16 | SP | 16 | SP | 16 | SP | 16 |  |
| Q9BXJ3 | EUKARYA | SP | 16 | SP | 16 | LIPO | 13 | SP | 16 | SP | 16 | SP | 14 |  |
| Q95JC9 | EUKARYA | SP | 16 | SP | 16 | SP | 16 | SP | 16 | SP | 16 | SP | 16 |  |
| Q7M418 | EUKARYA | SP | 16 | SP | 16 | SP | 16 | SP | 16 | SP | 16 | SP | 16 |  |
| Q5KSB7 | EUKARYA | SP | 16 | SP | 16 | SP | 16 | SP | 16 | SP | 16 | SP | 14 |  |
| Q6UX39 | EUKARYA | SP | 16 | SP | 16 | SP | 16 | SP | 16 | SP | 16 | SP | 16 |  |
| Q8TC27 | EUKARYA | SP | 16 | SP | 16 | SP | 16 | SP | 16 | SP | 16 | SP | 20 |  |
| Q6F4C6 | EUKARYA | SP | 16 | SP | 16 | SP | 16 | SP | 16 | SP | 16 | SP | 16 |  |
| Q5ECE3 | EUKARYA | SP | 16 | SP | 16 | SP | 16 | SP | 16 | SP | 16 | SP | 16 |  |
| Q2ULM2 | EUKARYA | SP | 16 | SP | 15 | SP | 22 | SP | 15 | SP | 15 | SP | 15 |  |
| Q9UVI4 | EUKARYA | SP | 16 | SP | 16 | SP | 16 | SP | 22 | SP | 16 | SP | 16 |  |
| P09230 | EUKARYA | SP | 15 | SP | 30 | SP | 30 | SP | 21 | SP | 41 | SP | 21 |  |
| Q94637 | EUKARYA | SP | 15 | SP | 15 | SP | 15 | SP | 15 | SP | 15 | SP | 15 |  |
| P13731 | EUKARYA | SP | 15 | SP | 15 | SP | 15 | SP | 15 | SP | 15 | SP | 15 |  |
| P27613 | EUKARYA | SP | 15 | SP | 15 | SP | 15 | SP | 15 | SP | 15 | SP | 13 |  |
| Q27650 | EUKARYA | SP | 13 | SP | 13 | SP | 13 | SP | 13 | SP | 13 | SP | 13 |  |
| O43653 | EUKARYA | SP | 11 | SP | 11 | SP | 11 | NO_SP | -1 | SP | 47 | SP | 70 |  |
| P39457 | EUKARYA | SP | 9 | NO_SP | -1 | NO_SP | -1 | NO_SP | -1 | NO_SP | -1 | NO_SP | -1 |  |
| P08943 | EUKARYA | SP | 6 | SP | 6 | SP | 6 | NO_SP | -1 | NO_SP | -1 | NO_SP | -1 |  |
| P02674 | EUKARYA | SP | 5 | NO_SP | -1 | NO_SP | -1 | NO_SP | -1 | NO_SP | -1 | NO_SP | -1 |  |
| Q6H9K0 | EUKARYA | SP | 5 | NO_SP | -1 | NO_SP | -1 | NO_SP | -1 | NO_SP | -1 | NO_SP | -1 |  |
| B3EWP4 | EUKARYA | SP | 32 | SP | 32 | SP | 32 | SP | 32 | SP | 32 | SP | 32 |  |
| P15500 | EUKARYA | SP | 3 | NO_SP | -1 | NO_SP | -1 | NO_SP | -1 | NO_SP | -1 | NO_SP | -1 |  |
| B3EWP3 | EUKARYA | SP | 25 | SP | 25 | SP | 25 | SP | 25 | SP | 25 | SP | 23 |  |
| P0C2W5 | EUKARYA | SP | 23 | SP | 22 | SP | 22 | SP | 22 | SP | 22 | SP | 22 |  |
| Q6KF82 | EUKARYA | SP | 23 | NO_SP | -1 | NO_SP | -1 | NO_SP | -1 | NO_SP | -1 | NO_SP | -1 |  |
| P01359 | EUKARYA | SP | 21 | NO_SP | -1 | NO_SP | -1 | NO_SP | -1 | NO_SP | -1 | NO_SP | -1 |  |
| P49252 | EUKARYA | SP | 18 | SP | 18 | SP | 18 | SP | 18 | SP | 18 | SP | 18 |  |
| P30122 | EUKARYA | SP | 18 | NO_SP | -1 | NO_SP | -1 | NO_SP | -1 | NO_SP | -1 | NO_SP | -1 |  |
| P52307 | EUKARYA | SP | 14 | SP | 15 | SP | 15 | SP | 14 | SP | 15 | SP | 13 |  |
| P16531 | EUKARYA | SP | 14 | SP | -1 | NO_SP | -1 | NO_SP | -1 | NO_SP | -1 | NO_SP | -1 |  |
| A1C3L3 | POSITIVE | SP | 85 | NO_SP | -1 | NO_SP | -1 | NO_SP | -1 | NO_SP | -1 | NO_SP | -1 |  |
| Q51705 | NEGATIVE | TAT | 57 | TAT | 57 | TAT | 57 | TAT | 57 | TAT | 57 | TAT | 27 |  |
| Q07982 | NEGATIVE | TAT | 52 | TAT | 52 | TAT | 52 | TAT | 52 | TAT | 58 | TAT | 59 |  |
| Q53728 | POSITIVE | SP | 47 | SP | 47 | SP | 47 | SP | 47 | SP | 47 | LIPO | 47 |  |
| P0DX29 | POSITIVE | SP | 47 | SP | 47 | SP | 49 | SP | 49 | SP | 49 | SP | 47 |  |
| Q8KRU5 | POSITIVE | TAT | 46 | TAT | 46 | TAT | 46 | TAT | 46 | TAT | 46 | TAT | 41 |  |
| Q8VPD4 | POSITIVE | TAT | 45 | TAT | 35 | TAT | 35 | TAT | 35 | TAT | 35 | TAT | 35 |  |
| P22637 | POSITIVE | TAT | 45 | TAT | 45 | TAT | 45 | TAT | 45 | TAT | 45 | TAT | 45 |  |
| P80561 | POSITIVE | SP | 45 | SP | 33 | SP | 33 | SP | 33 | SP | 33 | SP | 33 |  |
| P22088 | NEGATIVE | SP | 44 | LIPO | 15 | LIPO | 15 | LIPO | 15 | TAT | 40 | SP | 28 |  |
| P82594 | POSITIVE | TAT | 43 | TAT | 43 | TAT | 43 | TAT | 43 | TAT | 43 | TAT | 43 |  |
| Q0PCD7 | POSITIVE | TAT | 43 | TAT | 37 | TAT | 37 | TAT | 37 | TAT | 37 | TAT | 37 |  |
| Q69GM4 | NEGATIVE | TAT | 43 | TAT | 29 | TAT | 29 | TAT | 35 | TAT | 45 | TAT | 31 |  |
| P9WP41 | POSITIVE | TAT | 43 | TAT | 43 | TAT | 43 | TAT | 32 | SP | 34 | SP | 32 |  |
| P81594 | POSITIVE | TAT | 42 | TAT | 42 | TAT | 42 | TAT | 42 | TAT | 42 | TAT | 42 |  |
| O34213 | NEGATIVE | TAT | 42 | TAT | 42 | TAT | 42 | TAT | 42 | TAT | 42 | TAT | 42 |  |
| Q3ZAB8 | NEGATIVE | TAT | 42 | TAT | 34 | TAT | 34 | TAT | 34 | TAT | 34 | TAT | 34 |  |
| P00733 | POSITIVE | SP | 42 | SP | 27 | SP | 27 | SP | 27 | SP | 27 | SP | 27 |  |
| Q5LT66 | NEGATIVE | SP | 42 | SP | 42 | SP | 42 | SP | 42 | SP | 42 | SP | 42 |  |
| Q45340 | NEGATIVE | SP | 42 | SP | 42 | SP | 42 | SP | 42 | SP | 42 | SP | 40 |  |
| O86308 | POSITIVE | SP | 41 | TAT | 41 | TAT | 41 | SP | 41 | SP | 41 | SP | 41 |  |
| P26514 | POSITIVE | SP | 41 | SP | 41 | SP | 41 | SP | 41 | SP | 41 | SP | 41 |  |
| P50401 | POSITIVE | SP | 40 | SP | 40 | SP | 40 | SP | 40 | SP | 40 | SP | 40 |  |
| P25006 | NEGATIVE | TAT | 38 | TAT | 39 | TAT | 40 | TAT | 33 | TAT | 33 | TAT | 31 |  |
| P83544 | POSITIVE | SP | 38 | SP | 28 | SP | 28 | SP | 28 | SP | 28 | SP | 26 |  |
| Q9F437 | NEGATIVE | SP | 38 | SP | 22 | SP | 22 | SP | 22 | SP | 22 | SP | 20 |  |
| P81715 | POSITIVE | SP | 37 | SP | 30 | TAT | 30 | SP | 30 | SP | 30 | SP | 30 |  |
| P00260 | NEGATIVE | TAT | 37 | TAT | 37 | TAT | 37 | TAT | 37 | TAT | 37 | TAT | 37 |  |
| P27951 | POSITIVE | SP | 37 | SP | 37 | SP | 37 | SP | 37 | SP | 37 | SP | 37 |  |
| Q02834 | POSITIVE | TAT | 37 | TAT | 37 | TAT | 37 | TAT | 37 | TAT | 37 | SP | 37 |  |
| Q9L9Z9 | NEGATIVE | SP | 37 | SP | 37 | SP | 37 | SP | 37 | SP | 37 | SP | 37 |  |
| P22222 | POSITIVE | TAT | 36 | SP | 36 | TAT | 36 | SP | 36 | SP | 36 | SP | 36 |  |
| P39186 | NEGATIVE | SP | 35 | SP | 34 | SP | 34 | SP | 34 | SP | 34 | NO_SP | -1 |  |
| P46445 | NEGATIVE | SP | 35 | SP | 35 | SP | 35 | SP | 35 | SP | 35 | SP | 35 |  |
| P14529 | POSITIVE | SP | 35 | LIPO | 19 | LIPO | 19 | LIPO | 19 | SP | 20 | SP | 19 |  |
| Q980C7 | ARCHAEA | SP | 35 | SP | 30 | SP | 30 | SP | 30 | SP | 30 | SP | 30 |  |
| Q8RQU9 | POSITIVE | SP | 35 | SP | 35 | SP | 35 | SP | 35 | SP | 35 | SP | 35 |  |
| P0A3R9 | POSITIVE | SP | 34 | SP | 34 | SP | 34 | SP | 34 | SP | 35 | SP | 34 |  |
| Q93MW7 | POSITIVE | SP | 34 | SP | 29 | TAT | 29 | SP | 35 | SP | 29 | SP | 26 |  |
| P01551 | POSITIVE | SP | 33 | TAT | 33 | TAT | 33 | SP | 33 | SP | 33 | TAT | 33 |  |
| P36917 | POSITIVE | SP | 33 | SP | 33 | SP | 33 | SP | 33 | SP | 33 | SP | 33 |  |
| P15917 | POSITIVE | SP | 33 | SP | 33 | SP | 33 | SP | 33 | SP | 33 | SP | 33 |  |
| P9WIB9 | POSITIVE | SP | 33 | SP | 33 | SP | 33 | SP | 33 | SP | 33 | SP | 33 |  |
| P0CZ00 | POSITIVE | TAT | 32 | TAT | 32 | TAT | 32 | TAT | 32 | TAT | 32 | TAT | 22 |  |
| P12374 | NEGATIVE | TAT | 32 | TAT | 32 | TAT | 32 | TAT | 32 | TAT | 32 | TAT | 32 |  |
| P81186 | NEGATIVE | TAT | 32 | TAT | 32 | TAT | 32 | TAT | 32 | TAT | 32 | TAT | 32 |  |
| P9WP43 | POSITIVE | SP | 32 | SP | 32 | SP | 32 | SP | 32 | SP | 32 | SP | 29 |  |
| P37699 | POSITIVE | SP | 32 | SP | 32 | SP | 32 | SP | 32 | SP | 32 | SP | 32 |  |
| P19405 | POSITIVE | SP | 32 | SP | 32 | SP | 32 | SP | 32 | SP | 32 | SP | 32 |  |
| P14090 | POSITIVE | SP | 32 | SP | 32 | SP | 32 | SP | 32 | SP | 32 | SP | 32 |  |
| B7J3C9 | NEGATIVE | SP | 32 | SP | 32 | SP | 32 | SP | 32 | SP | 32 | SP | 32 |  |
| P94522 | POSITIVE | SP | 32 | SP | 32 | SP | 32 | SP | 32 | SP | 32 | SP | 32 |  |
| P01549 | POSITIVE | SP | 32 | SP | 32 | SP | 32 | SP | 30 | SP | 30 | SP | 25 |  |
| P26222 | POSITIVE | TAT | 31 | SP | 31 | SP | 31 | SP | 31 | SP | 31 | SP | 24 |  |
| D4GPK6 | ARCHAEA | TAT | 31 | TAT | 31 | TAT | 31 | TAT | 31 | TAT | 31 | TAT | 31 |  |
| Q51485 | NEGATIVE | SP | 31 | SP | 31 | SP | 15 | SP | 31 | SP | 31 | SP | 31 |  |
| P08750 | POSITIVE | SP | 31 | SP | 31 | SP | 31 | SP | 31 | SP | 31 | LIPO | 21 |  |
| P07883 | POSITIVE | TAT | 30 | TAT | 30 | TAT | 30 | TAT | 30 | TAT | 30 | TAT | 30 |  |
| Q06530 | NEGATIVE | TAT | 30 | TAT | 30 | TAT | 30 | TAT | 30 | TAT | 30 | TAT | 30 |  |
| P38059 | POSITIVE | SP | 30 | SP | 30 | SP | 30 | SP | 30 | SP | 30 | SP | 25 |  |
| P46883 | NEGATIVE | SP | 30 | SP | 30 | SP | 30 | SP | 30 | SP | 30 | SP | 30 |  |
| P01092 | POSITIVE | SP | 30 | SP | 30 | SP | 30 | SP | 30 | SP | 30 | SP | 30 |  |
| P11220 | POSITIVE | SP | 30 | SP | 30 | SP | 30 | SP | 30 | SP | 30 | SP | 30 |  |
| P18477 | POSITIVE | TAT | 30 | TAT | 30 | TAT | 30 | TAT | 33 | SP | 30 | TAT | 30 |  |
| P96729 | POSITIVE | SP | 30 | SP | 30 | SP | 30 | SP | 30 | SP | 30 | SP | 30 |  |
| P81549 | NEGATIVE | SP | 30 | SP | 30 | SP | 30 | SP | 30 | SP | 30 | SP | 30 |  |
| Q5Y818 | NEGATIVE | TAT | 29 | TAT | 29 | TAT | 29 | TAT | 29 | TAT | 29 | TAT | 29 |  |
| P33406 | NEGATIVE | SP | 29 | SP | 29 | SP | 29 | SP | 29 | SP | 29 | SP | 29 |  |
| P49051 | POSITIVE | SP | 29 | SP | 29 | SP | 29 | SP | 29 | SP | 29 | SP | 29 |  |
| P10475 | POSITIVE | SP | 29 | SP | 29 | SP | 29 | SP | 29 | SP | 29 | SP | 29 |  |
| P01077 | POSITIVE | SP | 29 | LIPO | 32 | SP | 29 | SP | 29 | SP | 29 | SP | 29 |  |
| P07662 | NEGATIVE | SP | 29 | SP | 27 | SP | 27 | SP | 27 | SP | 27 | SP | 25 |  |
| P32717 | NEGATIVE | SP | 29 | SP | 29 | SP | 29 | SP | 29 | SP | 29 | SP | 29 |  |
| P05695 | NEGATIVE | SP | 29 | SP | 29 | SP | 29 | SP | 29 | SP | 29 | SP | 29 |  |
| Q52VH2 | NEGATIVE | SP | 29 | SP | 29 | SP | 29 | SP | 29 | SP | 29 | SP | 29 |  |
| Q8KIL1 | NEGATIVE | SP | 29 | LIPO | 16 | LIPO | 16 | SP | 24 | LIPO | 16 | LIPO | 16 |  |
| Q8RQV0 | POSITIVE | SP | 29 | SP | 29 | SP | 29 | SP | 29 | SP | 29 | SP | 29 |  |
| P82593 | POSITIVE | SP | 29 | SP | 29 | SP | 29 | SP | 29 | SP | 29 | SP | 27 |  |
| P36649 | NEGATIVE | TAT | 28 | TAT | 28 | TAT | 28 | TAT | 28 | TAT | 28 | TAT | 28 |  |
| P07528 | POSITIVE | SP | 28 | SP | 28 | SP | 28 | SP | 29 | SP | 28 | SP | 28 |  |
| Q9S1G7 | NEGATIVE | SP | 28 | SP | 28 | SP | 28 | SP | 28 | SP | 28 | SP | 28 |  |
| Q44642 | NEGATIVE | SP | 28 | SP | 28 | SP | 28 | SP | 28 | SP | 28 | SP | 28 |  |
| P40943 | POSITIVE | SP | 28 | SP | 28 | SP | 28 | SP | 28 | SP | 28 | SP | 28 |  |
| Q06851 | POSITIVE | SP | 28 | SP | 28 | SP | 28 | SP | 28 | SP | 28 | SP | 28 |  |
| P23847 | NEGATIVE | SP | 28 | SP | 28 | SP | 28 | SP | 28 | SP | 28 | SP | 28 |  |
| A6QIG7 | POSITIVE | SP | 28 | SP | 28 | SP | 28 | SP | 28 | SP | 28 | SP | 28 |  |
| Q72EC8 | NEGATIVE | SP | 28 | LIPO | 22 | LIPO | 22 | LIPO | 22 | SP | 28 | LIPO | 16 |  |
| P0C0J0 | POSITIVE | SP | 27 | SP | 27 | SP | 27 | SP | 27 | SP | 27 | SP | 27 |  |
| P04369 | NEGATIVE | SP | 27 | SP | 27 | SP | 27 | SP | 27 | SP | 27 | SP | 27 |  |
| P22865 | POSITIVE | SP | 27 | SP | 27 | SP | 27 | SP | 27 | SP | 27 | SP | 27 |  |
| P21171 | POSITIVE | SP | 27 | SP | 27 | SP | 27 | SP | 27 | SP | 27 | SP | 20 |  |
| D3RVD4 | NEGATIVE | SP | 27 | SP | 27 | SP | 27 | SP | 27 | SP | 27 | SP | 27 |  |
| P27035 | POSITIVE | SP | 27 | SP | 27 | SP | 27 | SP | 27 | SP | 27 | SP | 21 |  |
| P33682 | POSITIVE | SP | 27 | SP | 27 | SP | 27 | SP | 27 | SP | 27 | SP | 27 |  |
| Q9KYH3 | POSITIVE | SP | 27 | SP | 27 | SP | 27 | SP | 27 | SP | 27 | SP | 27 |  |
| Q8RJP2 | NEGATIVE | SP | 27 | SP | 27 | SP | 27 | SP | 27 | SP | 27 | SP | 27 |  |
| Q5HEA4 | POSITIVE | SP | 27 | SP | 27 | SP | 27 | SP | 27 | SP | 27 | SP | 27 |  |
| O31737 | POSITIVE | SP | 27 | SP | 27 | SP | 27 | SP | 27 | SP | 27 | SP | 27 |  |
| P54507 | POSITIVE | SP | 27 | SP | -1 | SP | 27 | NO_SP | -1 | SP | 27 | SP | 27 |  |
| P0ADV1 | NEGATIVE | SP | 27 | SP | 27 | SP | 27 | SP | 27 | SP | 27 | SP | 27 |  |
| O29751 | ARCHAEA | TAT | 26 | TAT | 26 | TAT | 26 | TAT | 26 | TAT | 26 | TAT | 26 |  |
| O85465 | POSITIVE | SP | 26 | SP | 25 | SP | 25 | SP | 25 | SP | 26 | SP | 25 |  |
| P23843 | NEGATIVE | SP | 26 | SP | 26 | SP | 26 | SP | 26 | SP | 26 | SP | 26 |  |
| P0AEX9 | NEGATIVE | SP | 26 | SP | 26 | SP | 26 | SP | 26 | SP | 26 | SP | 24 |  |
| P45796 | POSITIVE | SP | 26 | SP | 26 | SP | 26 | SP | 26 | SP | 26 | SP | 26 |  |
| P09616 | POSITIVE | SP | 26 | SP | 26 | SP | 26 | SP | 26 | SP | 26 | SP | 26 |  |
| P16699 | POSITIVE | SP | 26 | SP | 31 | SP | 31 | SP | 31 | SP | 29 | SP | 29 |  |
| P84908 | POSITIVE | SP | 26 | SP | 26 | SP | 26 | SP | 26 | SP | 26 | SP | 26 |  |
| Q0P9X8 | NEGATIVE | SP | 26 | LIPO | 17 | SP | 26 | SP | 26 | SP | 26 | LIPO | 17 |  |
| P37735 | NEGATIVE | SP | 26 | SP | 26 | TAT | 26 | SP | 26 | SP | 26 | SP | 26 |  |
| Q9LAP7 | NEGATIVE | SP | 26 | SP | 26 | SP | 26 | SP | 26 | SP | 26 | SP | 24 |  |
| Q51372 | NEGATIVE | SP | 26 | SP | 26 | SP | 26 | SP | 26 | SP | 26 | SP | 26 |  |
| O05512 | POSITIVE | SP | 26 | SP | 21 | SP | 21 | SP | 26 | SP | 21 | SP | 21 |  |
| O07532 | POSITIVE | SP | 26 | SP | 26 | SP | 26 | SP | 26 | SP | 26 | SP | 26 |  |
| O05527 | NEGATIVE | SP | 25 | SP | 25 | SP | 25 | SP | 25 | SP | 25 | SP | 25 |  |
| P16700 | NEGATIVE | SP | 25 | SP | 25 | SP | 25 | SP | 25 | SP | 25 | SP | 25 |  |
| P45523 | NEGATIVE | SP | 25 | SP | 25 | SP | 25 | SP | 25 | SP | 25 | SP | 17 |  |
| O33599 | POSITIVE | SP | 25 | SP | 25 | SP | 25 | SP | 25 | SP | 25 | SP | 25 |  |
| P02925 | NEGATIVE | SP | 25 | SP | 25 | SP | 25 | SP | 25 | SP | 25 | SP | 23 |  |
| Q01269 | NEGATIVE | SP | 25 | SP | 25 | SP | 25 | SP | 25 | SP | 25 | SP | 25 |  |
| P18956 | NEGATIVE | SP | 25 | LIPO | 19 | SP | 25 | LIPO | 19 | SP | 25 | LIPO | 19 |  |
| P19487 | NEGATIVE | SP | 25 | SP | 25 | SP | 25 | SP | 25 | SP | 25 | SP | 19 |  |
| Q06529 | NEGATIVE | SP | 25 | SP | 25 | SP | 25 | SP | 25 | SP | 25 | SP | 25 |  |
| P00115 | NEGATIVE | SP | 25 | SP | 25 | SP | 25 | SP | 25 | SP | 25 | SP | 25 |  |
| P07497 | NEGATIVE | SP | 24 | SP | 24 | SP | 24 | SP | 24 | SP | 24 | SP | 24 |  |
| O05544 | NEGATIVE | SP | 24 | SP | 24 | SP | 24 | SP | 24 | SP | 24 | SP | 24 |  |
| P37329 | NEGATIVE | SP | 24 | SP | 24 | SP | 24 | SP | 24 | SP | 24 | SP | 24 |  |
| P0C1A9 | NEGATIVE | SP | 24 | SP | 24 | SP | 24 | SP | 24 | SP | 24 | SP | 24 |  |
| P94449 | POSITIVE | SP | 24 | SP | 24 | SP | 24 | SP | 24 | SP | 24 | SP | 24 |  |
| Q05918 | NEGATIVE | SP | 24 | SP | 24 | SP | 24 | SP | 24 | SP | 24 | SP | 22 |  |
| P04032 | NEGATIVE | SP | 24 | SP | 24 | SP | 24 | SP | 25 | SP | 25 | SP | 25 |  |
| V5YM14 | NEGATIVE | SP | 24 | SP | 24 | SP | 24 | SP | 24 | SP | 24 | SP | 24 |  |
| P09333 | POSITIVE | SP | 24 | SP | 24 | SP | 24 | SP | 24 | SP | 24 | SP | 24 |  |
| P19369 | NEGATIVE | SP | 24 | SP | 24 | SP | 24 | SP | 24 | SP | 24 | NO_SP | -1 |  |
| Q82S91 | NEGATIVE | SP | 24 | SP | 24 | SP | 24 | SP | 24 | SP | 24 | SP | 24 |  |
| Q8GFE2 | POSITIVE | SP | 24 | SP | 24 | SP | 24 | SP | 24 | SP | 36 | SP | 24 |  |
| Q02114 | POSITIVE | SP | 24 | SP | 24 | SP | 24 | SP | 24 | SP | 24 | SP | 24 |  |
| Q9L5A4 | NEGATIVE | SP | 24 | SP | 24 | SP | 24 | SP | 24 | SP | 24 | SP | 24 |  |
| Q9I2C5 | NEGATIVE | SP | 24 | SP | 24 | SP | 24 | SP | 24 | SP | 24 | SP | 24 |  |
| Q9RUE8 | POSITIVE | SP | 24 | SP | 22 | SP | 22 | SP | 22 | SP | 22 | SP | 22 |  |
| P31554 | NEGATIVE | SP | 24 | SP | 24 | SP | 24 | SP | 24 | SP | 24 | SP | 24 |  |
| Q9X7I4 | NEGATIVE | SP | 23 | SP | 23 | SP | 23 | SP | 23 | SP | 23 | SP | 23 |  |
| O07834 | NEGATIVE | SP | 23 | SP | 23 | SP | 23 | SP | 23 | SP | 23 | SP | 23 |  |
| P35482 | NEGATIVE | SP | 23 | SP | 23 | SP | 23 | SP | 23 | SP | 23 | SP | 21 |  |
| Q03011 | NEGATIVE | SP | 23 | SP | 23 | SP | 23 | SP | 23 | SP | 23 | SP | 23 |  |
| P07254 | NEGATIVE | SP | 23 | SP | 23 | SP | 23 | SP | 23 | SP | 23 | SP | 20 |  |
| P00120 | NEGATIVE | SP | 23 | SP | 23 | SP | 23 | SP | 23 | SP | 23 | SP | 23 |  |
| W8FLH9 | NEGATIVE | SP | 23 | SP | 23 | SP | 23 | SP | 23 | SP | 23 | SP | 23 |  |
| P0ABW7 | NEGATIVE | SP | 23 | SP | 23 | SP | 23 | SP | 23 | SP | 23 | SP | 23 |  |
| Q47223 | NEGATIVE | SP | 23 | SP | 23 | SP | 23 | SP | 23 | SP | 23 | SP | 21 |  |
| K4LAH1 | NEGATIVE | SP | 23 | SP | 23 | SP | 23 | SP | 23 | SP | 23 | SP | 21 |  |
| Q47706 | NEGATIVE | SP | 23 | SP | 23 | SP | 23 | SP | 23 | SP | 23 | SP | 23 |  |
| P14756 | NEGATIVE | SP | 23 | SP | 23 | SP | 23 | SP | 23 | SP | 23 | SP | 23 |  |
| Q9KK91 | NEGATIVE | SP | 23 | SP | 23 | SP | 23 | SP | 23 | SP | 23 | SP | 23 |  |
| P35755 | NEGATIVE | SP | 23 | SP | 23 | SP | 23 | SP | 23 | SP | 23 | SP | 23 |  |
| P44526 | NEGATIVE | SP | 23 | SP | 23 | SP | 23 | SP | 23 | SP | 23 | SP | 23 |  |
| P44737 | NEGATIVE | SP | 23 | SP | 23 | SP | 23 | SP | 23 | SP | 23 | SP | 23 |  |
| P59206 | POSITIVE | SP | 23 | SP | 23 | SP | 23 | SP | 23 | SP | 23 | SP | 23 |  |
| P15363 | NEGATIVE | LIPO | 23 | LIPO | 23 | LIPO | 23 | LIPO | 23 | LIPO | 23 | LIPO | 21 |  |
| I0J1I6 | NEGATIVE | SP | 23 | SP | 23 | SP | 23 | SP | 23 | SP | 23 | SP | 23 |  |
| Q9HVD1 | NEGATIVE | SP | 23 | SP | 23 | SP | 23 | SP | 23 | SP | 23 | SP | 21 |  |
| Q5SK82 | POSITIVE | TAT | 22 | TAT | 29 | TAT | 29 | TAT | 29 | TAT | 29 | TAT | 29 |  |
| P37921 | NEGATIVE | SP | 22 | SP | 22 | SP | 22 | SP | 22 | SP | 22 | SP | 22 |  |
| P77348 | NEGATIVE | SP | 22 | SP | 22 | SP | 22 | SP | 22 | SP | 22 | SP | 22 |  |
| P0AEU0 | NEGATIVE | SP | 22 | SP | 22 | SP | 22 | SP | 22 | SP | 22 | SP | 22 |  |
| P09551 | NEGATIVE | SP | 22 | SP | 22 | SP | 22 | SP | 22 | SP | 22 | SP | 22 |  |
| Q04681 | NEGATIVE | SP | 22 | SP | 22 | SP | 22 | SP | 22 | SP | 22 | SP | 22 |  |
| P80672 | NEGATIVE | SP | 22 | SP | 22 | SP | 22 | SP | 22 | SP | 22 | SP | 22 |  |
| P00101 | NEGATIVE | SP | 22 | SP | 22 | SP | 22 | SP | 22 | SP | 22 | SP | 16 |  |
| P0A0Y3 | NEGATIVE | SP | 22 | SP | 22 | SP | 22 | SP | 22 | SP | 22 | SP | 22 |  |
| P0AEQ3 | NEGATIVE | SP | 22 | SP | 22 | SP | 22 | SP | 22 | SP | 22 | SP | 22 |  |
| A0A0A1H8I4 | NEGATIVE | SP | 22 | SP | 22 | SP | 22 | SP | 22 | SP | 22 | SP | 22 |  |
| P13430 | NEGATIVE | SP | 22 | SP | 22 | SP | 22 | SP | 22 | SP | 22 | SP | 19 |  |
| P15488 | NEGATIVE | SP | 22 | SP | 22 | SP | 22 | SP | 22 | SP | 22 | SP | 22 |  |
| P19926 | NEGATIVE | SP | 22 | SP | 22 | SP | 22 | SP | 22 | SP | 22 | SP | 22 |  |
| P22340 | NEGATIVE | SP | 22 | SP | 22 | SP | 22 | SP | 22 | SP | 22 | SP | 20 |  |
| Q99PX1 | NEGATIVE | SP | 22 | SP | 22 | SP | 22 | SP | 22 | SP | 22 | SP | 22 |  |
| P00099 | NEGATIVE | SP | 22 | SP | 22 | SP | 22 | SP | 22 | SP | 22 | SP | 22 |  |
| P38540 | NEGATIVE | SP | 22 | SP | 22 | SP | 22 | SP | 22 | SP | 22 | SP | 22 |  |
| Q8RS40 | NEGATIVE | SP | 22 | SP | 22 | SP | 22 | SP | 22 | SP | 22 | SP | 15 |  |
| W5JXD7 | NEGATIVE | SP | 22 | SP | 22 | SP | 22 | SP | 22 | SP | 22 | SP | 21 |  |
| P0C8Z8 | NEGATIVE | SP | 22 | SP | 22 | SP | 22 | SP | 22 | SP | 22 | SP | 22 |  |
| Q8RMH6 | NEGATIVE | LIPO | 22 | LIPO | 22 | LIPO | 22 | LIPO | 22 | LIPO | 22 | LIPO | 22 |  |
| P76172 | NEGATIVE | SP | 21 | SP | 21 | SP | 21 | SP | 21 | SP | 21 | SP | 21 |  |
| P77747 | NEGATIVE | SP | 21 | SP | 21 | SP | 21 | SP | 21 | SP | 21 | SP | 21 |  |
| P48982 | NEGATIVE | SP | 21 | SP | 21 | SP | 21 | SP | 22 | SP | 21 | SP | 21 |  |
| P02970 | NEGATIVE | SP | 21 | SP | 21 | SP | 21 | SP | 21 | SP | 21 | SP | 21 |  |
| P00149 | NEGATIVE | SP | 21 | SP | 21 | SP | 21 | SP | 21 | SP | 21 | SP | 21 |  |
| P08191 | NEGATIVE | SP | 21 | SP | 21 | SP | 21 | SP | 19 | SP | 21 | SP | 19 |  |
| A0A1Z4F647 | NEGATIVE | SP | 21 | SP | 21 | SP | 21 | SP | 21 | SP | 21 | SP | 18 |  |
| P12061 | NEGATIVE | SP | 21 | SP | 21 | SP | 21 | SP | 21 | LIPO | 15 | SP | 15 |  |
| P0AD70 | NEGATIVE | SP | 21 | SP | 21 | SP | 21 | SP | 21 | SP | 21 | SP | 21 |  |
| P20041 | NEGATIVE | SP | 21 | SP | 21 | SP | 21 | SP | 21 | SP | 21 | SP | 21 |  |
| A0A2S4MYF8 | NEGATIVE | SP | 21 | SP | 21 | SP | 21 | SP | 21 | SP | 21 | SP | 21 |  |
| P21982 | NEGATIVE | SP | 21 | SP | 21 | SP | 21 | SP | 21 | SP | 21 | SP | 21 |  |
| P24093 | NEGATIVE | SP | 21 | SP | 21 | SP | 21 | SP | 21 | SP | 21 | SP | 21 |  |
| A0RZH5 | NEGATIVE | SP | 21 | SP | 21 | SP | 21 | SP | 21 | SP | 21 | SP | 21 |  |
| P33219 | NEGATIVE | SP | 21 | LIPO | 15 | LIPO | 15 | SP | 21 | LIPO | 15 | SP | 15 |  |
| A9ZM27 | NEGATIVE | SP | 21 | SP | 21 | SP | 21 | SP | 21 | SP | 21 | SP | 21 |  |
| P32823 | NEGATIVE | SP | 21 | SP | 21 | SP | 21 | SP | 21 | SP | 21 | SP | 21 |  |
| P36560 | NEGATIVE | SP | 21 | SP | 21 | SP | 21 | SP | 21 | SP | 21 | SP | 21 |  |
| P39116 | POSITIVE | SP | 21 | SP | 21 | SP | 21 | SP | 21 | SP | 21 | SP | 21 |  |
| P45996 | NEGATIVE | SP | 21 | SP | 21 | SP | 21 | SP | 21 | SP | 21 | SP | 21 |  |
| P38399 | NEGATIVE | SP | 21 | SP | 21 | SP | 21 | SP | 21 | SP | 21 | SP | 21 |  |
| A0A2S4N3N0 | NEGATIVE | SP | 21 | SP | 21 | SP | 21 | SP | 21 | SP | 21 | SP | 21 |  |
| P0A855 | NEGATIVE | SP | 21 | SP | 21 | SP | 21 | SP | 21 | SP | 21 | SP | 21 |  |
| P0AEG6 | NEGATIVE | SP | 20 | SP | 20 | SP | 20 | SP | 20 | SP | 20 | SP | 20 |  |
| Q04064 | NEGATIVE | SP | 20 | SP | 20 | SP | 20 | SP | 20 | SP | 20 | SP | 20 |  |
| P81894 | NEGATIVE | SP | 20 | SP | 20 | SP | 20 | SP | 20 | SP | 20 | SP | 20 |  |
| P30860 | NEGATIVE | SP | 19 | SP | 19 | SP | 19 | SP | 19 | SP | 19 | SP | 19 |  |
| P30859 | NEGATIVE | SP | 19 | SP | 19 | SP | 19 | SP | 19 | SP | 19 | SP | 19 |  |
| P00148 | NEGATIVE | SP | 19 | SP | 19 | SP | 19 | SP | 19 | SP | 19 | SP | 19 |  |
| P06111 | NEGATIVE | SP | 19 | SP | 19 | SP | 19 | SP | 19 | SP | 19 | SP | 19 |  |
| P29739 | NEGATIVE | SP | 19 | SP | 19 | SP | 19 | SP | 19 | SP | 19 | SP | 19 |  |
| P0C0T5 | NEGATIVE | SP | 19 | SP | 19 | SP | 19 | SP | 19 | SP | 19 | SP | 19 |  |
| A2TJI4 | NEGATIVE | SP | 19 | SP | 19 | SP | 19 | SP | 19 | SP | 19 | SP | 19 |  |
| P13113 | NEGATIVE | SP | 19 | SP | 19 | SP | 19 | SP | 19 | SP | 19 | SP | 19 |  |
| P32520 | NEGATIVE | SP | 19 | SP | 19 | SP | 19 | SP | 19 | SP | 19 | SP | 17 |  |
| O25825 | NEGATIVE | SP | 19 | SP | 19 | SP | 19 | SP | 19 | SP | 19 | SP | 24 |  |
| P36655 | NEGATIVE | SP | 19 | SP | 19 | SP | 19 | SP | 19 | SP | 19 | SP | 19 |  |
| P15452 | NEGATIVE | SP | 18 | SP | 18 | SP | 18 | SP | 18 | SP | 18 | SP | 18 |  |
| P77202 | NEGATIVE | SP | 17 | SP | 17 | SP | 17 | SP | 17 | SP | 17 | SP | 17 |  |
| Q5SME3 | POSITIVE | SP | 17 | SP | 17 | SP | 17 | SP | 19 | SP | 19 | SP | 19 |  |
| P0A1N4 | NEGATIVE | SP | 16 | SP | 16 | SP | 16 | SP | 16 | SP | 16 | SP | 16 |  |
| P08594 | POSITIVE | SP | 14 | LIPO | 18 | LIPO | 18 | LIPO | 18 | LIPO | 18 | LIPO | 18 |  |
| Q50833 | ARCHAEA | SP | 23 | SP | 23 | SP | 23 | SP | 23 | SP | 23 | SP | 18 |  |
